# Robust leishmanicidal upshot of some new diphenyl triazine-based molecules†

**DOI:** 10.1039/d4ra01904k

**Published:** 2024-07-17

**Authors:** Anju Singh, Mirza Adil Beg, Samra Jamal, Arif Khan, Abdur Rahman, Angamuthu Selvapandiyan, Syed Shafi, Nasimul Hoda

**Affiliations:** a Department of Chemistry, Drug Design and Synthesis Lab., Jamia Millia Islamia Jamia Nagar New Delhi 110025 India nhoda@jmi.ac.in +0091-11-26985507 +0091-9910200655; b Department of Molecular Medicine, Jamia Hamdard New Delhi 110062 India; c Department of Biotechnology, Jamia Hamdard New Delhi 110062 India; d Department of Chemistry, SCLS, Jamia Hamdard New Delhi 110062 India

## Abstract

Amongst the neglected tropical diseases, leishmaniasis alone causes 30 000 deaths annually due to the protozoan parasite genus *Leishmania*. Existing therapies have serious drawbacks in safety, drug resistance, field-adapted application and cost. Therefore, new safer and shorter treatments are an urgent need of the time. Herein, we report the synthesis of fifteen novel diphenyl triazine and diphenyl triazine pyrimidine derivatives and their antileishmanial properties against *Leishmania donovani*, that causes fatal visceral leishmaniasis. Most of the synthesized analogues exhibited more than 90% inhibition against the promastigote stage of the parasite. Moreover, compounds T4 and T7 showed potent activity against extracellular promastigote (IC_50_ = 1.074 μM and IC_50_ = 1.158 μM) as compared to miltefosine (IC_50_ = 1.477 μM) and is nontoxic towards the host THP-1 macrophage cell line. Interestingly, compound T4 exhibited significant activity against amastigotes (7.186 μM) and induced the macrophages to prevent the survival of the parasite. Our results indicate that T4 represents a new structural lead for this serious and neglected disease.

## Abstract 2

We report the synthesis of fifteen novel diphenyl triazine and diphenyl triazine pyrimidine derivatives and their antileishmanial properties. against *Leishmania donovani*.

## Graphical abstract

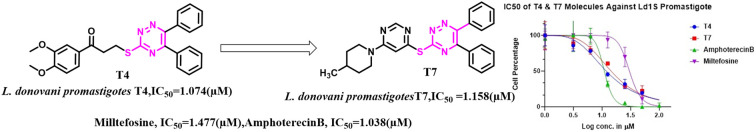

## 1. Introduction

Leishmaniasis is an endemic protozoan disease and is one of the major health problems worldwide. It is caused by the genus *Leishmania* transmitted from infected persons to normal ones by the bite of female phlebotomine sand flies.^1,2^ It appears in three clinical forms: cutaneous (CL), mucocutaneous (MCL), and visceral leishmaniasis (VL).^3,4^ VL^5^ is the life threatening form of the disease due to the failure of the host immune system.^6^ As per reports, 200 million people reside in leishmaniasis endemic areas and at least 2 million cases are reported every year with leishmaniasis causing 20 000–30 000 deaths.^7,8^

An absolute treatment for this ailment is still lacking, which makes the quest for the discovery of new potential antileishmanial agents. Amphotericin B (Ampho B), a well-known antifungal drug works as an essential medication for leishmaniasis.^1^ In addition to it, other available antileishmanial drugs comprise: miltefosine (MTF), paromomycin (P) and antimonials (Sb(iii)).^9^ Due to the increasing resistance and prolonged treatment of existing drugs, there is an immense need for the development of safer antileishmanial agents.

**Fig. 1:**
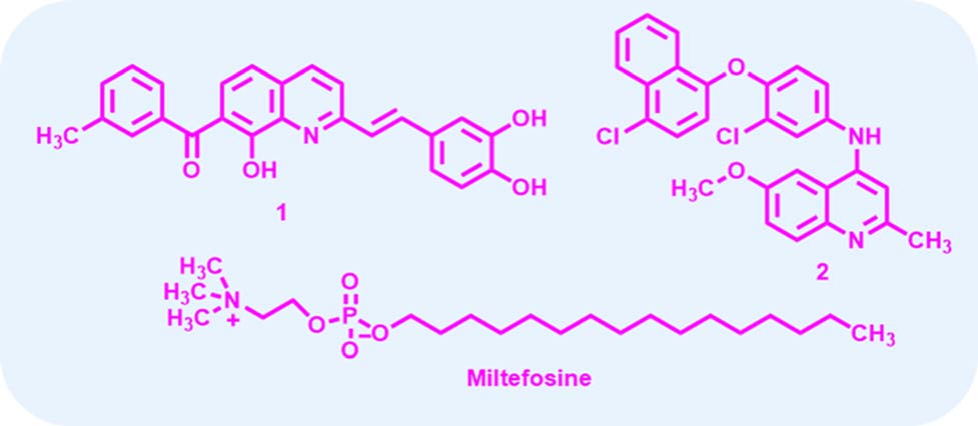
Structure of some known antileishmanial drugs.^10^

Among the scaffolds explored, quinoline derivatives have shown promising biological activities against various disease-causing parasites. Compound 1 (Fig. 1) passed in the Drugs for Neglected Diseases initiative (DNDi) pipeline for *in vivo* testing due to their better selectivity index as compared to MTF.^10^ Recently, the complex quinaldine derivative 2 (Fig. 1) was designed through virtual screening methods to inhibit infantum type 2 NADH dehydrogenase (NDH2). It experimentally proved the inhibition of the enzyme and displayed notable activity against *L. infantum* axenic amastigotes and promastigotes.^11^

**Fig. 2:**
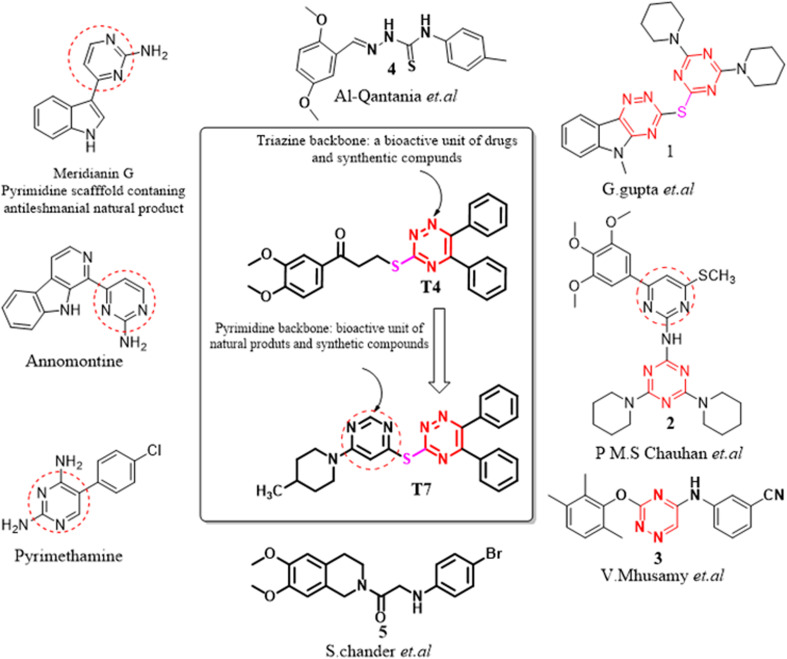
Designing of diphenyl triazine, pyrimidine-based hybrids showing antileishmanial activity.

The potentiality of 1,3,5-triazine derivatives against leishmaniasis has been reported by several research groups.^12–16^ Triazine core moiety linked through an ether linkage of pentamidine resulted in the development of two antileishmanial compounds 2 (Fig. 2) with good activity against intracellular *L. donovani* amastigotes.^16^ Analogues of β-carboline-1,3,5-triazine have been reported to act against promastigote and amastigote forms of *Leishmania amazonensis*.^17^ 1,3,4-thiadiazole analogues like compound 4 (Fig. 2) also showed very capable antileishmanial effects even at lower concentrations.^18^ 1,2,4-triazino[5,6-*b*]indol-3-ylthio-1,3,5-triazine 1 (Fig. 2) displayed more than 90% inhibition against promastigote form of *L. donovani*.^17^ It was found to be the most active and least toxic with 20 and 10-fold more selectivity (S.I. = 56.61) in comparison to the standard drugs pentamidine and sodium stibogluconate, respectively.^15^ In addition, the compound 5 exhibited significant activity IC_50_ < 25 μM against promastigote as well as amastigote.^19^ A triazine dimer 3,3′-(((ethane-1,2-diylbis(azanediyl)) bis(4-(mesityloxy)-1,3,5-triazine-6,2-diyl)) bis(azanediyl))di benzonitrile, 3 (Fig. 2) was observed to display very potent *in vitro* and moderate *in vivo* antitrypanosomal activity.^20^ Interestingly, some natural products Meridianin G, Annomontine (Fig. 2) are potent antileishmanial agents.^21^

Leishmaniasis is an infectious disease caused by protozoa and is the second most critical parasitic disease after malaria. The suggested drugs for leishmaniasis consist of triazole, chalcone, chromone, thiazole, thiosemicarbazone, indole, quinoline *etc.*^22^ Chloroquine and its derivatives, whose nuclear structure is composed of quinoline, have always fascinated chemists and biologists due to the diversity of their chemical and medicinal properties, for example antiparasitic, anticancer, antibacterial, antiviral, antifungal, antioxidant, Anti-asthmatic, antipsychotic, antiglaucoma and cardiovascular agents.^23–25^ The majority of anti-leishmanial agents act by interacting with key regulators including PTR-I, DHFR, LdMetAP1, MAPK, 14 α-demethylase and pteridine reductase-I, *etc.* Also, these tend to induce the production of ROS, which causes damage to parasites. In the present study the *in vitro* and *ex vivo* antileishmanial activity of a chloroquinolin inhibitors, namely 7-chloro-N, *N*-dimethylquinolin-4-amine was evaluated against *L. infantum* and *L. amazonensis*.^26^ The Results showed that the compound was highly effective against *L. infantum* and *L. amazonensis*, presenting a selectivity index of 154.6 and 86.4 against the promastigotes and of 137.6 and 74.3 against the axenic amastigotes respectively. Keeping these things into consideration, we attempted to incorporate different heterocyclic moieties, the synthesis and antileishmanial evaluation of new fifteen 1,2,4 Triazine, pyrimidine hybrid molecules.

## 2. Results and discussions

### 2.1. Chemistry

**Scheme 1:**
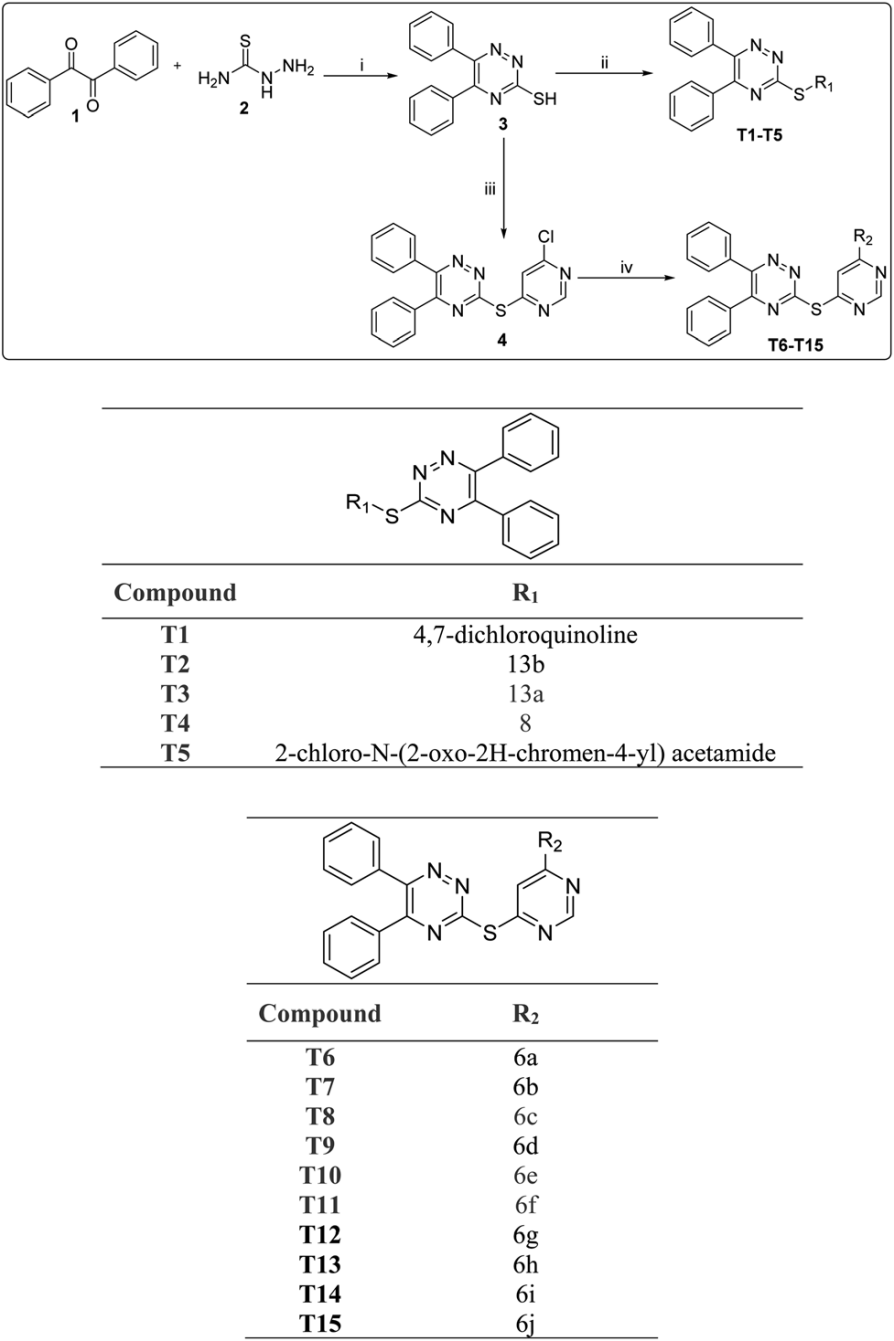
Synthesis of diphenyl triazine based compounds. Reagents and conditions: (i) EtOH, water 1 : 1, 80 °C, reflux, 16 h, (ii) [R_1_H {4,7 dichloroquinoline}] Et_3_N Acetone, reflux, 60 °C, 8 h. (iii) K_2_CO_3_, DMF, reflux, 100 °C, 10 h (iv) [R_2_H{Morpholine}] Et_3_N, DMF, reflux, 100 °C, 10 h.

The target molecules were synthesized *via* multiple steps as represented in Scheme 1. In the first step benzil (1) was made to react with thiosemicarbazide (hydrazinecarbothioamide) 2 in water and ethanol in equal proportions to get 3 according to the reported literature.^27^ The compound 3 was treated with substituted chloro containing complex to get target compounds T1–T5. On the other hand, compounds 3 was treated with 4,6-dichloropyrimidine to get compound 4 in good yields. Further compound 4 was reacted with different nitrogen containing nucleophiles in presence of DMF as a reaction solvent, triethylamine as base to obtain the target molecules T6–T15. Column chromatography technique was used for the purification of all the molecules and ^1^H-NMR, ^13^C-NMR, ESI MS and elemental analysis for characterisation purposes.

### 2.2. Biological evaluation

#### 2.2.1. Anti-leishmanial effect

To identify novel diphenyl-triazine, pyrimidine hybrids, a novel series of 15 compounds were subjected to the screening test against the promastigote stage of *L. donovani*.

Each candidate was evaluated for its *in vitro* activity against reference strain (MHOM/IN/83/AG83) of extracellular promastigotes and intracellular amastigotes of *L. donovani*. The *in vitro* cytotoxicity assay was performed using human macrophage (cell line THP-1). Compounds, which showed good inhibitory activity against the promastigotes, standard antileishmanial, miltefosine and Ampho B were included in the study. We were very pleased to find that among the synthesised analogues T4 and T7 more potent against promastigotes and axenic amastigotes.

#### 2.2.2. *In vitro* antileishmanial activity of diphenyl-triazine

Initially, a series of diphenyl-1,2,4 triazine (T1–T5) were synthesized (Scheme 1) and evaluated for their *in vitro* antileishmanial activity, compounds were added with 100 μM concentration. We observed that compound T4 displayed **99.67%** inhibition of the promastigote stage of the parasites (Table 1). However, compounds T1, T2, T3 and T5 showed <90% inhibition against the parasite, hence we did stop the further testing of compounds T1, T2, T3 and T5. We continued our studies with the compound T4 exhibited the potent activity against promastigote and amastigotes (IC_50_ = 1.074 μM), (IC_50_ = 7.186 μM) respectively. Interestingly, compound T4 behaved better than miltefosine. Miltefosine and Ampho B have been evaluated under the same conditions. Miltefosine displayed significant anti-promastigote activity (IC_50_ = 1.477 μM) (Table 2) and nontoxic toward THP-1 human macrophages cell line. Here, we observed compound T4 bearing 3,4-dimethoxy group on the phenyl ring electron withdrawing groups showed excellent potency against promastigotes and showed 99.67% parasite inhibition in addition was nontoxic for macrophages. Electron withdrawing groups was more effective for the antileishmanial activity.

**Table 1:** Percentage inhibition of *L. donovani* at promastigote stage

Entity	Compound	% Inhibition of *L. donovani* promastigotes
1	T1	66.78
2	T2	71.02
3	T3	83.43
**4**	T4	**99.67**
5	T5	67.93
6	T6	43.86
**7**	T7	**99.89**
8	T8	73.70
9	T9	29.81
10	T10	22.41
11	T11	32.15
12	T12	21.86
13	T13	18.54
14	T14	22.76
15	T15	28.82

**Table 2:** *In vitro* antileishmanial activity of compounds T4, T7 and MTF against *L. donovani* promastigotes and axenic amastigotes

Compound	*L. donovani* promastigotes	*L. donovani* amastigotes
	IC_50_ (μM)	IC50 (μM)
T4	1.074	7.186
T7	1.158	nt
MTF	1.477	
Ampho B	1.038	

#### 2.2.3. *In vitro* antileishmanial activity of diphenyl-triazine – pyrimidine

**Scheme 2:**
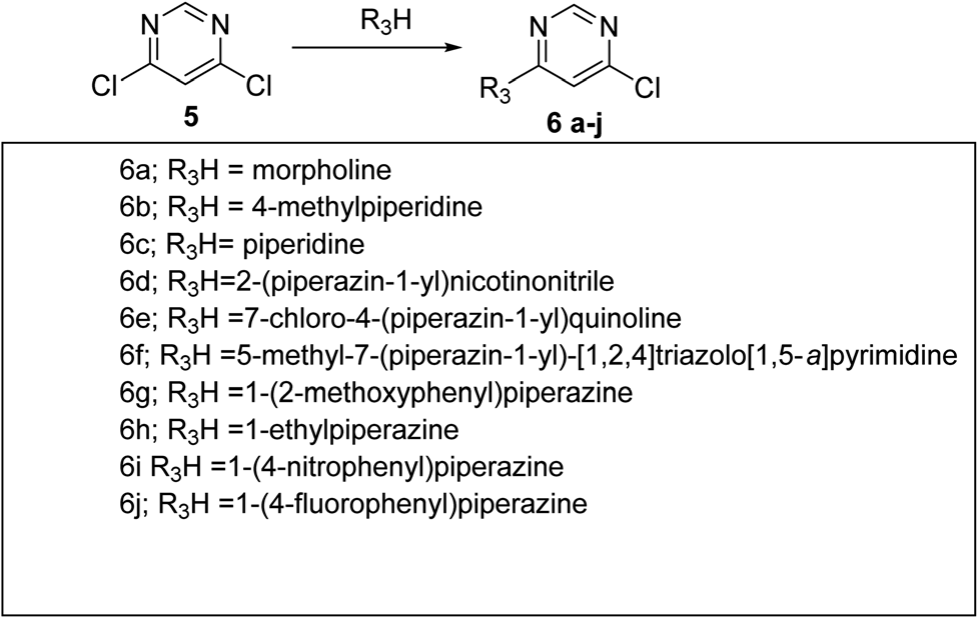
Reagents and conditions: K_2_CO_3_, DMF, reflux 100 °C, overnight.

**Scheme 3:**
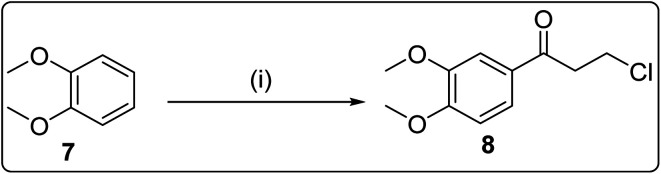
Reagents and conditions: (i) 3-chloropropanoyl chloride, AlCl_3_, CH_2_Cl_2_, 24 h, room temperature.

**Scheme 4:**
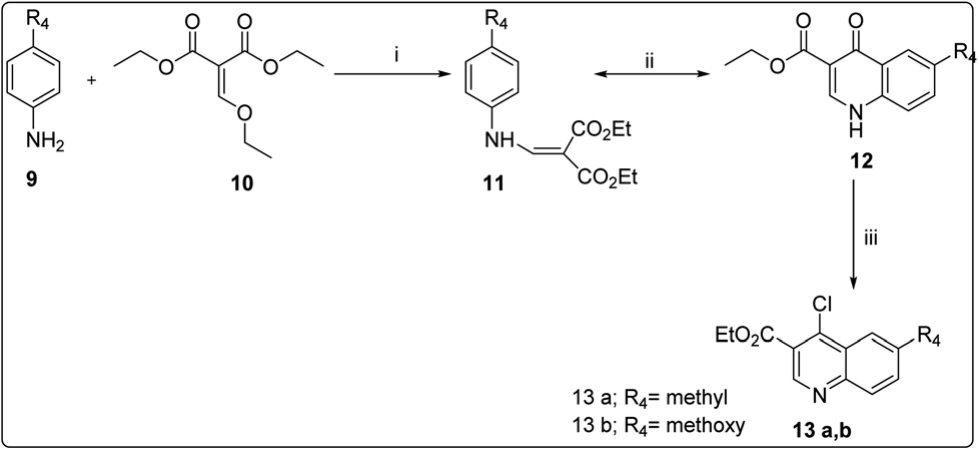
Reagents and conditions. (i) Benzene, 83 °C reflux, 1.5 h (ii) acetic acid, 110 °C reflux 4–5 h; (iii) POCl_3_, 110 °C,18 h.

Previously, we found the role of diphenyl triazine counterpart with the withdrawing group against antileishmanial activity. Furthermore, we did synthesize diphenyl-1,2,4 triazine-pyrimidine (T6–T15) (Scheme 2) analogues and screened for their *in vitro* antileishmanial activity and parasite inhibitions (Table 1). Analogue T7 showed 99.89% inhibition against the promastigote parasite. Compounds T6, T8, T9, T10, T11, T12, T13, T14 and T15 at the100 μM concentration showed a negligible activity against the parasite, hence were not further tested. Compounds T7 exhibited promising anti-promastigote activity having (IC_50_ = 1.158 μM) (Table 2) better than that of miltefosine and compound T7 also showing the good parasite inhibitory activity against the amastigotes (Table 3). Although compound T7 showing more cytotoxicity than T4 on THP-1 cell line (Fig. 5) and was not progressed further. Analogues T7 contained methyl group on the piperidine ring. A progressive enhancement in anti-amastigotes potency was observed in the case of electron donating substituted hydride showing weak cytotoxicity. Furthermore, compound T4 and T7 showed good inhibitory activity against the amastigotes, considering electron withdrawing group important for antileishmanial activity (Schemes 3 and 4).

**Table 3:** Anti-leishmanial effect of the compound T4 and T7 on amastigote parasite^a^

Compounds	Percentage reduction of parasites				
	100 μM	50 μM	25 μM	12.5 μM	6.25 μM
T4	78.0	74	70	68.80	66
T7	72	70.6	66	62	56
Ampho B	84.2	82.5	79.44	76	74
Milte	76	74	73	72	68

a T4 and T7 reduced the infection of macrophages, with highest effect observed at 100 μM. Antiamastigote activity of test molecules against *Leishmania* (*donovani*) decreasing gradually with decrease in concentrations. This effect can be correlated to the anti-promastigote activity of molecules.

#### 2.2.4. Cellular morphological alterations in *L. donovani* promastigotes

**Fig. 3:**
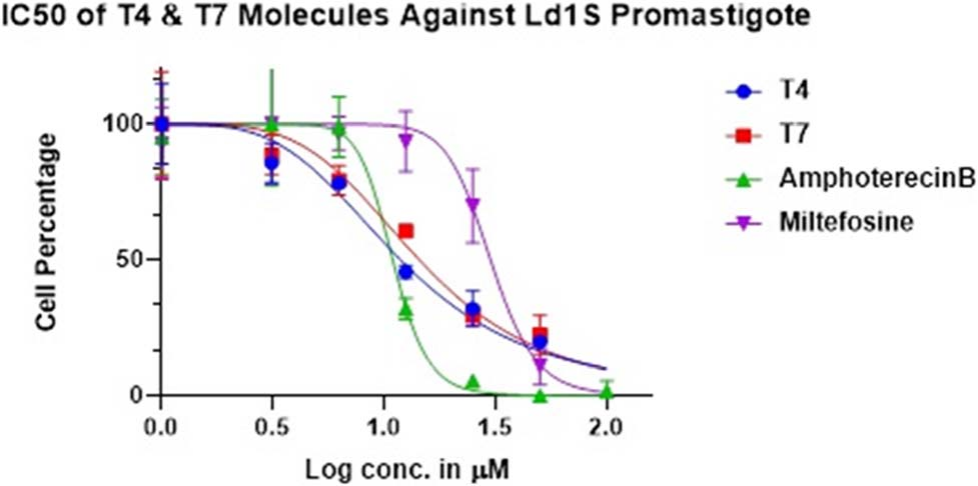
IC_50_ of T4 and T7 molecules against the promastigotes.

**Fig. 4:**
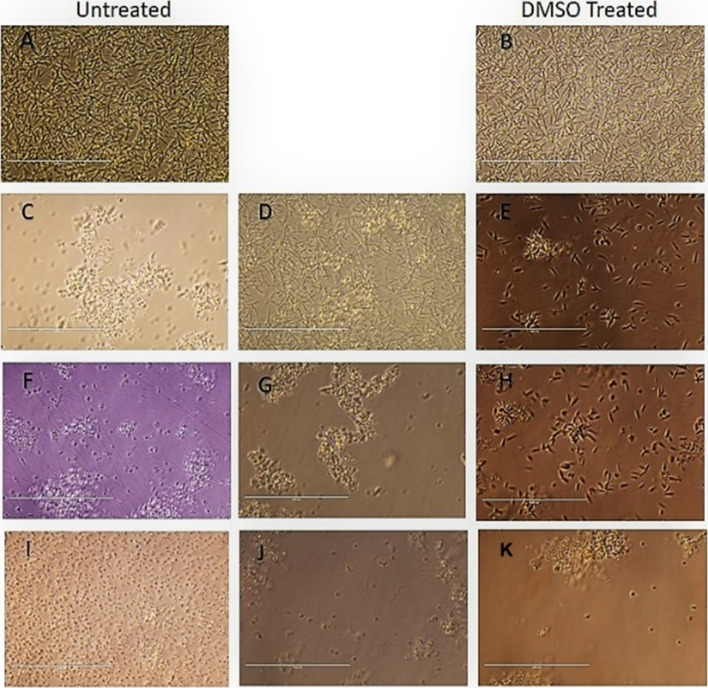
Morphological Analysis of promastigote stage cells treated with compound T4 and MTF, compared to the untreated control (A) and DMSO treated control (B). Exponential-phase promastigotes (1 × 10^6^ cells per mL) were incubated with 25 μm, 50 μm and 100 μm and analysed by light microscopy (40× magnification). (A) Untreated control (B) DMSO Treated cells (C–E) Ampho B, (F–H) T4 and T7 (I–K) at 25, 50, and 100 μM respectively. Scale bar in all is 10 μm.

For the evident of morphological changes, the promastigotes (Fig. 3 (C to L)) were incubated in presence of compound T7, T4 and control Ampho B with the different concentration 25 μM, 50 μM, 100 μM shown in (Fig. 3C to L) were analysed by light microscopy (40×magnification) compared to untreated cells (Fig. 4A). Drastic changes in the cellular morphology of the parasites were evidenced by the loss of flagella at 25, 50 and 100 μM concentration of T7, T4 treatment. Massive cytoplasmic condensation and cell shrinkage were observed in almost all the cells treated with T7 and T4 (Fig. 4J and K). The parasite was found to display ovoidal shape with loss of flagella and apparent reduction in size by the treatment with T7 and T4 compared to the untreated control and MTF treated strains.

#### 2.2.5. Cytotoxicity of the test compound T4, T7, Ampho B and MTF on THP-1 human monocytes

**Fig. 5:**
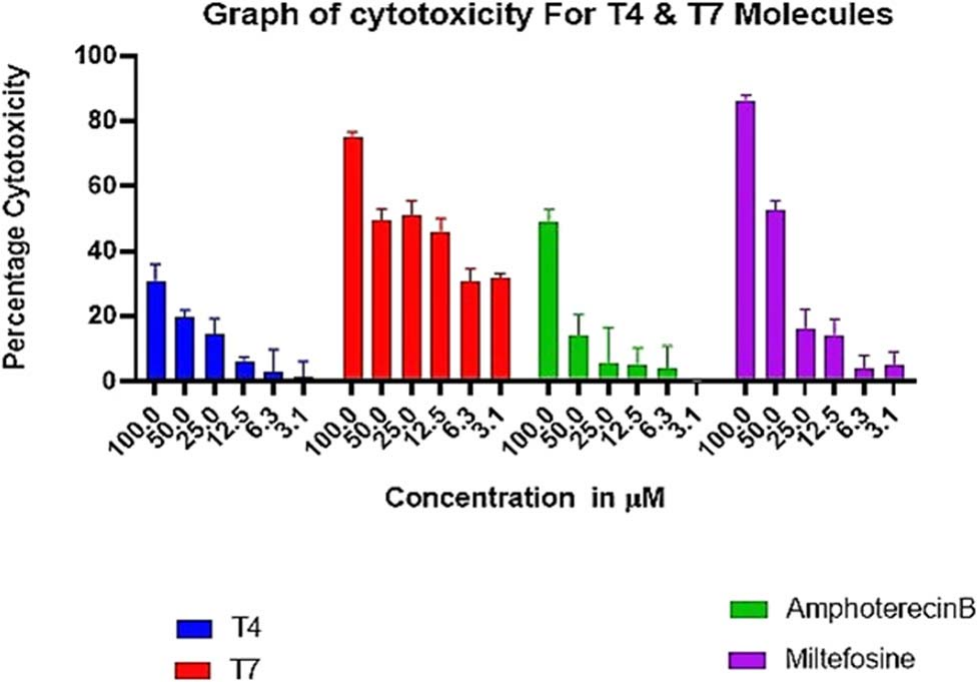
Absence of adverse toxicity of test compounds T4 on THP-1 cells up to 3.125–100 μM in comparison to MTF and Ampho B.

*In vitro* cytotoxicity assays of the selected compound of the series T4 and T7 were carried out with human macrophage cell line THP-1 and were compared with MTF and Ampho B. The toxicity assay revealed that test compounds T4 up to 3.125–100 μM had no adverse effects on the viability and morphology of the macrophages and almost equally safe as Ampho B, while T7 compound showed some cytotoxicity like another standard drug, MTF (Fig. 5). The cell lines were incubated for 48 h at 37 °C in a CO_2_ incubator with increasing concentrations of test compounds and viability was ascertained. Each point or bar corresponds to the mean ± SEM of triplicate samples and is representative of one of three independent experiments.

## 3. Micrographs of *L. donovani* infected macrophages treated with compound T4 and standard drugs Ampho B and miltefosine

**Fig. 6:**
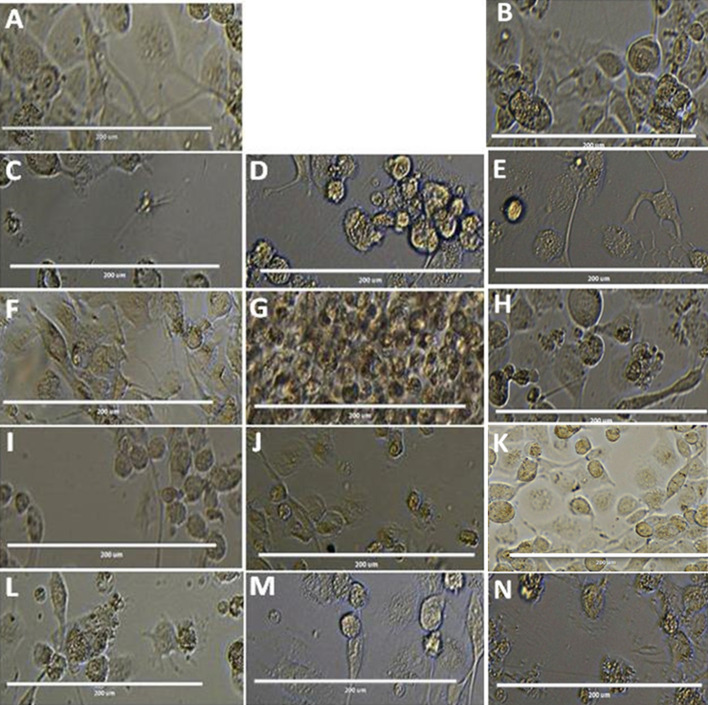
Morphological analysis of compound T4 on THP-1 cells along with controls AmphoB and Milte with different concentrations of Test molecules where (A) untreated control (B) DMSO treated cells (C–H) T4 treated in 100, 50, 25, 12.5, 6.25, 3.125 μM conc. (I–K) Ampho treated cells in 100, 50, 25 μM (L–N) Milte treated cells in 100, 50, 25 μM concentrations respectively. Scale bars in all is 10 μm.

We find that the diphenyl triazine and diphenyl-triazine-pyrimidine showed better activity against promastigotes and amastigotes. Unfortunately, compound T7 displayed cytotoxicity on THP-1 cell line. After that we evaluated the compound T4 on the infected THP-1cells, which was compared with standard antileishmanials, Miltefosine and Ampho B at different concentrations (Fig. 6). Interestingly, we found the compound T4 showed great effect against the promastigotes better than that of well-established antileishmanials. Diphenyl-triazine analogues showing robust potency against the amastigotes of the parasite as well as *L donovani* promastigotes (IC_50_ = 1.074 μM), (IC_50_ = 7.186 μM). Compound T4 can serve as lead for the development of more active antileishmanial agents.

### 3.1. Infection and treatment of *L. donovani*

**Fig. 7:**
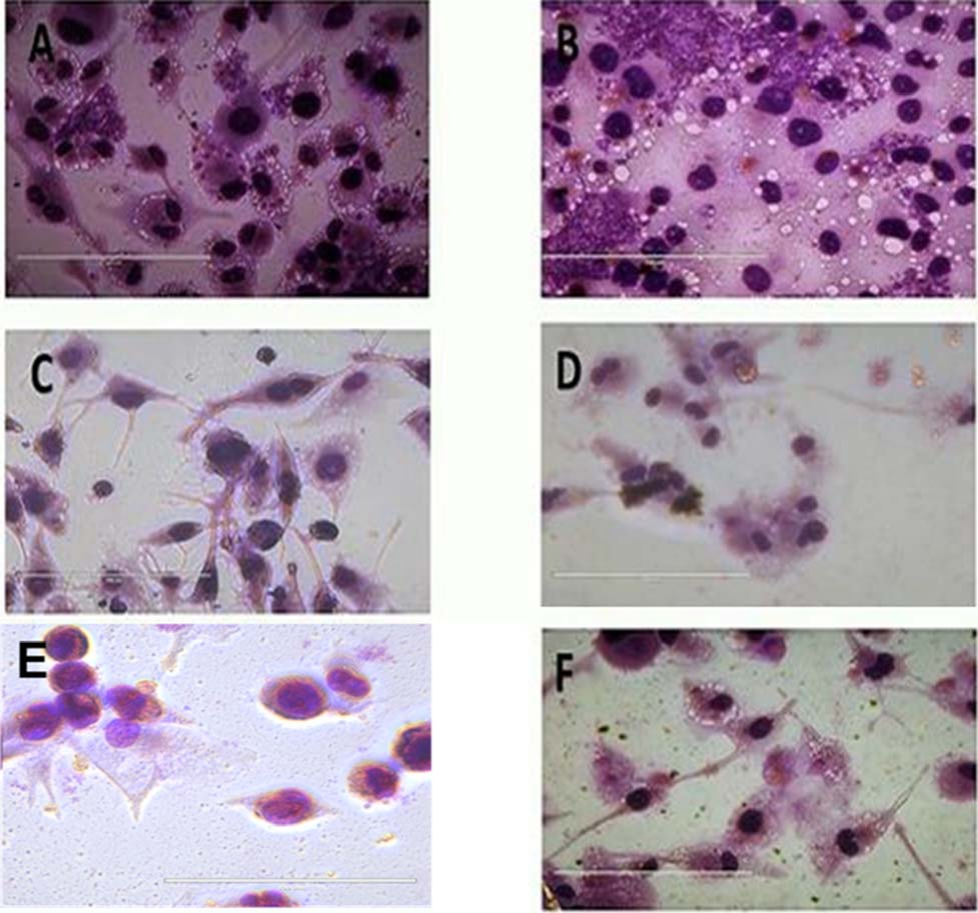
Treatment with internalized parasites. (A) Untreated (B) DMSO, (C) Ampho B, (D) MTF and (E and F) T4 at 100, 50 μM respectively. The scale bar in all is100 μm.

To evaluate the anti-leishmanial effect of the compound T4 and T7 on intracellular *L. donovani* parasites, macrophage was infected with stationary -phase *L. donovani* for 6 h. The cells were then washed with saline to remove any non-internalised parasites. To assess the effect of compounds T4 and T7 on intracellular parasites at early stages of infection, macrophages were subsequently treated with T4 and T7 at concentrations of 100, 50, 25, 12.5, or 6.25 μM (refer to Table 3 and Fig. 7) for 6, 24, or 48 hours. Observation of treated macrophages revealed that both T4 and T7 were capable of inhibiting the growth of Leishmania *L. donovani* species, indicating the potential of these compounds as chemotherapeutic agents for the treatment of *L. donovani* leishmaniasis.

## 4. Conclusions

A series of diphenyl-triazine core moieties was synthesized and evaluated for their antileishmanial efficacy. The compounds were synthesized using organic chemistry methods and their structures were evaluated using NMR and mass spectrometry techniques. These diphenyl-triazine hybrids were screened against the promastigote stage of *L. donovani*. Compounds T4 and T7 demonstrated significant inhibition of the promastigote stage of the parasites with >90% inhibition and IC_50_ values of 1.074 and 1.158 μM, respectively. Conversely, compounds T12, T13, T14 and T15 exhibited minimal activity against the parasite. Compound T4, which incorporates (3,4-dimethoxyphenyl) propanone attached to the diphenyl-triazine core moiety, also effectively inhibited the parasite at intracellular amastigotes, with an IC_50_ of 5.18 μM. Assessment of the toxicity profile of T4 on the human macrophage cell line THP-1 indicated its non-toxic nature compared to the marketed MTF. In conclusion, compounds such as T4 show promise as lead candidates for the development of more potent antileishmanial agents.

## 5. Experimental section

### 5.1. Chemistry

The chemicals, reagents and solvents used during the synthesis and characterization of the compounds were from Sigma-Aldrich. ^1^H NMR and ^13^CNMR spectra were observed on Bruker 126, 75 and 500, 399, 300 MHz spectrophotometer using deuterated solvents (CDCl_3_, *δ* 7.26; DMSO-d6 *δ* 2.54) and multiplicities of NMR signals were designated as s (singlet), d (doublet), dd (double doublet), t (triplet), q (quartet), m (multiplet, for unresolved lines). Chemical shifts were showed in *δ* (ppm) and tetramethylsilane was used as the internal standard. Mass spectra were recorded on UPLC XEVO G2-XS QTOF Spectrometer (HRMS) instrument. Thin layer chromatography was performed with Merck silica gel (60–120 and F254) aluminum-coated sheets of 0.25 mm thickness. Spots on these were observed in short (254 nm) with ultraviolet light and long (365 nm) wavelengths. Elemental analyses were obtained on Elementar Vario analyser. Elemental analyses of the compounds were found to be within ±0.4% of the theoretical values. The purity of tested compounds was >95%.^1^

#### 5.1.1. General procedure for the synthesis of compound 3

Thiosemicarbazide (0.65 g, 7.1 mol) was dissolved in 30 mL water at room temperature. K_2_CO_3_ (2 g, 14.4 mol) was added to the stirring solution of thiosemicarbazide. The reaction mixture was allowed for stirring at room temperature for 1 hour. Now ethanolic solution of benzil (1.5 g, 7.1 mol) was added. The reaction mixture was refluxed at 80 °C for 16 h. After the completion of the reaction, the reaction mixture was acidified to pH 3 with acetic acid to obtain a yellow-coloured precipitate. The precipitate obtained was filtered and dried and was used for forward reaction steps without any purification.

#### 5.1.2. General procedure for the synthesis of compounds T1–T5

5,6-Diphenyl-1,2,4-triazine-3-thiol 3 (10 mmol) was dissolved in 20 mL acetone at room temperature. Et_3_N (30 mmol) was added to the stirring solution, after 30 minutes and different chloro-substituted aromatic compounds (R_1_) (10 mmol) were added. The reaction mixture was refluxed at 60 °C for 8 h. After the completion of the reaction brought the temperature to cool, acetone was removed by rota vapour, dried the crude mixture, dissolved the ethyl acetate 300 mL and washed with water (3 × 300 mL) followed by brine separated the organic layer dried with Na_2_SO_4_ obtain to crude product (T1–T5) purified by column chromatographic using silica (200–400 mesh) gel (EtOAc/hexane).

##### 5.1.2.1. 7-Chloro-4-((5,6-diphenyl-1,2,4-triazin-3-yl)thio)quinoline (T1)

Yellow, m.p. 250–252 °C, 60% yield was obtained after column chromatography. HRMS (ESI+): calcd for [C24H15ClN4S + H]^+^: *m*/*z* = 426.07 found: 427.0834. ^1^H NMR (400 MHz) *δ* 8.96 (d, *J* = 4.4 Hz 1H), 8.29 (d, *J* = 9.0 Hz, 1H), 8.19 (d, *J* = 2.1 Hz, 1H), 7.89 (d, *J* = 4.4 Hz, 1H), 7.54 (dd, *J* = 9.0, 2.1 Hz, 1H), 7.48 (d, *J* = 8.0 Hz, 2H), 7.41–7.34 (m, 3H), 7.32 (dd, *J* = 7.8, 1.4 Hz, 3H), 7.25–7.22 (m, 2H). ^13^C NMR (126 MHz, CDCl_3_) *δ* 169.01, 155.89, 154.81, 150.80, 134.78, 134.52, 131.31, 129.80, 129.74, 129.31, 128.96, 128.82, 128.68, 128.56, 128.38, 127.91, 126.89. Anal. calcd for C_24_H_15_ClN_4_S C, 67.52; H, 3.54; N, 13.12 found C, 66.51; H, 4.01; N, 13.02.

##### 5.1.2.2. Ethyl 4-((5,6-diphenyl-1,2,4-triazin-3-yl)thio)-6-methoxyquinoline-3-carboxylate (T2)

Light yellow, m.p. 200–202 °C, 55% yield was obtained after column chromatography. HRMS (ESI^+^): calcd for [C_28_H_22_N_4_O_3_S + H]^+^: *m*/*z* = 494.14 found: 495.1500 ^1^H NMR (500 MHz, CDCl_3_) *δ* 9.23 (s, 1H), 8.14 (d, *J* = 10.0 Hz, 1H), 7.90 (d, *J* = 2.6 Hz, 1H), 7.50 (d, *J* = 10.0 Hz, 3H), 7.44–7.32 (m, 5H), 7.26 (dd, *J* = 15.4, 7.7 Hz, 2H), 4.32 (q, *J* = 7.1 Hz, 2H), 3.91 (s, 3H), 1.27 (t, *J* = 5.0 Hz, 3H). ^13^C NMR (126 MHz, CDCl_3_) *δ* 169.53, 165.79, 159.41, 155.57, 154.38, 147.19, 145.68, 135.85, 134.93, 134.60, 131.58, 131.16, 130.73, 129.89, 129.80, 129.67, 129.62, 129.29, 128.65, 128.48, 124.54, 104.06, 61.94, 55.79, 14.04. Anal. calcd for C_28_H_22_N_4_O_3_S C, 68.00; H, 4.48; N, 11.33; found C, 67.50; H, 4.07; N, 11.03.

##### 5.1.2.3. Ethyl 4-((5,6-diphenyl-1,2,4-triazin-3-yl)thio)-6-methylquinoline-3-carboxylate (T3)

White, m.p. 210–212 °C, 62% yield was obtained after column chromatography. HRMS (ESI+): calcd for [C_28_H_22_N_4_O_2_S + H]^+^: *m*/*z* = 478.15 found: 479.1561. ^1^H NMR (500 MHz, CDCl_3_) *δ* 9.32 (s, 1H), 8.44 (s, 1H), 8.17 (d, *J* = 8.5 Hz, 1H), 7.71 (d, *J* = 8.6 Hz, 1H), 7.50 (d, *J* = 8.2 Hz, 1H), 7.43–7.33 (m, 1H), 7.27 (dd, *J* = 14.2, 6.3 Hz, 1H), 4.32 (q, *J* = 7.1 Hz, 1H), 2.58 (s, 1H), 1.27 (t, *J* = 7.1 Hz, 1H). ^13^C NMR (126 MHz, CDCl_3_) *δ* 169.55, 165.58, 155.57, 154.40, 148.60, 138.95, 134.96, 134.63, 134.09, 131.13, 129.80, 129.65, 129.51, 129.30, 129.26, 128.64, 128.47, 125.43, 61.99, 22.02, 14.03. Anal. calcd for C_28_H_22_N_4_O_2_S C, 70.27; H, 4.63; N, 11.71; found C, 71.03; H, 4.23; N, 12.31.

##### 5.1.2.4. 1-(3,4-Dimethoxyphenyl)-3-((5,6-diphenyl-1,2,4-triazin-3-yl)thio)propan-1-one (T4)

Pale yellow, m.p. 210–212 °C, 71% yield was obtained after column chromatography. HRMS (ESI+): calcd for [C_26_H_23_N_3_O_3_S] *m*/*z* = 457.15 found: 480.1518 and 496.1119 [C_26_H_23_N_3_O_3_S + Na]^+^: [C_26_H_23_N_3_O_3_S + K]^+^. ^1^H NMR (500 MHz, CDCl_3_) *δ* 7.63 (dd, *J* = 8.3, 1.4 Hz, 2H), 7.58 (d, *J* = 10.5 Hz, 1H), 7.55 (d, *J* = 3.7 Hz, 1H), 7.53 (s, 1H), 7.46–7.41 (m, 2H), 7.39 (d, *J* = 7.7 Hz, 2H), 7.34 (t, *J* = 7.7 Hz, 2H), 7.29 (s, 1H), 6.87 (d, *J* = 10.0 Hz, 1H), 3.96 (s, 6H), 3.76 (t, *J* = 6.9 Hz, 3H), 3.62 (t, *J* = 6.9 Hz, 3H). ^13^C NMR (126 MHz, CDCl_3_) *δ* 196.68, 170.63, 155.71, 153.92, 153.48, 149.09, 135.26, 135.14, 130.93, 129.86, 129.82, 129.47, 129.31, 128.62, 128.52, 122.86, 110.15, 110.07, 56.09, 56.04, 38.14, 25.54. Anal. calcd for C_26_H_23_N_3_O_3_S C, 68.25; H, 5.07; N, 9.18; found C, 67.65; H, 5.02; N, 10.00.

##### 5.1.2.5. 2-((5,6-Diphenyl-1,2,4-triazin-3-yl)thio)-*N*-(2-oxo-2*H*-chromen-4-yl)acetamide (T5)

Light yellow, m.p. 200–202 °C, 52% yield was obtained after column chromatography. HRMS (ESI^+^): calcd for [C_26_H_18_N_4_O_3_S + H]^+^: *m*/*z* = 466.11 found: 467.1179 and [C_26_H_18_N_4_O_3_S + Na]^+^: 489.1132. ^1^H NMR (500 MHz, CDCl_3_) *δ* 9.61 (s, 1H), 8.70 (s, 1H), 7.57 (t, *J* = 6.5 Hz, 4H), 7.51 (d, *J* = 10.0 Hz, 1H), 7.47–7.42 (m, 3H), 7.39 (d, *J* = 5.0 Hz, 1H), 7.36 (s, 1H), 7.34 (s, 1H), 7.32 (s, 1H), 7.30 (s, 1H), 7.29 (s, 1H), 4.21 (s, 2H). ^13^C NMR (126 MHz, CDCl_3_) *δ* 168.83, 167.79, 158.36, 156.30, 154.84, 150.11, 134.87, 134.73, 131.24, 129.91, 129.75, 129.70, 129.47, 128.65, 128.57, 127.84, 125.09, 124.18, 123.88, 119.73, 116.35, 35.41. Anal. calcd for C_26_H_18_N_4_O_3_S C, 66.94; H, 3.89; N, 12.01; found C, 67.01; H, 3.59; N, 11.89.

#### 5.1.3. General procedure for the synthesis of compound 4

Compound 3 (5 mmol) was dissolved in DMF at the room temperature. K_2_CO_3_ (10 mmol) was added to the stirring solution after 20 minutes, then 4,6 dichloropyrimidine (5 mmol) was added to the reaction mixture and the reaction mixture was refluxed at 100 °C for 10 h. The reaction mixture was cooled to room temperature, dissolved with ethyl acetate and washed with water (3 × 300 mL) followed by washing of the organic layer with brine. The organic layer was dried over Na_2_SO_4_. The crude mixture was purified by column chromatography using 20% EtOAc/hexane to obtain pure yellow powdered solid product 4.

#### 5.1.4. General procedure for the synthesis of compounds T6–T15

3-((6-Chloropyrimidin-4-yl) thio)-5,6-diphenyl-1,2,4-triazine 4 (1 mmol) was dissolved in 30 mL DMF. Et_3_N (3 mmol) was added to the stirring solution after few minutes, then different amines R_2_ (1 mmol) were added and the reaction mixture was refluxed at 100 °C for 10 h. After the completion of the reaction, the reaction mixture was cooled to room temperature and diluted with ethyl acetate and water (thrice). The organic part was washed with brine. The crude mixture was purified with column chromatography using 50% ethyl acetate and hexane to get the target compounds T6–T15 in good yields.

##### 5.1.4.1. 4-(6-((5,6-Diphenyl-1,2,4-triazin-3-yl)thio)pyrimidin-4-yl)morpholine (T6)

Yellow, m.p. 198–200 °C, 50% yield was obtained after column chromatography. ESI *m*/*z* [M + H]^+^ calcd 428.14 found 428.9. ^1^H NMR (399 MHz, cdcl3) *δ* 8.55 (s, 1H), 7.54 (d, *J* = 0.7 Hz, 1H), 7.52 (d, *J* = 1.2 Hz, 2H), 7.43 (d, *J* = 3.2 Hz, 1H), 7.41 (d, *J* = 1.5 Hz, 1H), 7.39 (s, 2H), 7.37 (d, *J* = 1.6 Hz, 1H), 7.34 (s, 1H), 7.32 (s, 1H), 7.26 (s, 1H), 3.752 (t, *J* = 4.8 Hz,4H), 3.66 (t, *J* = 5.2 Hz,4H). ^13^C NMR (126 MHz, cdcl_3_) *δ* 170.67, 163.97, 159.94, 157.83, 156.98, 133.19, 131.85, 131.79, 131.37, 130.72, 130.57, 106.68, 68.43, 46.18. Anal. calcd for C_23_H_20_N_6_OS C, 64.47; H, 4.70; N, 19.61; found C, 64.42; H, 5.20; N, 19.42.

##### 5.1.4.2 3-((6-(4-Methylpiperidin-1-yl)pyrimidin-4-yl)thio)-5,6-diphenyl-1,2,4-triazine (T7)

Chock white, m.p. 250–252 °C, 53% yield was obtained after column chromatography. ESI *m*/*z* [M + H]^+^ calcd 440.18 found 442.0. ^1^H NMR (399 MHz, cdcl_3_) *δ* 8.52 (s, 1H), 7.53 (s, 1H), 7.51 (d, *J* = 0.8 Hz, 2H), 7.41 (d, *J* = 2.1 Hz, 1H), 7.40 (d, *J* = 1.2 Hz, 1H), 7.37 (s, 1H), 7.35 (d, *J* = 0.8 Hz, 2H), 7.32 (s, 1H), 7.30 (s, 1H), 7.28 (s, 1H), 3.67 (s, 4H), 2.56 (t, *J* = 5.0 Hz, 1H), 2.45 (t, *J* = 5.2 Hz,4H), 2.32 (s, 3H).^13^C NMR (100 MHz, cdcl3) *δ* 168.75, 161.69, 161.63, 157.96, 155.75, 154.88, 134.90, 134.82, 131.12, 130.16, 129.83, 129.72, 129.34, 129.07, 128.67, 128.53, 128.24, 128.20, 104.85, 54.47, 46.02, 43.78, 43.41. Anal. calcd for C_25_H_24_N_6_S C, 68.16; H, 5.49; N, 19.08; found C, 67.76; H, 5.14; N, 18.78.

##### 5.1.4.3 5,6-Diphenyl-3-((6-(piperidin-1-yl)pyrimidin-4-yl)thio)-1,2,4-triazine (T8)

Pale yellow, m.p. 240–242 °C, 63% yield was obtained after column chromatography. ESI *m*/*z* [M + H]^+^ calcd 426.16 found 427.15. Anal. calcd for C_24_H_22_N_6_S C, 67.58; H, 5.20; N, 19.70; found C, 67.58; H, 5.20; N, 19.70; ^1^H NMR (500 MHz, cdcl_3_) *δ* 8.53 (s, 1H), 8.38 (s, 1H), 7.54 (dd, *J* = 6.9, 1.3 Hz, 1H), 7.43 (d, *J* = 8.0 Hz, 1H), 7.39 (s, 2H), 7.38 (s, 1H), 7.32 (t, *J* = 7.6 Hz, 1H), 7.30 (s, 1H),7.29 (s,1H), 6.78 (s, 2H), 3.61 (d, *J* = 25.6 Hz, 4H), 1.69 (dd, *J* = 11.2, 5.1 Hz, 2H), 1.60 (dd, *J* = 10.2, 4.3 Hz, 4H). ^13^C NMR (126 MHz, cdcl_3_) *δ* 166.34, 161.51, 158.11, 157.50, 131.06, 129.86, 129.68, 129.38, 128.66, 128.52, 105.11, 96.20, 45.15, 25.43, 24.45.

##### 5.1.4.4 2-(4-(6-((5,6-Diphenyl-1,2,4-triazin-3-yl)thio)pyrimidin-4-yl)piperazin-1-yl)nicotinonitrile (T9)

Light yellow, m.p. 250–252 °C, 59% yield was obtained after column chromatography. ESI *m*/*z* [M + H]^+^ calcd 529.18 found 530.12 ^1^H NMR (500 MHz, cdcl_3_) *δ* 8.58 (s, 1H), 8.40–8.36 (m, 1H), 7.82 (d, *J* = 7.6 Hz, 1H), 7.56 (s, 2H), 7.55 (s, 2H), 7.45 (s, 2H), 7.43 (d, *J* = 8.3 Hz, 1H), 7.39 (d, *J* = 6.8 Hz, 2H), 7.35 (d, *J* = 7.3 Hz, 2H), 6.83 (dd, *J* = 7.5, 4.9 Hz, 1H), 3.83 (d, *J* = 8.0 Hz, 8H). ^13^C NMR (126 MHz, cdcl_3_) *δ* 168.54, 162.19, 161.52, 157.88, 155.92, 155.07, 154.12, 138.90, 134.82, 134.76, 131.24, 129.87, 129.35, 128.75, 128.59, 126.03, 112.46, 104.65, 46.17, 43.07. Anal. calcd for C_29_H_23_N_9_S C, 65.77; H, 4.38; N, 23.80; found C, 66.01; H, 4.53; N, 23.43.

##### 5.1.4.5 7-Chloro-4-(4-(6-((5,6-diphenyl-1,2,4-triazin-3-yl)thio)pyrimidin-4-yl)piperazin-1-yl) quinoline (T10)

Yellow, m.p. 260–262 °C, 57% yield was obtained after column chromatography. ESI *m*/*z* [M + H]^+^ calcd 588.16 found 589.15. ^1^H NMR (399 MHz, cdcl_3_) *δ* 8.75 (d, *J* = 5.0 Hz, 1H), 8.60 (s, 1H), 8.10 (d, *J* = 2.0 Hz, 1H), 7.98 (d, *J* = 9.0 Hz, 1H), 7.56 (s, 1H), 7.55–7.54 (m, 2H), 7.53 (d, *J* = 2.0 Hz, 2H), 7.48 (dd, *J* = 9.0, 2.1 Hz, 1H), 7.43 (d, *J* = 2.7 Hz, 1H), 7.42–7.41 (m, 1H), 7.40 (s, 1H), 7.38 (s, 1H), 7.33 (d, *J* = 7.8 Hz, 1H), 7.26 (s, 1H), 6.86 (d, *J* = 5.0 Hz, 1H), 3.96 (s, 4H), 3.44 (t, *J* = 5.2 Hz, 4H). ^13^C NMR (126 MHz, cdcl_3_) *δ* 168.96, 162.38, 158.01, 156.51, 155.90, 155.05, 151.71, 134.83, 134.78, 131.27, 131.22, 129.87, 129.84, 129.35, 128.91, 128.88, 128.75, 128.58, 128.54, 126.68, 126.66, 124.80, 109.99, 109.13, 104.83, 51.76, 43.96. Anal. calcd for C_32_H_25_ClN_8_S C, 65.24; H, 4.28; Cl, 6.02; N, 19.02; found C, 65.32; H, 4.45; Cl, 5.88; N, 19.12.

##### 5.1.4.6 7-(4-(6-((5,6-Diphenyl-1,2,4-triazin-3-yl)thio)pyrimidin-4-yl)piperazin-1-yl)-5-methyl-[1,2,4]triazolo[1,5-*a*]pyrimidine (T11)

Yellow cotton, m.p. 280–285 °C, 68% yield was obtained after column chromatography. ESI *m*/*z* [M + H]^+^ calcd 559.20 found 559.8. ^1^H NMR (300 MHz, CDCl_3_) *δ* 8.58 (s, 1H), 8.32 (s, 1H), 8.01 (s, 1H), 7.56–7.54 (m, 2H), 7.54–7.50 (m, 3H), 7.41 (d, *J* = 3.3 Hz, 2H), 7.38–7.28 (m, 3H), 6.19 (s, 1H), 2.96 (s, 4H), 2.88 (s, 4H), 2.60 (s, 3H). ^13^C NMR (75 MHz, CDCl_3_) *δ* 168.46, 165.09, 162.52, 162.45, 161.64, 157.91, 157.11, 155.93, 155.05, 154.27, 149.77, 134.72, 131.26, 129.83, 129.33, 128.73, 128.60, 104.69, 94.57, 47.29, 43.20, 36.50. Anal. calcd for C_29_H_25_N_11_S C, 62.24; H, 4.50; N, 27.53; found C, 61.89; H, 3.95; N, 27.33.

##### 5.1.4.7. 103-((6-(4-(2-methoxyphenyl)piperazin-1-yl)pyrimidin-4-yl)thio)-5,6-diphenyl-1,2,4-triazine (T12)

White m.p. 250–255 °C, 64% yield was obtained after column chromatography. ESI *m*/*z* [M + H]^+^ calcd 533.20 found 534.20. ^1^H NMR (399 MHz, cdcl_3_) *δ* 8.56 (d, *J* = 0.9 Hz, 1H), 7.53 (dd, *J* = 7.6, 0.8 Hz, 3H), 7.42 (s, 2H), 7.40 (s, 1H), 7.38 (s, 1H), 7.37 (d, *J* = 1.6 Hz, 1H), 7.35 (t, *J* = 1.6 Hz, 1H), 7.32 (d, *J* = 7.8 Hz, 1H), 7.26 (s, 1H), 6.92 (s, 1H), 6.90 (s, 1H), 6.86 (s, 1H), 6.84 (s, 1H), 3.81 (s, 3H), 3.76 (d, *J* = 3.9 Hz, 4H), 3.16 (t, *J* = 5.5 4H). ^13^C NMR (126 MHz, cdcl_3_) *δ* 163.61, 159.86, 157.66, 156.78, 156.25, 147.04, 136.73, 136.67, 133.01, 131.71, 131.62, 131.22, 130.55, 130.41, 120.71, 120.65, 116.40, 106.76, 57.41, 52.49, 45.90. Anal. calcd for C_30_H_27_N_7_OS C, 67.52; H, 5.10; N, 18.37; found C, 67.32; H, 4.89; N, 18.17.

##### 5.1.4.8. 113-((6-(4-Ethylpiperazin-1-yl)pyrimidin-4-yl) thio)-5,6-diphenyl-1,2,4-triazine (T13)

Light yellow, m.p. 280–285 °C, 55% yield was obtained after column chromatography. ESI *m*/*z* [M + H]^+^ calcd 455.19 found 456.0. ^1^H NMR (399 MHz, cdcl_3_) *δ* 7.54 (s, 1H), 7.52 (s, 1H), 7.48 (d, *J* = 1.4 Hz, 2H), 7.43 (dd, *J* = 5.0, 2.8 Hz, 2H), 7.38 (dd, *J* = 7.4, 1.9 Hz, 2H), 7.32–7.29 (m, 4H), 7.26 (s, 1H), 4.14 (s, 4H), 2.70 (s, 4H), 2.60 (q, *J* = 7.1 Hz, 2H), 1.21 (t, *J* = 7.2 Hz, 3H). ^13^C NMR (126 MHz, cdcl_3_) *δ* 138.19, 132.19, 131.81, 131.61, 131.60, 131.31, 131.04, 130.65, 130.51, 130.23, 130.19, 54.43, 54.27, 45.03, 13.40. Anal. calcd for C_25_H_25_N_7_S C, 65.91; H, 5.53; N, 21.52; found C, 66.11; H, 5.44; N, 21.34.

##### 5.1.4.9. 123-((6-(4-(4-Nitrophenyl)piperazin-1-yl)pyrimidin-4-yl)thio)-5,6-diphenyl-1,2,4-triazine (T14)

Yellow, m.p. 260–265 °C, 73% yield was obtained after column chromatography. ESI *m*/*z* [M + H]^+^ calcd 548.17 found 548.9 ^1^H NMR (300 MHz, CDCl_3_) *δ* 8.58 (s, 1H), 8.15 (d, *J* = 9.3 Hz, 2H), 7.56–7.54 (m, 2H), 7.53 (dd, *J* = 2.9, 1.4 Hz, 2H), 7.53 (s, 2H), 7.41 (d, *J* = 4.8 Hz, 2H), 7.34 (dd, *J* = 15.4, 7.6 Hz, 3H), 6.80 (d, *J* = 9.3 Hz, 2H), 3.88 (t, *J* = 4.8 Hz, 4H), 3.75 (t, *J* = 5.7 Hz, 4H). ^13^C NMR (75 MHz, CDCl_3_) *δ* 168.56, 162.27, 161.52, 157.95, 155.92, 155.05, 138.83, 134.76, 131.25, 129.87, 129.35, 128.76, 128.60, 126.03, 112.43, 104.65, 46.14, 43.03. Anal. calcd for C_29_H_24_N_8_O_2_S C, 63.49; H, 4.41; N, 20.42; found C, 62.89; H, 4.10; N, 21.01.

##### 5.1.4.10. 3-((6-(4-(4-Fluorophenyl)piperazin-1-yl)pyrimidin-4-yl)thio)-5,6-diphenyl-1,2,4-triazine (T15)

Light yellow, m.p. 270–272 °C, 57% yield was obtained after column chromatography. ESI *m*/*z* [M + H]^+^ calcd 521.18 found 522.0. ^1^H NMR (300 MHz, CDCl_3_) *δ* 8.56 (s, 1H), 7.54 (d, *J* = 7.2 Hz, 4H), 7.43 (s, 2H), 7.41 (d, *J* = 3.3 Hz, 2H), 7.33 (dd, *J* = 15.6, 7.8 Hz, 3H), 6.99 (t, *J* = 8.4 Hz, 2H), 6.90 (dd, *J* = 7.8, 3.6 Hz, 2H), 3.82 (s, 4H), 3.15 (t, *J* = 4.5 Hz, 4H). ^13^C NMR (75 MHz, CDCl_3_) *δ* 168.73, 161.94, 161.74, 158.04, 156.03, 155.84, 154.97, 147.55, 134.89, 131.19, 129.88, 129.38, 128.73, 128.59, 118.55, 118.44, 115.89, 115.60, 104.90, 50.13, 43.94. Anal. calcd for C_29_H_24_FN_7_S C, 66.78; H, 4.64; N, 18.80; found C, 65.99; H, 5.21; N, 19.01.

#### 5.1.5. General procedure for the synthesis of compounds 6a–6j

A mixture of 4,6-dichloropyrimidine 5 (10 mmol), different amines (R_3_) (10 mmol) and K_2_CO_3_ (13 mmol) in anhydrous dimethylformamide (DMF) in round bottom flask was refluxed at 100 °C overnight. The reaction completion as well as formation of desired product was preliminarily confirmed by TLC. The reaction mixture was cooled to room temperature and diluted by ethyl acetate (100 mL) and the organic layer was washed with water and then with brine solution. The organic layer was dried over sodium sulphate, concentrated and purified by column chromatography using 15–20% EtOAc/hexane to obtain compounds 6a–6j.

#### 5.1.6. General procedure for the synthesis of compound 8

A mixture of aluminium chloride (5.3 g, 39.7 mol) and 25 mL DCM was allowed for stirring at room temperature. 3-Chloropropionylchloride (5.5 g, 43.3 mol) dissolved in 20 mL DCM was added dropwise to the stirring solution of AlCl_3_. After half an hour 1,2-dimethoxybenzene (5.0 g, 36.1 mol) was added to the reaction mixture and the mixture was allowed for stirring for 24 h at room temperature. On the completion of the reaction, the reaction mixture was poured into ice-cold water and the organic part was extracted with DCM (50 mL × 3). The organic layer was washed with sodium bicarbonate and brine solutions and was dried over Na_2_SO_4_. The crude product 8 was purified by column chromatography using ethyl acetate hexane 25 : 75.

#### 5.1.7. General procedure for the synthesis of compounds 13a and 13b

A solution of 3 (85 mmol) in POCl_3_ (1.34 mol) could reflux at 110 °C for 18 h. On the completion of the reaction, the reaction mixture was cooled and concentrated under vacuum. The resulting brown oil was obtained in CH_2_Cl_2_ (500 mL) and was washed with water (250 mL × 3). The organic extract received was dried through Na_2_SO_4_ and concentrated in vacuum to give a brown oil. The crude product (5a–5d) was chromatographed on silica gel eluting with 15% EtOAc/hexane.^28^

## 6. Biological assays

### 6.1. Leishmanial parasite culture and maintenance

Promastigotes were routinely cultured at 26 °C in medium M199 supplemented with 10% heat inactivated fetal bovine serum (FBS, Gibco Laboratories, Mumbai, India), penicillin 100 (IU mL^−1^), streptomycin (100 μg mL^−1^). Log phase promastigotes were sub-cultured every 72–96 h, the inoculum being 2 × 10^6^ cells per mL.^29^

### 6.2. Cell line culture

THP-1 human monocytic cells were grown at 37 °C in medium RPMI-1640 (pH 7.4, Sigma-Aldrich, St. Louis, MO, USA) supplemented with 10% heat-inactivated FBS for 48 to 72 h in a humidified atmosphere of 5% CO_2_ and sub-cultured in fresh RPMI-1640 medium at an average density 2 × 10^5^ cells per mL (Sharma *et al.* 2023).^30^

### 6.3. Anti-leishmanial activity

The triazin hybrids series was evaluated for their antileishmanial ability against *L. donovani* promastigotes *in vitro*. Promastigotes of *L. donovani* (1× 10^6^ cells per mL) in M199 medium were incubated at 26 °C for 72 h with each compound at a concentration of 100 μM. Miltefosine (MTF) was used as a reference drug, 0.2% DMSO as solvent control. Parasites with media alone were taken as control. Parasite viability was achieved by MTT assay after 72 h (Sharma *et al.* 2023).^30^

### 6.4. Cytotoxicity assay

Human macrophage THP-1 cell line of 0.5 × 10^6^ cells per mL seeded in RPMI-1640 medium (Sigma-Aldrich, USA) supplemented with 10% FBS (GIBCO) in presence of 5% CO_2_ at 37 °C in triplicate in 96 well culture plate. The cells were treated with two-fold serially diluted concentrations (100–3.125 μM) of the candidate molecules for 72 h. The IC_50_ was calculated using MTT assay (Sharma *et al.* 2023).^30^

### 6.5. Dose-dependent anti-promastigote activity and determination of IC_50_

Promastigotes at density of 1 × 10^6^ cells per mL were incubated in the absence and or presence of most active compounds T1–T15 of the series at serial six-fold dilutions starting at 3.125 μM for 3 days at 26 °C. MTF was used as a standard antileishmanial drug control. The cell viability was evaluated (by MTT Assay) and the mean percentage viability was calculated as follows: Mean cell number of treated parasites/mean cell number of untreated parasites × 100. The 50 and 90% inhibitory concentration (IC_50_) *i.e.*, the concentration of drugs that decreased the cell growth by 50 and 90% respectively, was determined by graphical extrapolation after plotting the graph of percentage viability *vs.* concentration of the drug.^31^

### 6.6. Determination of promastigote cellular morphology

Variations in the cellular morphology of *Leishmania* parasites due to the treatment with compounds T4 and T7 was detected microscopically. Briefly, the promastigotes (1× 10^6^ cells per mL) were incubated in the absence or presence of test compounds and MTF and AmphoB for 72 h with different concentrations and observed under 40× objective of a phase-contrast microscope. At least 20 microscopic fields were observed for each sample. Data were recorded by using NIS-Elements imaging software.^32^

The 50% inhibitory concentration of the highly potent compounds T4 and T7 is represented in (Table 2). The test compounds showed a similar trend like that of MTF and Ampho B in dose dependent parasite killing at promastigote stage with IC_50_ values at 1.074 ± 0.09403 μM and 1.158 ± 0.92568 μM respectively. Parasite viability was not affected by DMSO (0.2%, data not shown) used as solvent control.^33^

### 6.7. Anti-amastigote assay

THP-1 cells (5 × 10^5^ cells per mL) were cultivated in RPMI-1640 media (Roswell Park Memorial Institute 1640) (Sigma-Aldrich, USA), supplemented with 10% fetal calf serum and maintained at 37 °C with 5% CO_2_ using phorbol ester as the inducer. These cells were added on circular coverslips (1 × 10^6^ cells per mL). Promastigotes of *L. donovani* were introduced at 1 : 20 ratio. Treatment of the samples was carried out at doses of 100, 50, 25, 12.5, 6.25 and 3.125 μM/72 h. After being taken off, the coverslips were stained with Giemsa. The rate of macrophage infection was then ascertained.

## Author contributions

A. S. and A. R. were involved in the design and synthesis of the compounds under the guidance of NH. A. S. analyzed all chemical data. M. A. B., S. J., and A. K. performed *in vitro* biological studies under the supervision A. S., S. S. and M. A. B. also analyzed all biological data.

## Conflicts of interest

There are no conflicts of interest to declare for any of the authors.

## Supplementary Material

RA-014-D4RA01904K-s001
