# Supplementary material for: Robust leishmanicidal upshot of some new diphenyl triazine-based molecules
Source: RSC Adv. 2024 Jul 17;14(31):22587–97. doi: 10.1039/d4ra01904k (PMC11253633; doi:10.1039/d4ra01904k)
Supplement: RA-014-D4RA01904K-s001 [file RA-014-D4RA01904K-s001.pdf]

Electronic Supplementary Material (ESI) for RSC Advances.
This journal is © The Royal Society of Chemistry 2024

Supporting information

Robust leishmanicidal upshot of some new diphenyl triazine-based molecules

Anju Singh[1], Mirza Adil Beg[2], Samra Jamal[3], Arif Khan[4], Abdur Rahman[1], A. SelvaPandiyan[2], Syed Shafi[4], Nasimul Hoda[1]*

[1]Drug Design and Synthesis Lab., Department of Chemistry, Jamia Millia Islamia, Jamia Nagar, New Delhi-110025, India

[2]JH- Department of molecular medicine, Jamia Hamdard New Delhi-110062, India

[3] Department of Biotechnology, Jamia Hamdard New Delhi-110062, India

[4]Department of Chemistry, SCLS, Jamia Hamdard New Delhi-110062, India

*Corresponding authors

Nasimul Hoda
Professor
Department of Chemistry,
Jamia Millia Islamia,
Jamia Nagar, New Delhi-110025, India
Tel.: +0091-9910200655;
fax: +0091-11-26985507;
e-mail: nhoda@jmi.ac.in

1. General procedure for the synthesis of compounds 6a–6j (Scheme S1)

A mixture of 4,6-dichloropyrimidine **5** (10 mmol), different amines **(R_3)** (10 mmol) and K_2CO_3 (13 mmol) in anhydrous dimethylformamide (DMF) in round bottom flask was refluxed at 100 °C overnight. The reaction completion as well as formation of desired product was preliminarily confirmed by TLC. The reaction mixture was cooled to room temperature and diluted by ethyl acetate (100 mL) and the organic layer was washed with water and then with brine solution. The organic layer was dried over sodium sulphate, concentrated and purified by column chromatography using 15–20% EtOAc/Hexane to obtain compounds **6a–6j.**

Scheme S1.

Scheme 2.

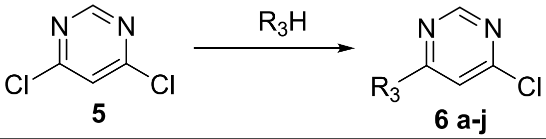

5 **6 a-j**

6a; R_3H = morpholine
6b; R_3H = 4-methylpiperidine
6c; R_3H= piperidine
6d; R_3H=2-(piperazin-1-yl)nicotinonitrile
6e; R_3H =7-chloro-4-(piperazin-1-yl)quinoline
6f; R_3H =5-methyl-7-(piperazin-1-yl)-[1,2,4]triazolo[1,5-*a*]pyrimidine
6g; R_3H =1-(2-methoxyphenyl)piperazine
6h; R_3H =1-ethylpiperazine
6i R_3H =1-(4-nitrophenyl)piperazine
6j; R_3H =1-(4-fluorophenyl)piperazine

Reagents and conditions: K_2CO_3, DMF, reflux 100 °C, overnight.

2. General procedure for the synthesis of compound 8 (Scheme S2)

A mixture of aluminium chloride (5.3 g, 39.7 mol) and 25 mL DCM was allowed for stirring at room temperature. 3-chloropropionylchloride (5.5 g, 43.3 mol) dissolved in 20 mL DCM was added dropwise to the stirring solution of $AlCl_3$. After half an hour 1,2-dimethoxybenzene (5.0 g, 36.1 mol) was added to the reaction mixture and the mixture was allowed for stirring for 24 h at room temperature. On the completion of the reaction, the reaction mixture was poured into ice-cold water and the organic part was extracted with DCM (50 mL x 3). The organic layer was washed with sodium bicarbonate and brine solutions and was dried over Na_2SO_4. The crude product **8** was purified by column chromatography using ethyl acetate hexane 25:75.

Scheme S2.

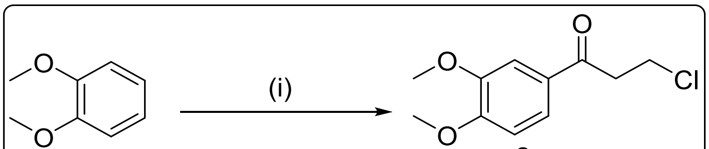

7 **8**

Reagents and conditions:(i)3-chloropropanoyl chloride, $AlCl_3$, CH_2Cl_2, 24h, room temperature

3. General procedure for the synthesis of compounds 13a and 13b (Scheme S3)

A solution of **3** (85 mmol) in $POCl_3$ (1.34 mole) could reflux for 18 h. On the completion of the reaction, the reaction mixture was cooled and concentrated under vacuum. The resulting brown oil was obtained in CH_2Cl_2 (500 mL) and was washed with water (250 mL x 3). The organic extract received was dried through Na_2SO_4 and concentrated in vacuum to give a brown oil. The crude product **(5a–5d)** was chromatographed on silica gel eluting with 15% EtOAc/ Hexane.[1]

Scheme S3.

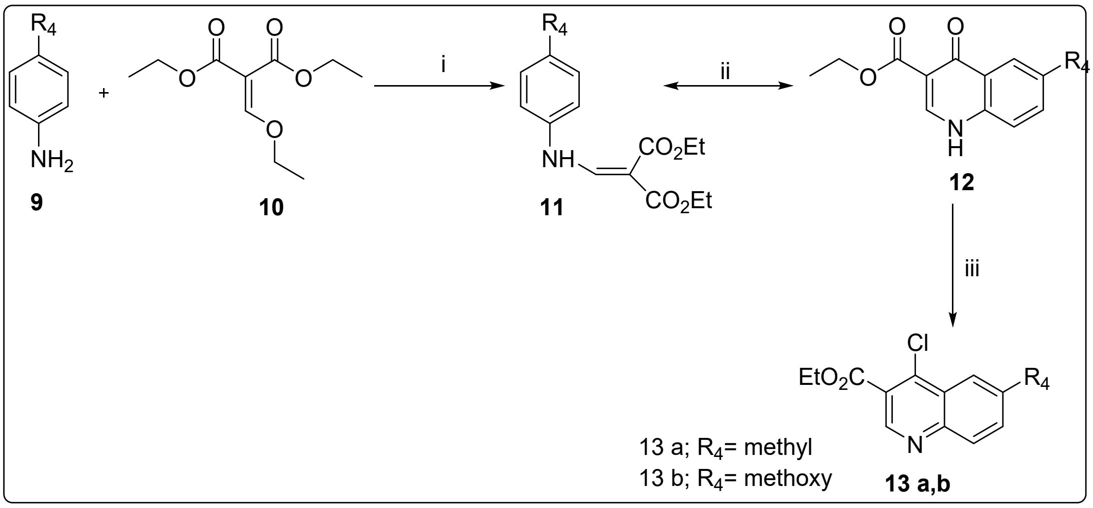

Reagents and conditions. (i) Benzene, 83 °C reflux ,1.5h (ii) Acetic acid, 110 C reflux 4-5h; (iii) $POCl_3$,110 C,18h.

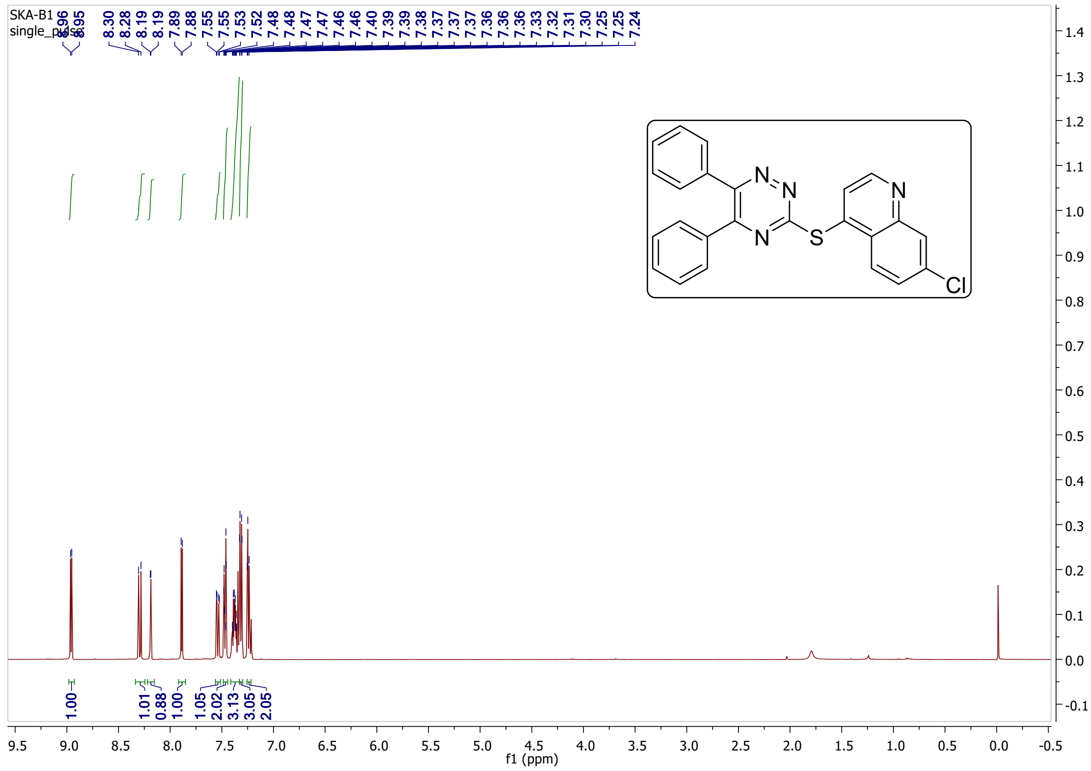

Spe

ctral data of the synthesized compounds

Figure S1: ¹H NMR spectrum of Molecule (T1)

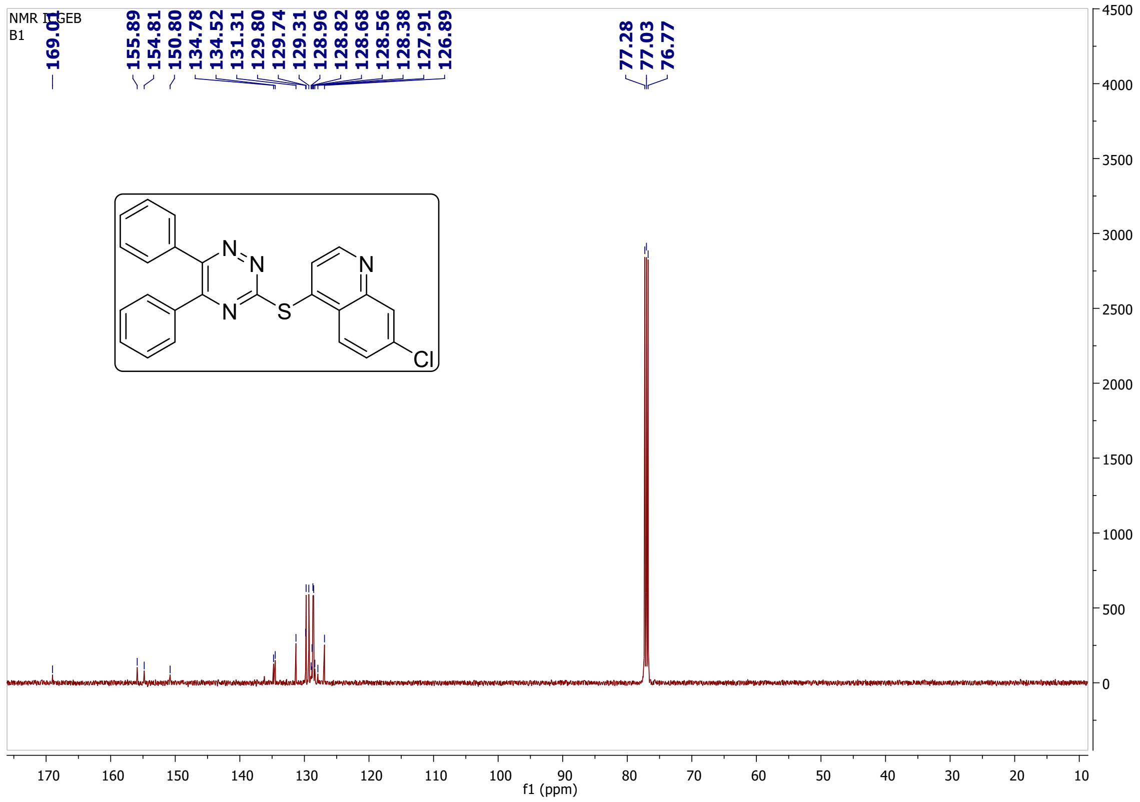

Generic Display Report

Analysis Info

Analysis Name	D:\Data\yang jh\20190210-B1.d	Acquisition Date	2/10/2019 1:27:24 PM
Method	20170112_pos_50-1000.m	Operator	Bruker07
Sample Name	B1-1	Instrument	micrOTOF-Q
Comment			

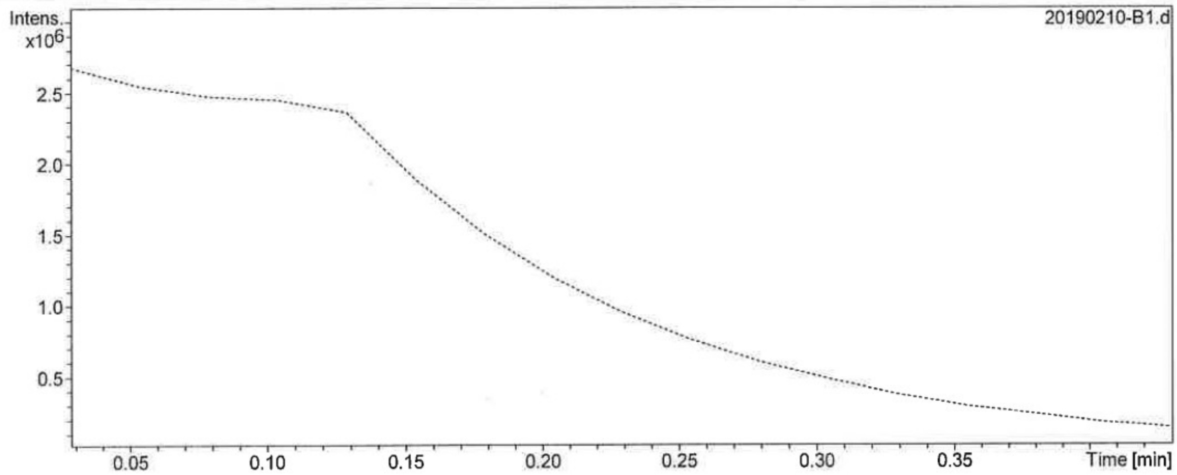

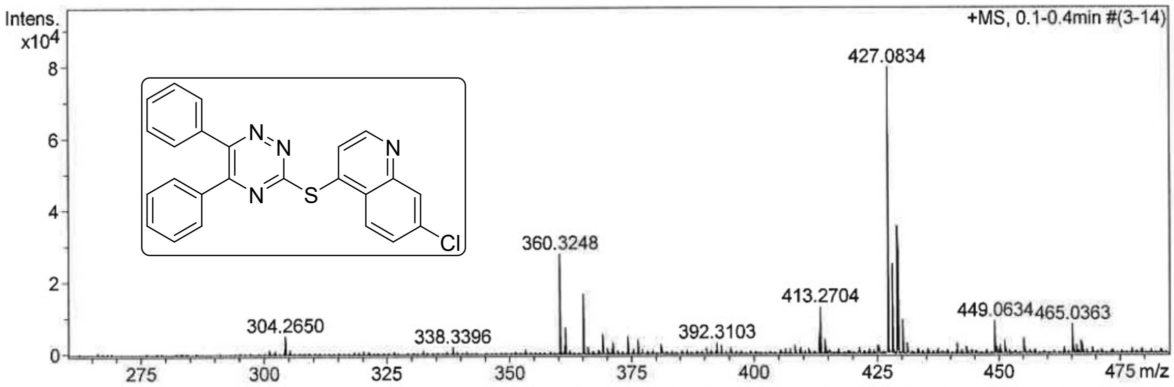

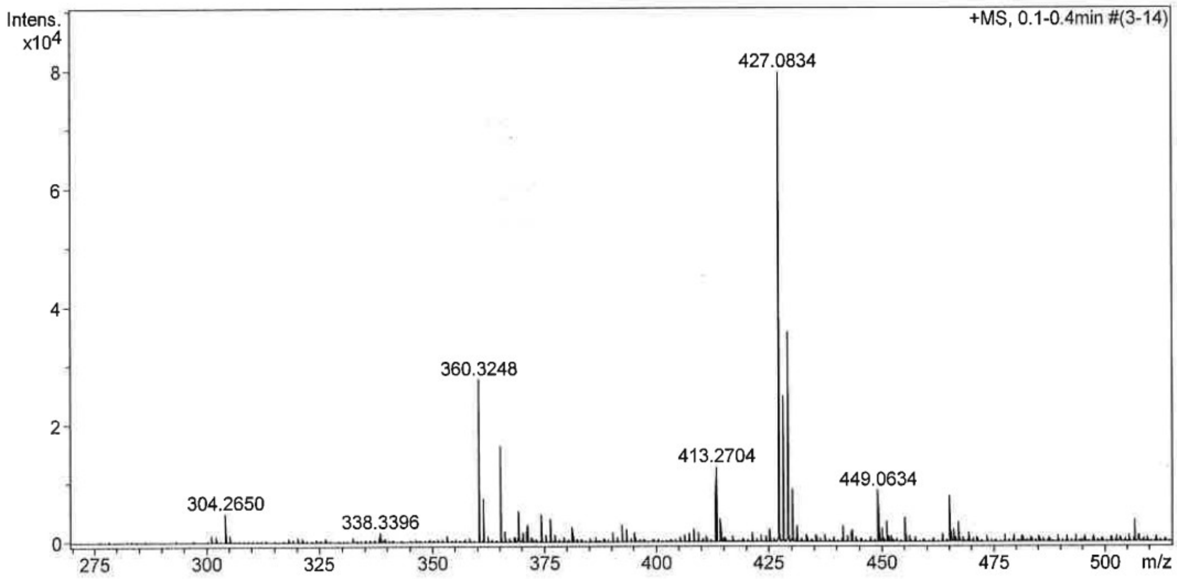

Figure S3: Mass spectrum of Molecule (T1)

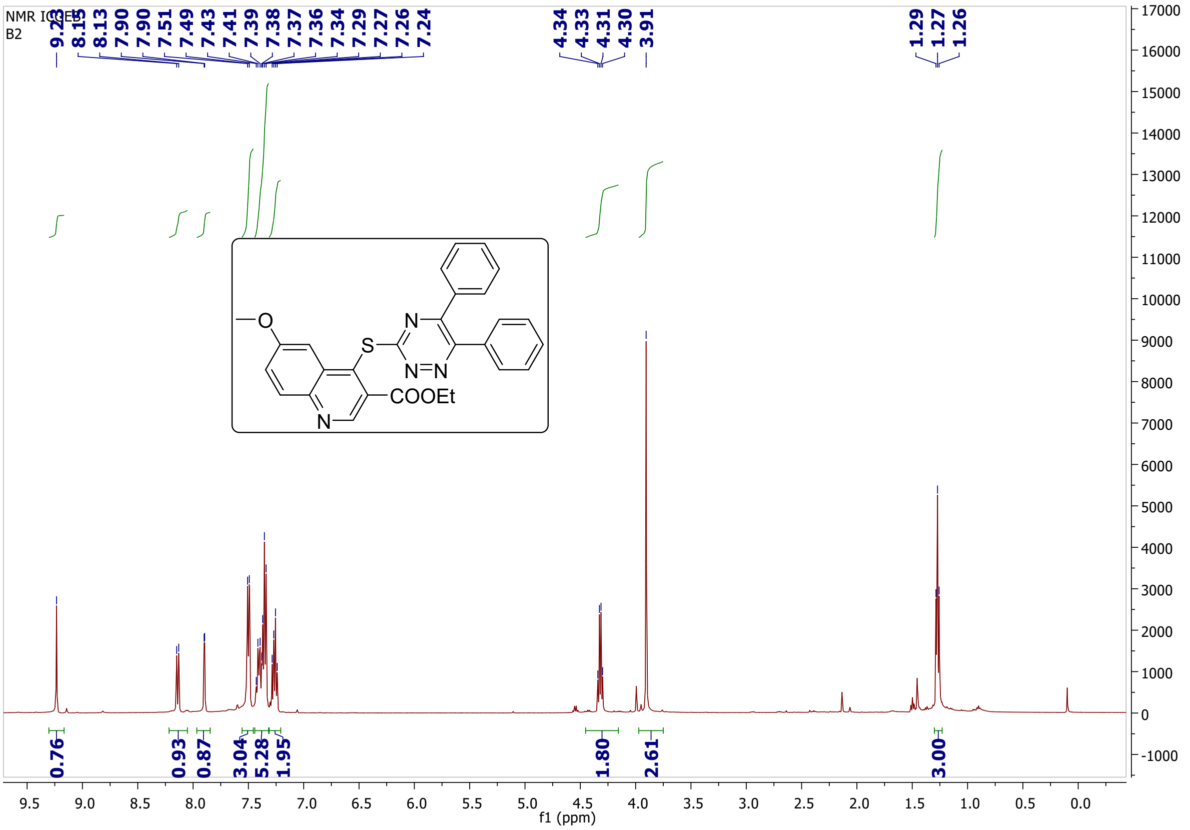

Figure S4: ^{1}H NMR spectrum of Molecule (T2)

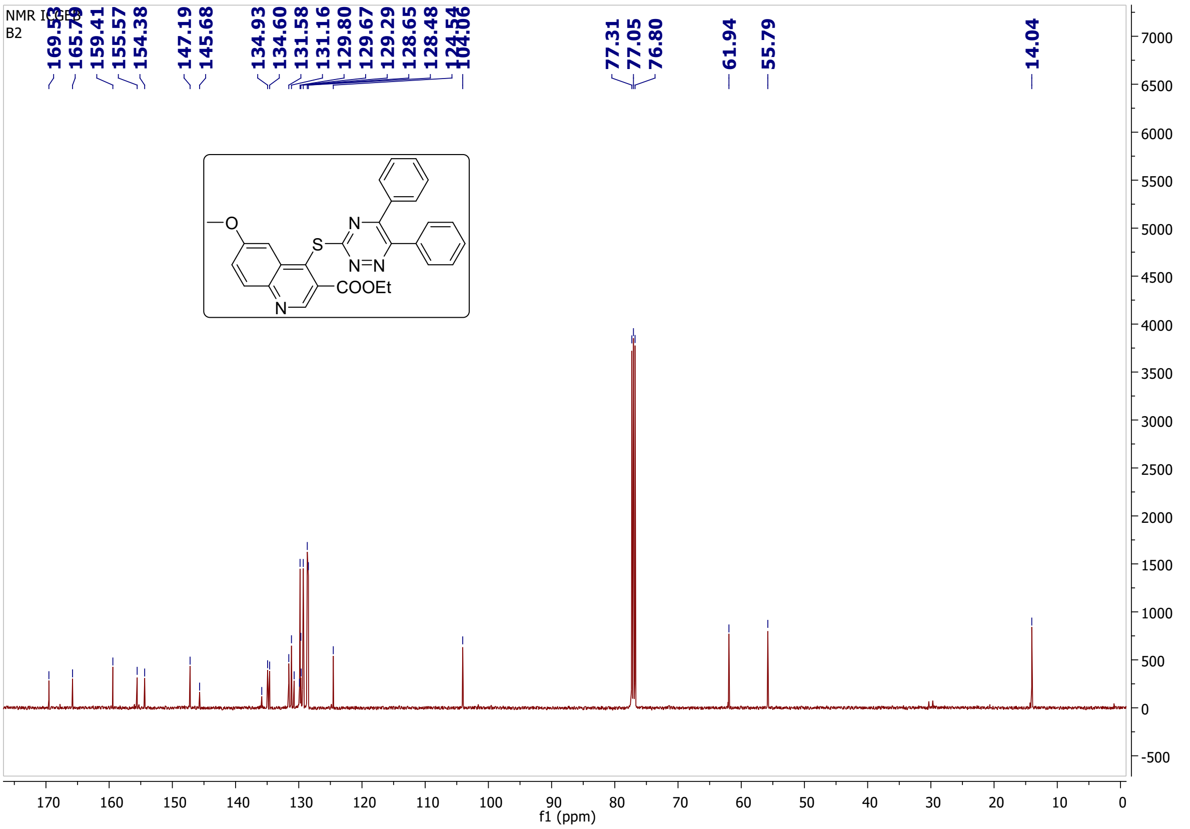

Figure S5: ^{13}C NMR spectrum of Molecule (T2)

Generic Display Report

Analysis Info

Analysis Name	D:\Data\yang jh\20190210-B2.d	Acquisition Date	2/10/2019 1:41:00 PM
Method	20170112_pos_50-1000.m	Operator	Bruker07
Sample Name	B2-1	Instrument	micrOTOF-Q
Comment			

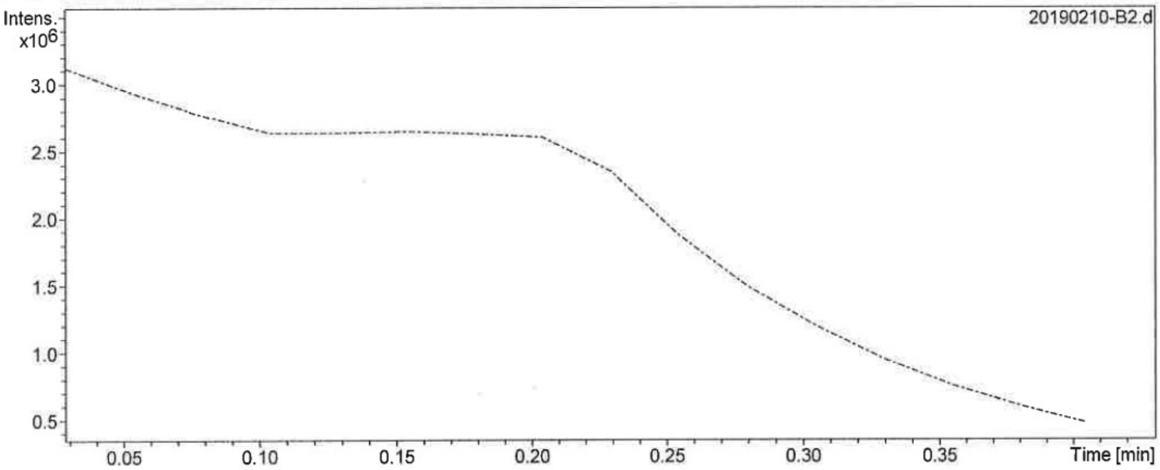

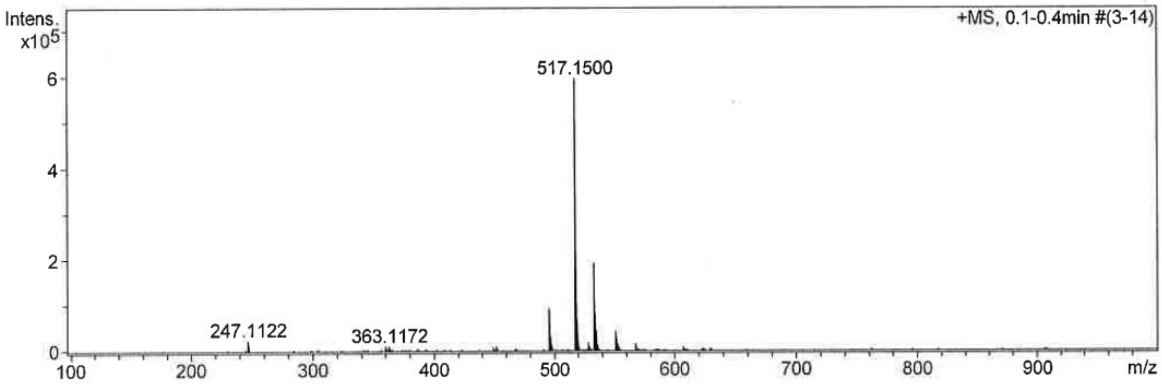

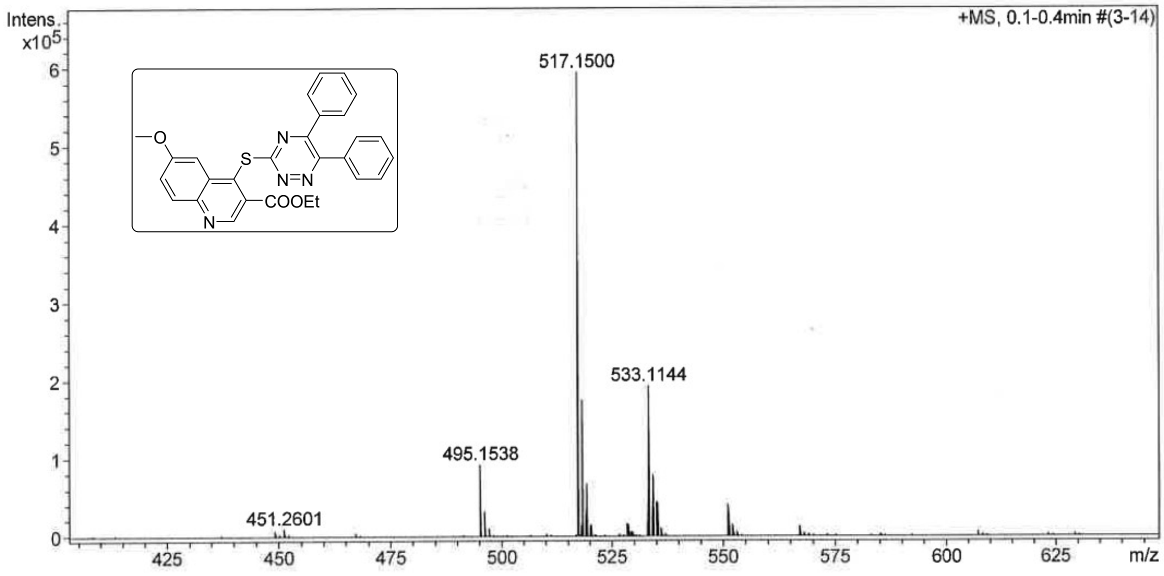

Figure S6: Mass spectrum of Molecule (T2)

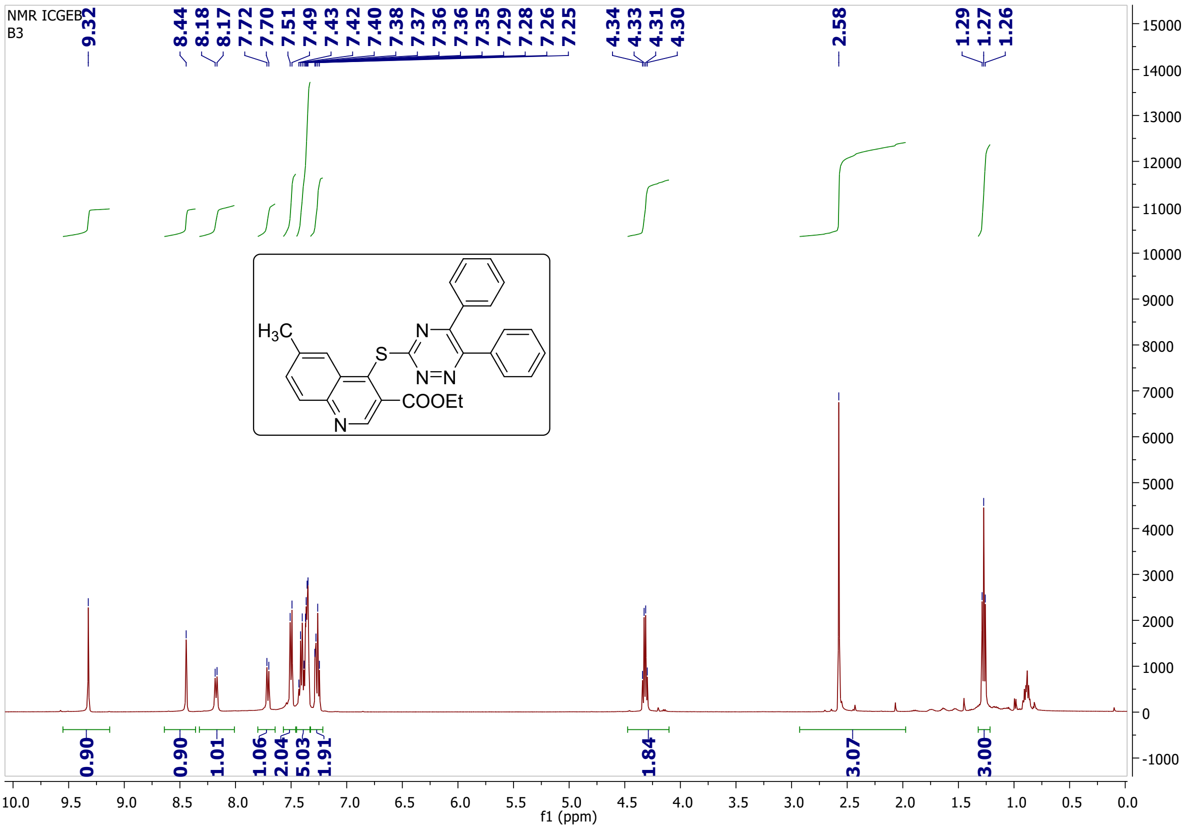

Figure S7: ^{1}H NMR spectrum of Molecule (T3)

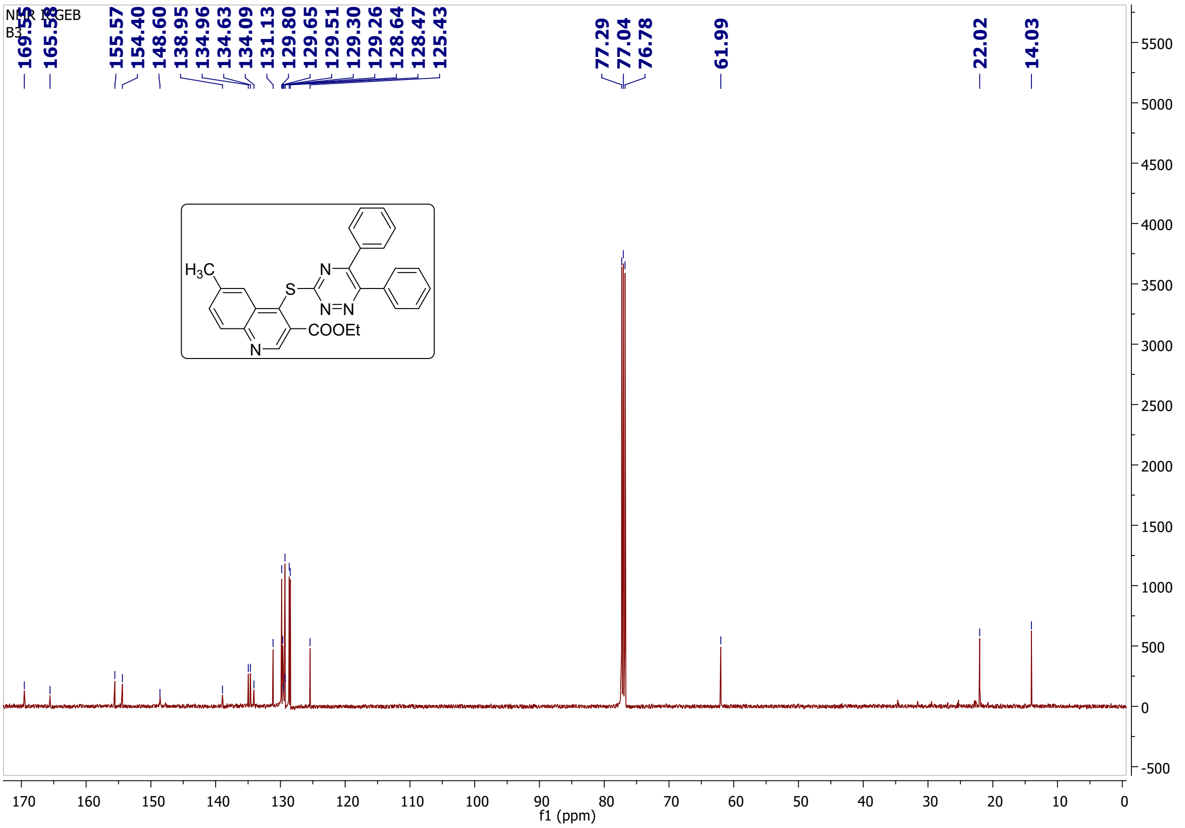

Figure S8: ^{13}C NMR spectrum of Molecule (T3)

Generic Display Report

Analysis Info

Analysis Name	D:\Data\yang jh\20190210-B3.d	Acquisition Date	2/10/2019 1:57:22 PM
Method	20170112_pos_50-1000.m	Operator	Bruker07
Sample Name	B3-1	Instrument	micrOTOF-Q
Comment			

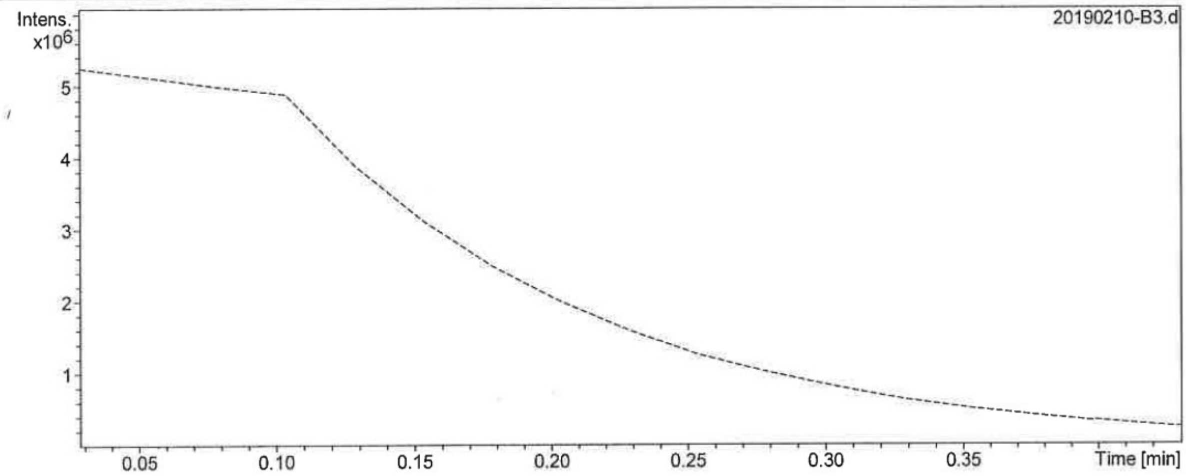

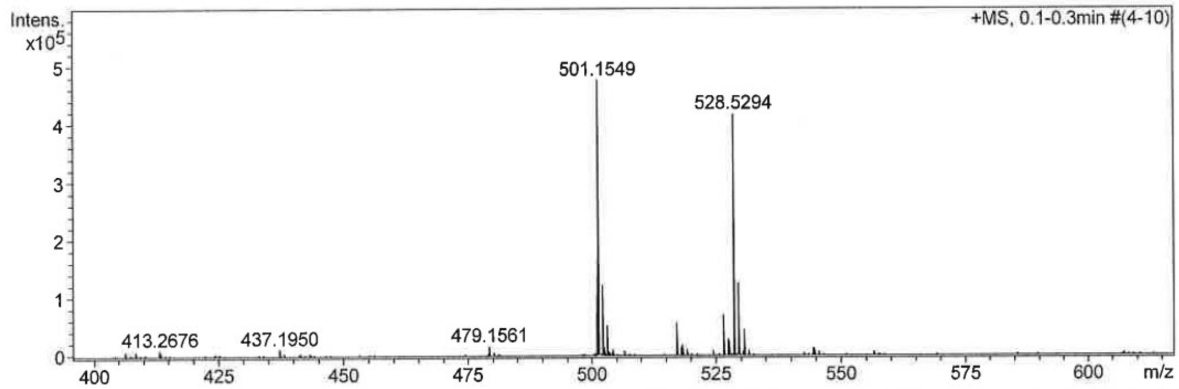

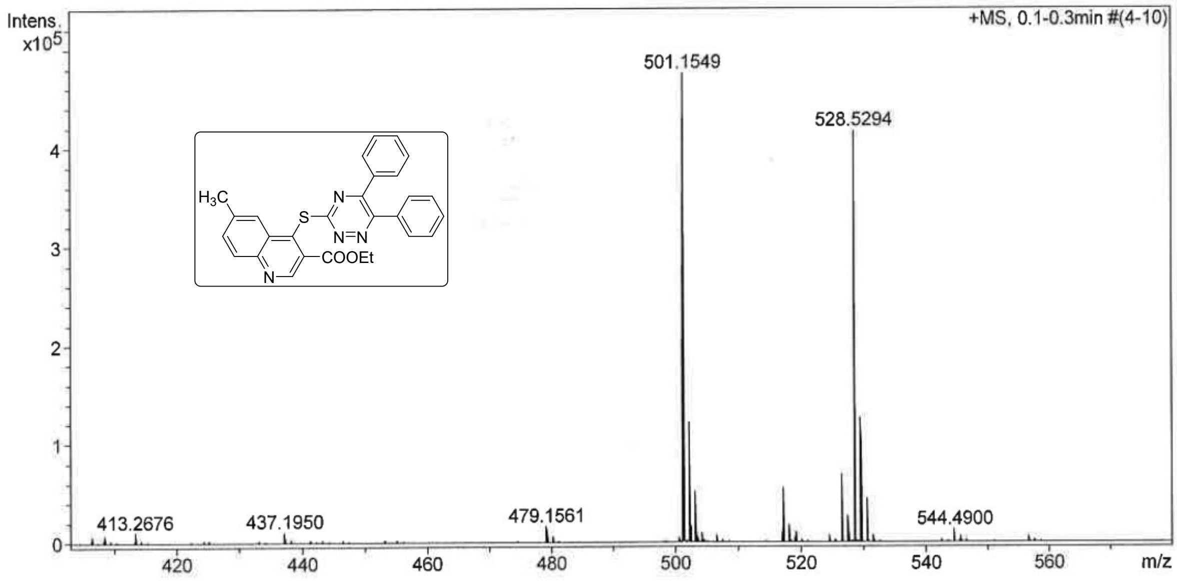

Figure S9: Mass spectrum of Molecule (T3)

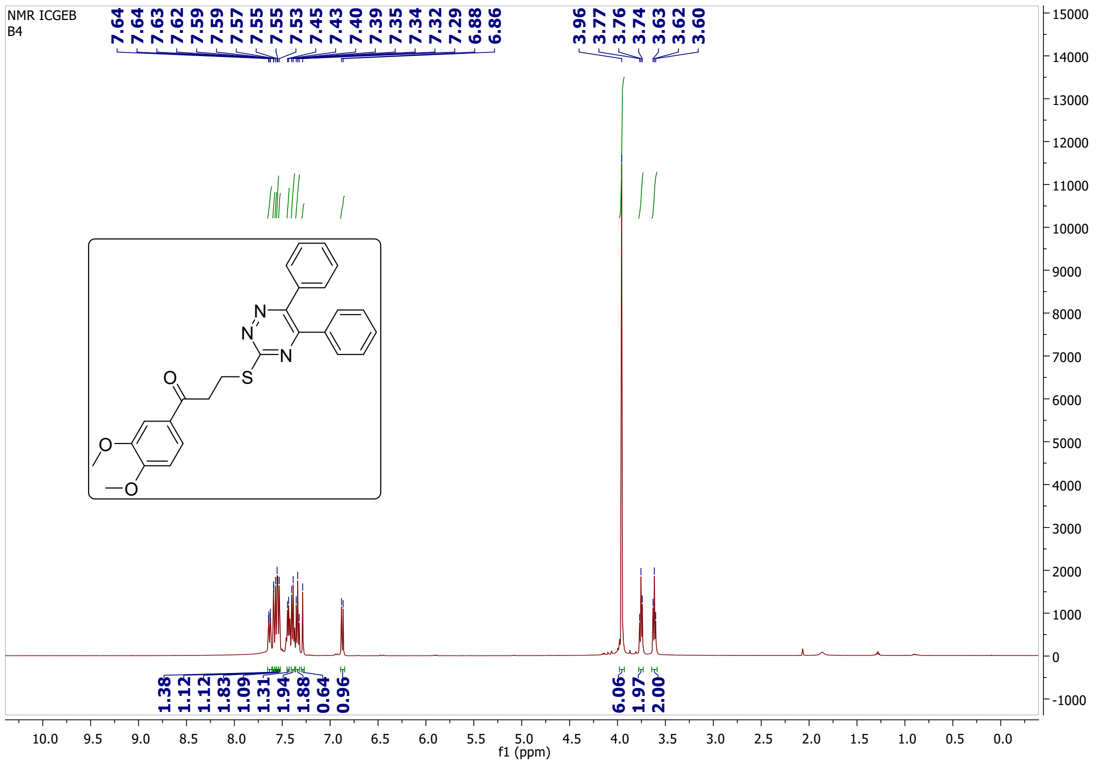

Figure S10: ^{1}H NMR spectrum of Molecule (T4)

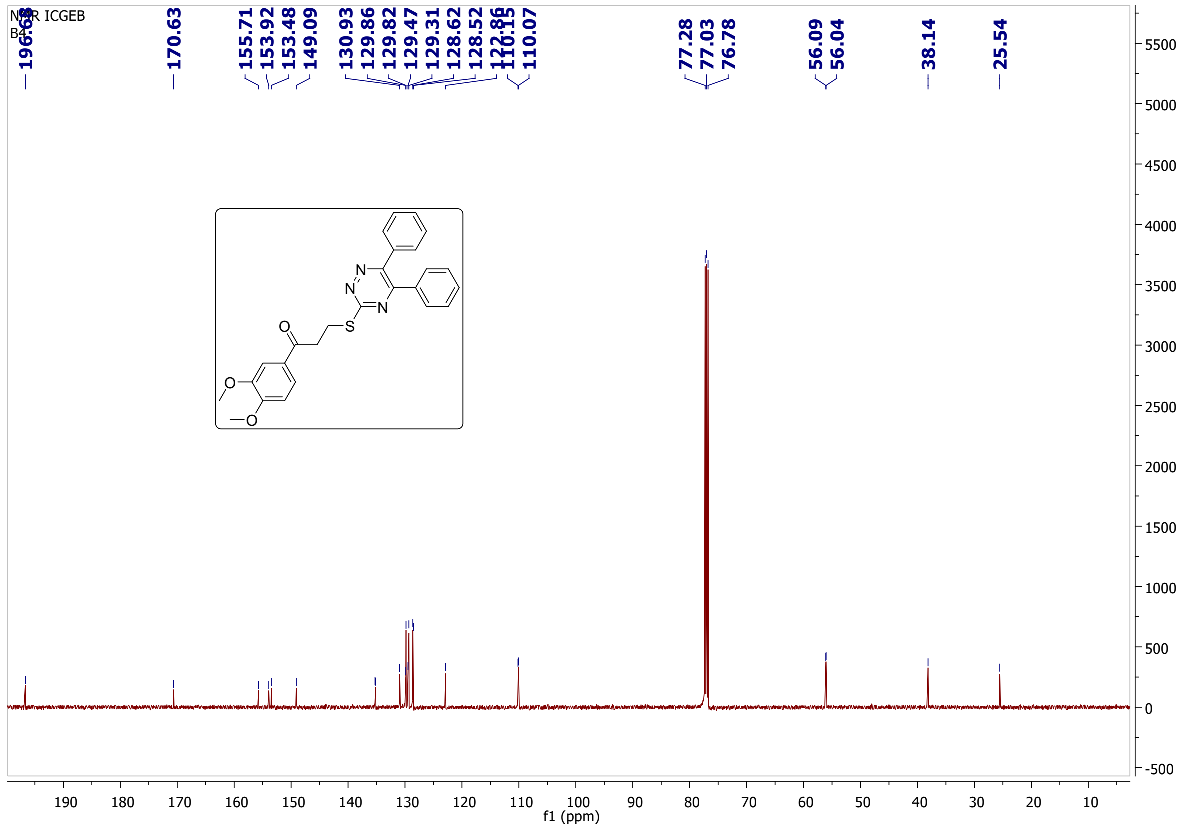

Figure S11: ^{13}C NMR spectrum of Molecule (T4)

Generic Display Report

Analysis Info

Analysis Name	D:\Data\yang jh\20190210-B4.d	Acquisition Date	2/10/2019 2:08:08 PM
Method	20170112_pos_50-1000.m	Operator	Bruker07
Sample Name	B4-1	Instrument	micrOTOF-Q
Comment			

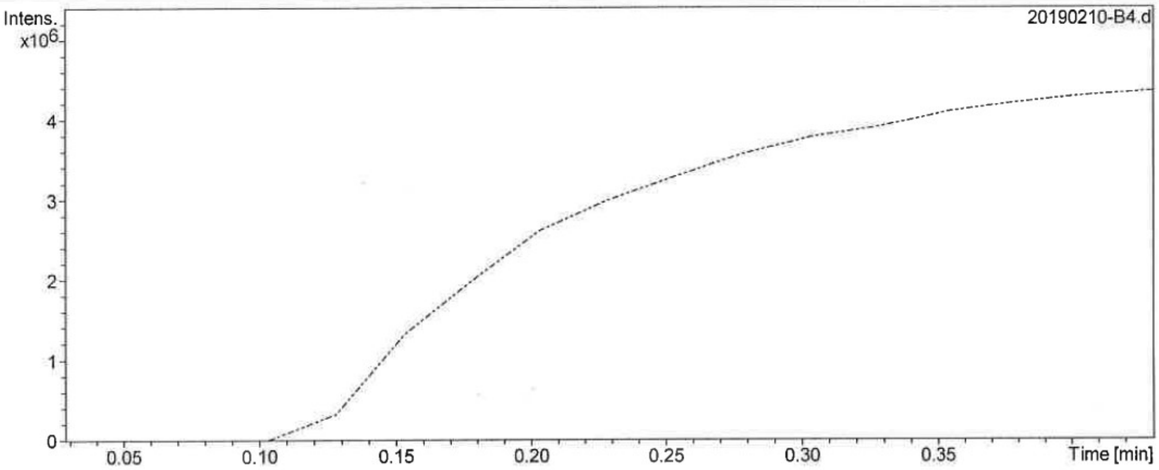

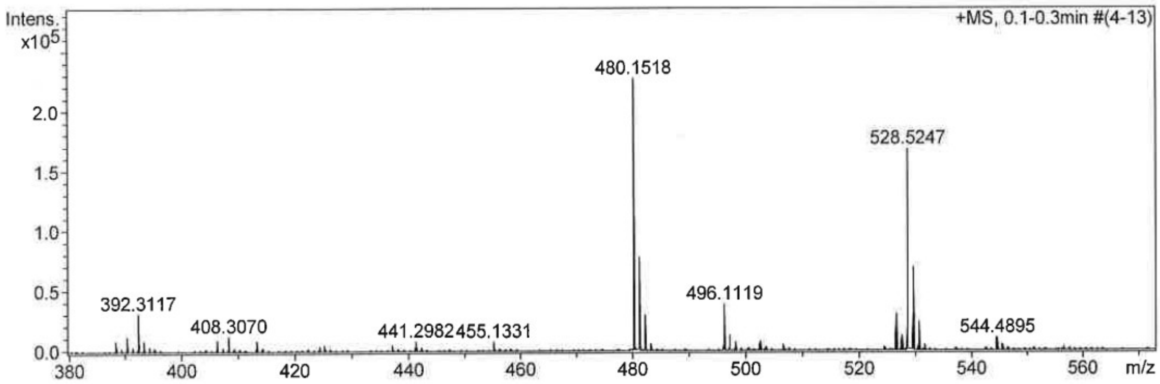

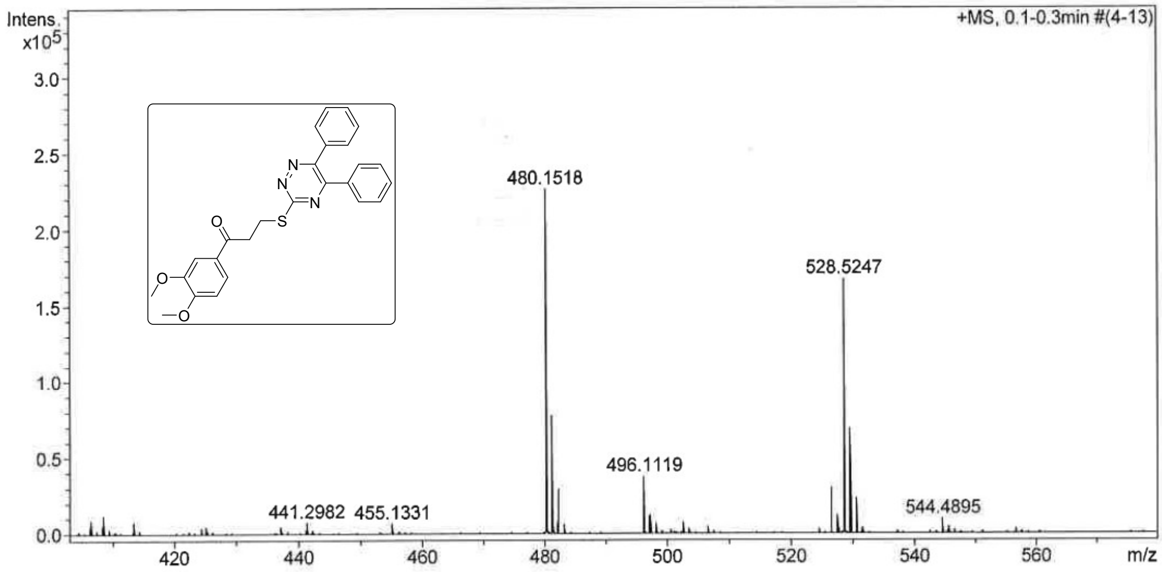

Figure S12: Mass spectrum of Molecule (T4)

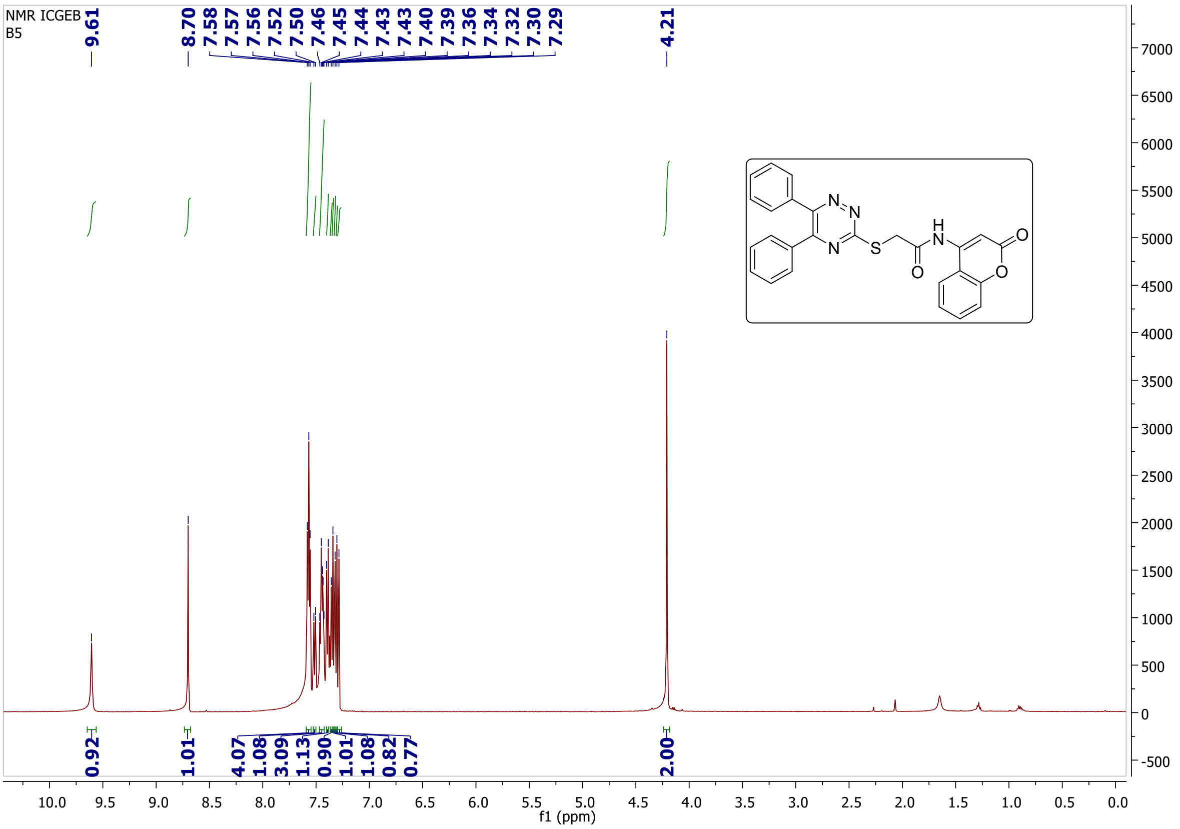

Figure S13: ^{1}H NMR spectrum of Molecule (T5)

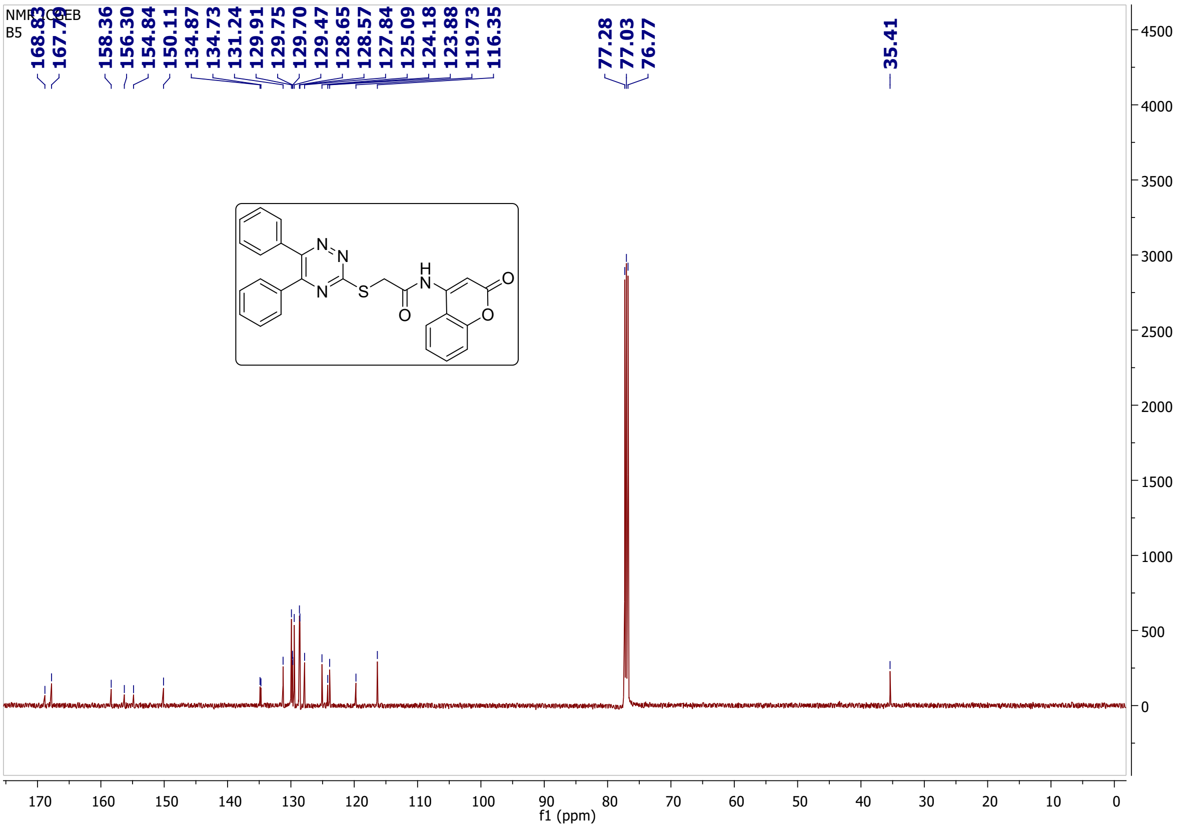

Figure S14: ^{13}C NMR spectrum of Molecule (T5)

Generic Display Report

Analysis Info

Analysis Name	D:\Data\yang jh\20190210-B5.d	Acquisition Date	2/10/2019 2:15:21 PM
Method	20170112_pos_50-1000.m	Operator	Bruker07
Sample Name	B5-1	Instrument	micrOTOF-Q
Comment			

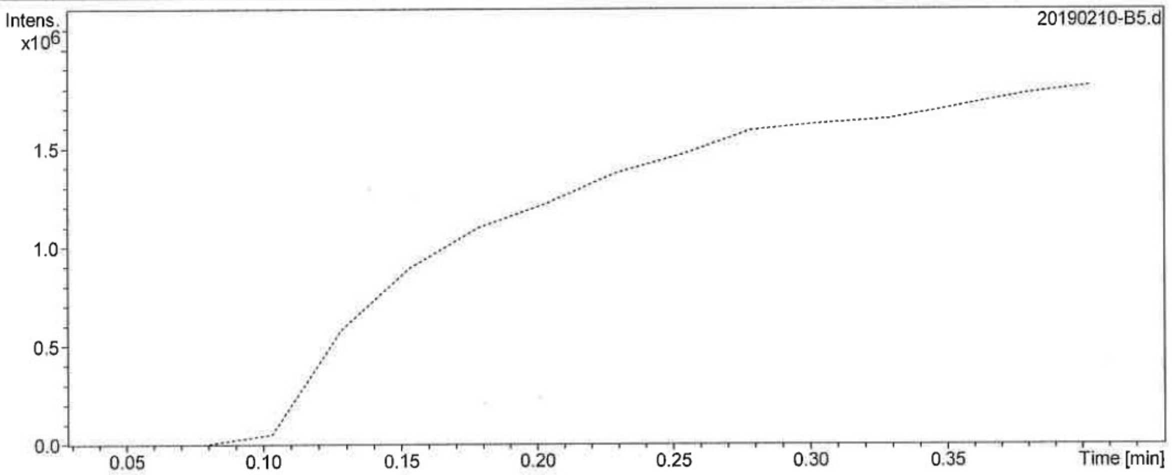

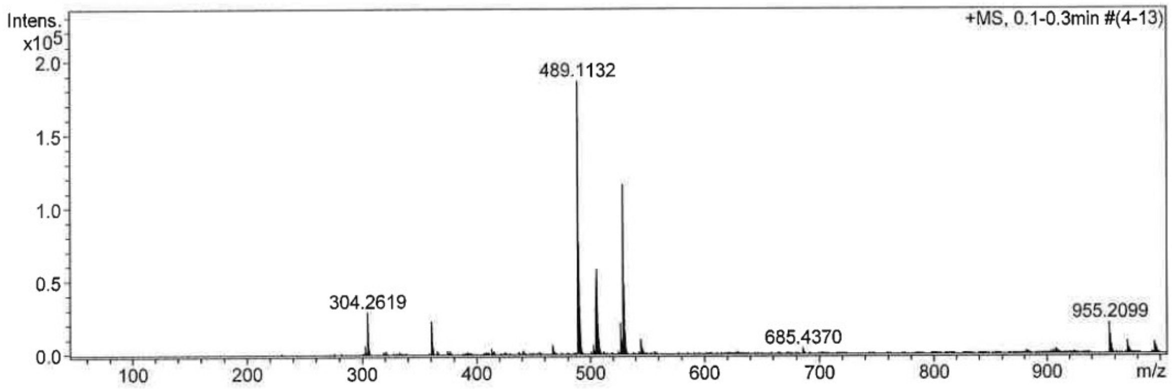

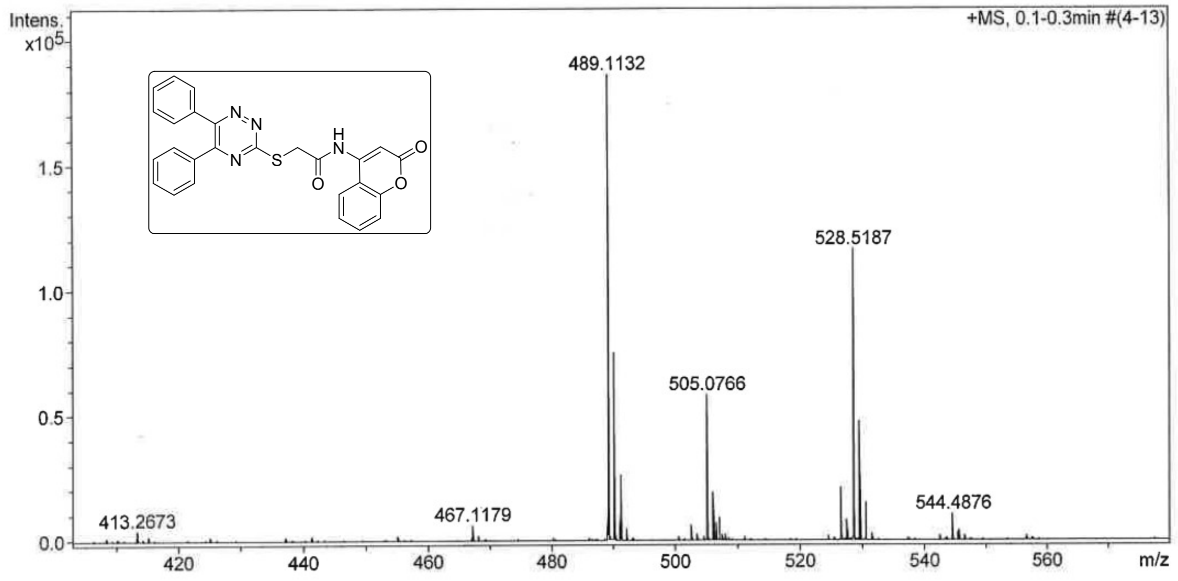

Figure S15: Mass spectrum of Molecule (T5)

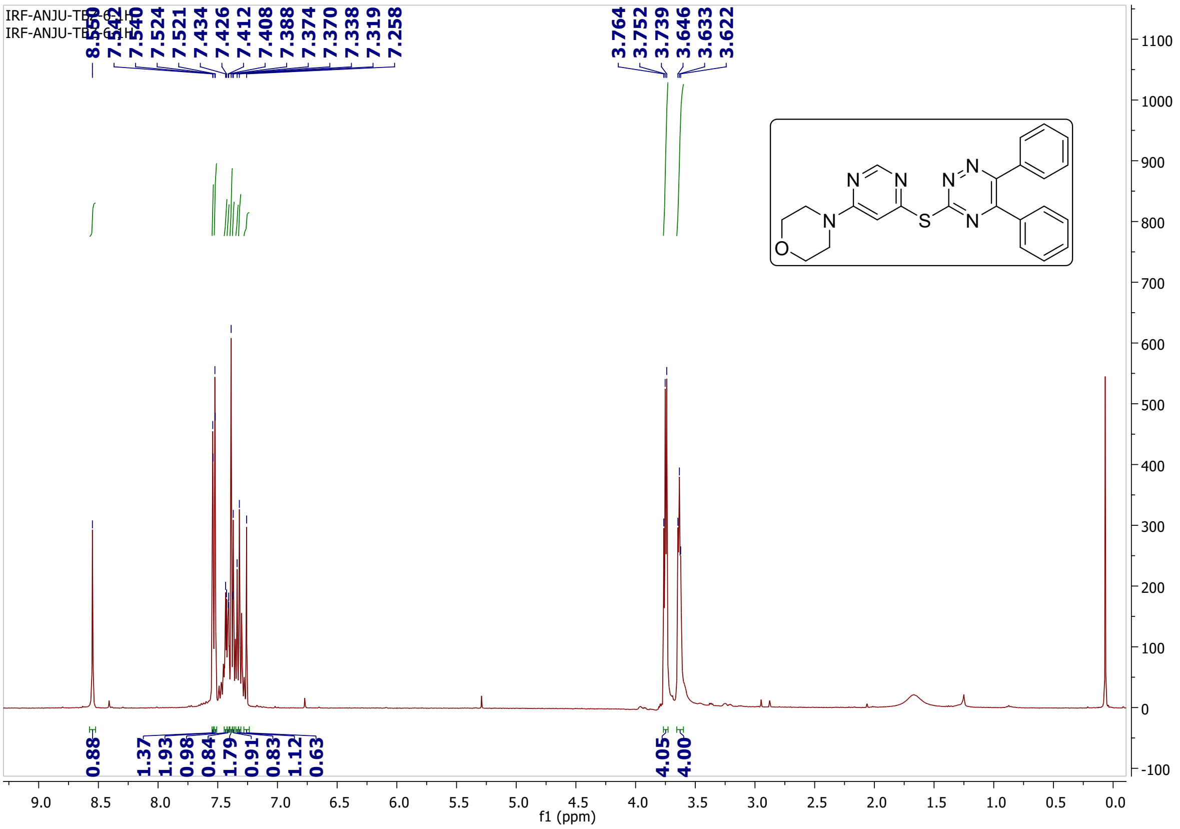

Figure S16: ^{1}H NMR spectrum of Molecule (T6)

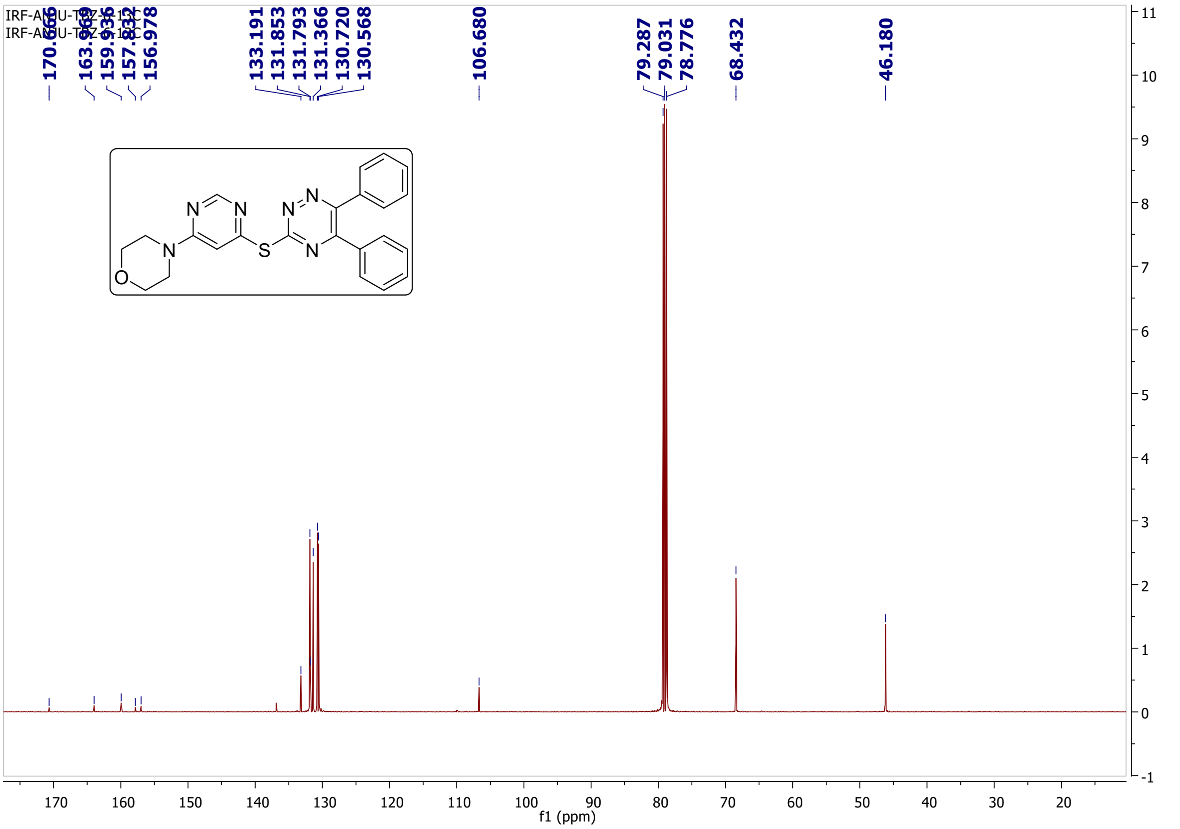

Figure S17: ^{13}C NMR spectrum of Molecule (T6)

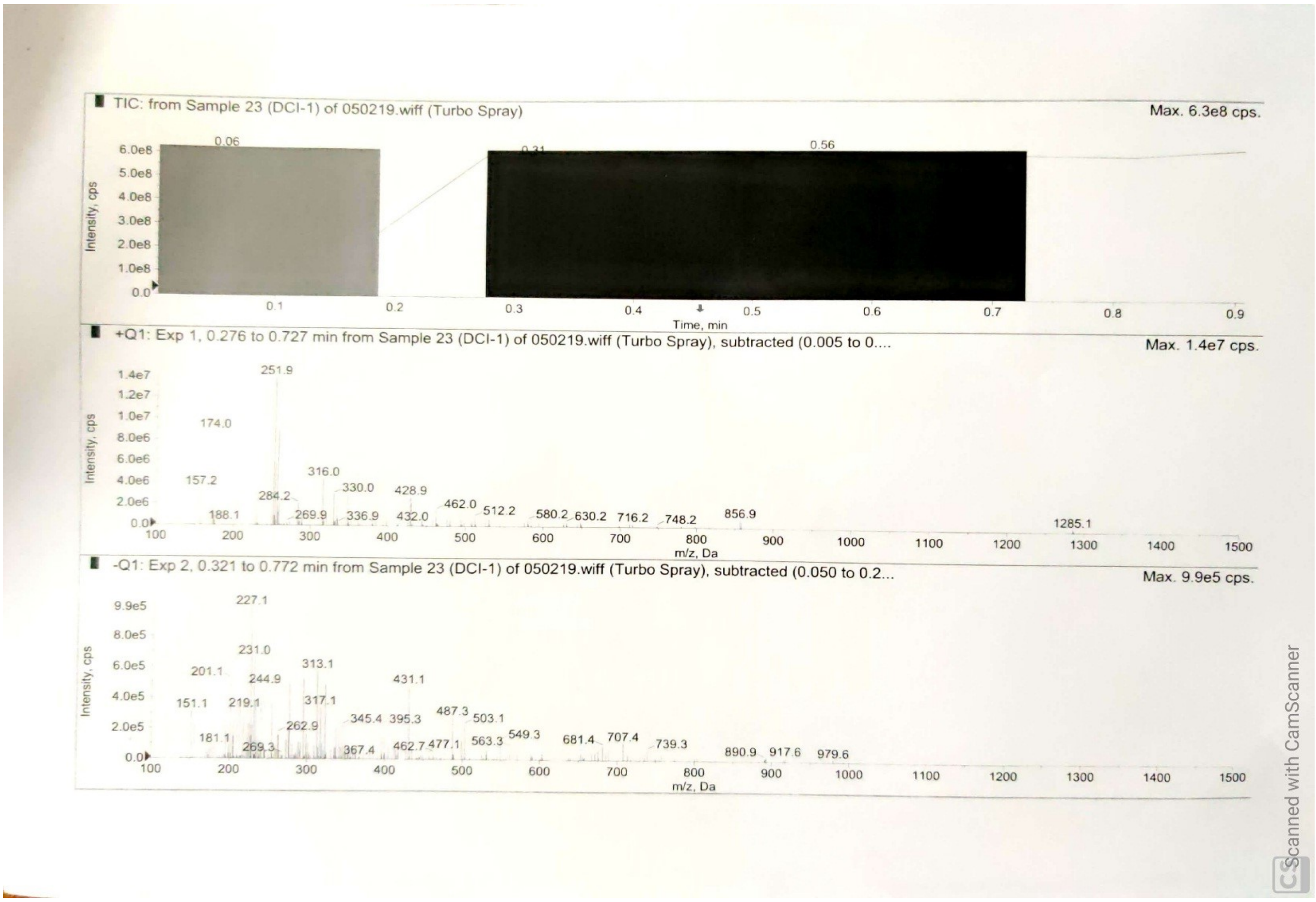

Figure S18: Mass spectrum of Molecule (T6)

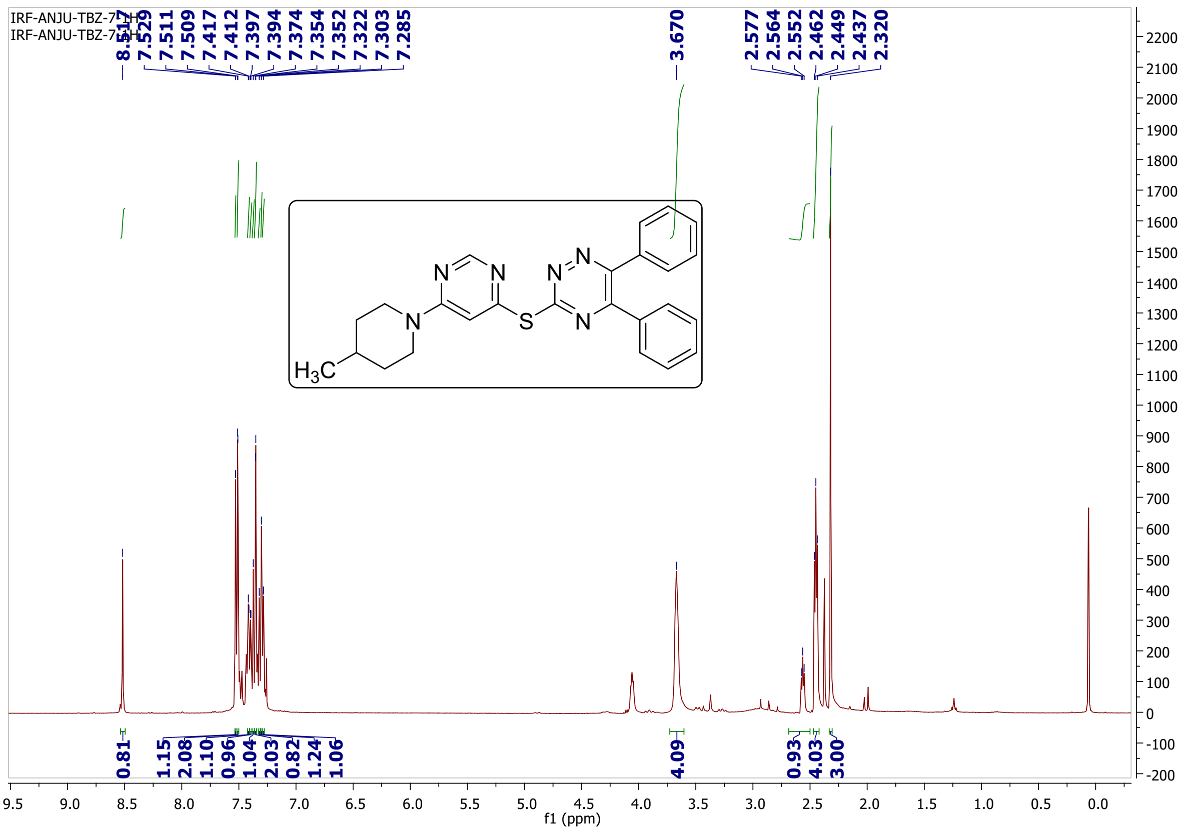

Figure S19: ^{1}H NMR spectrum of Molecule (T7)

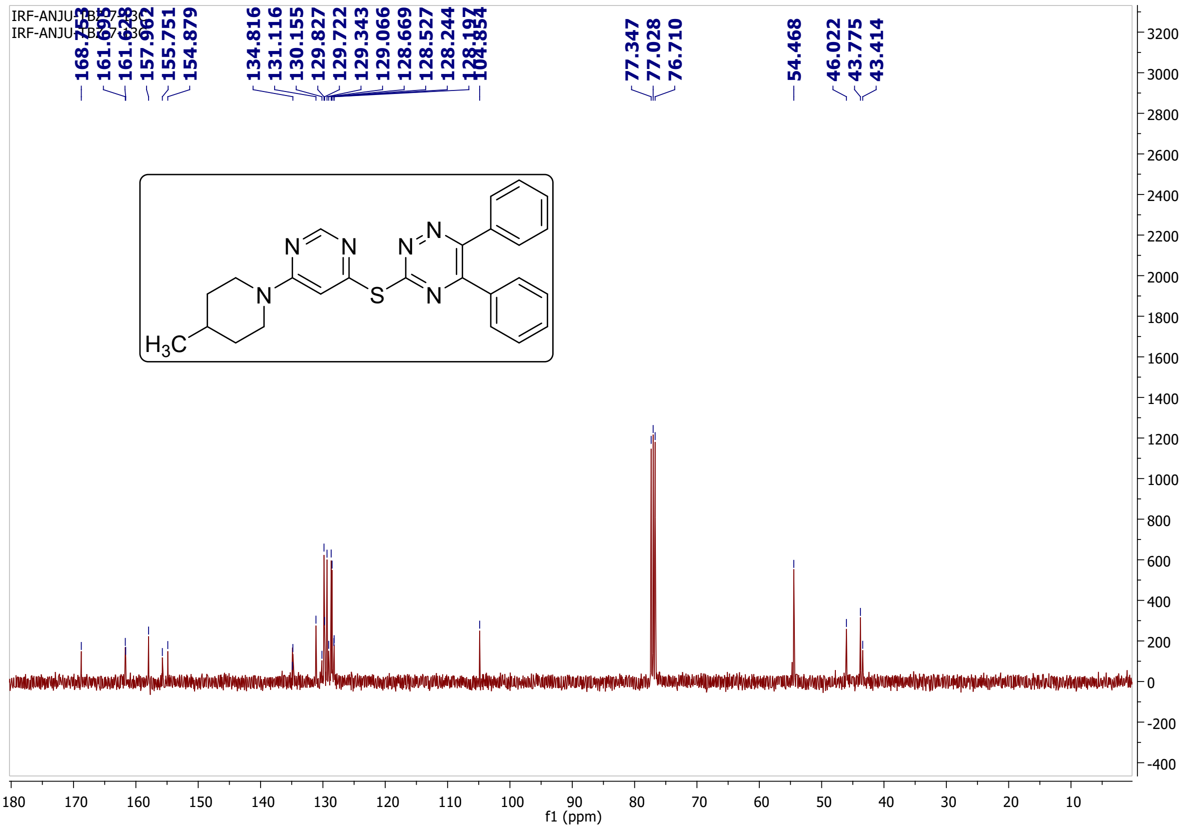

Figure S20: ^{13}C NMR spectrum of Molecule (T7)

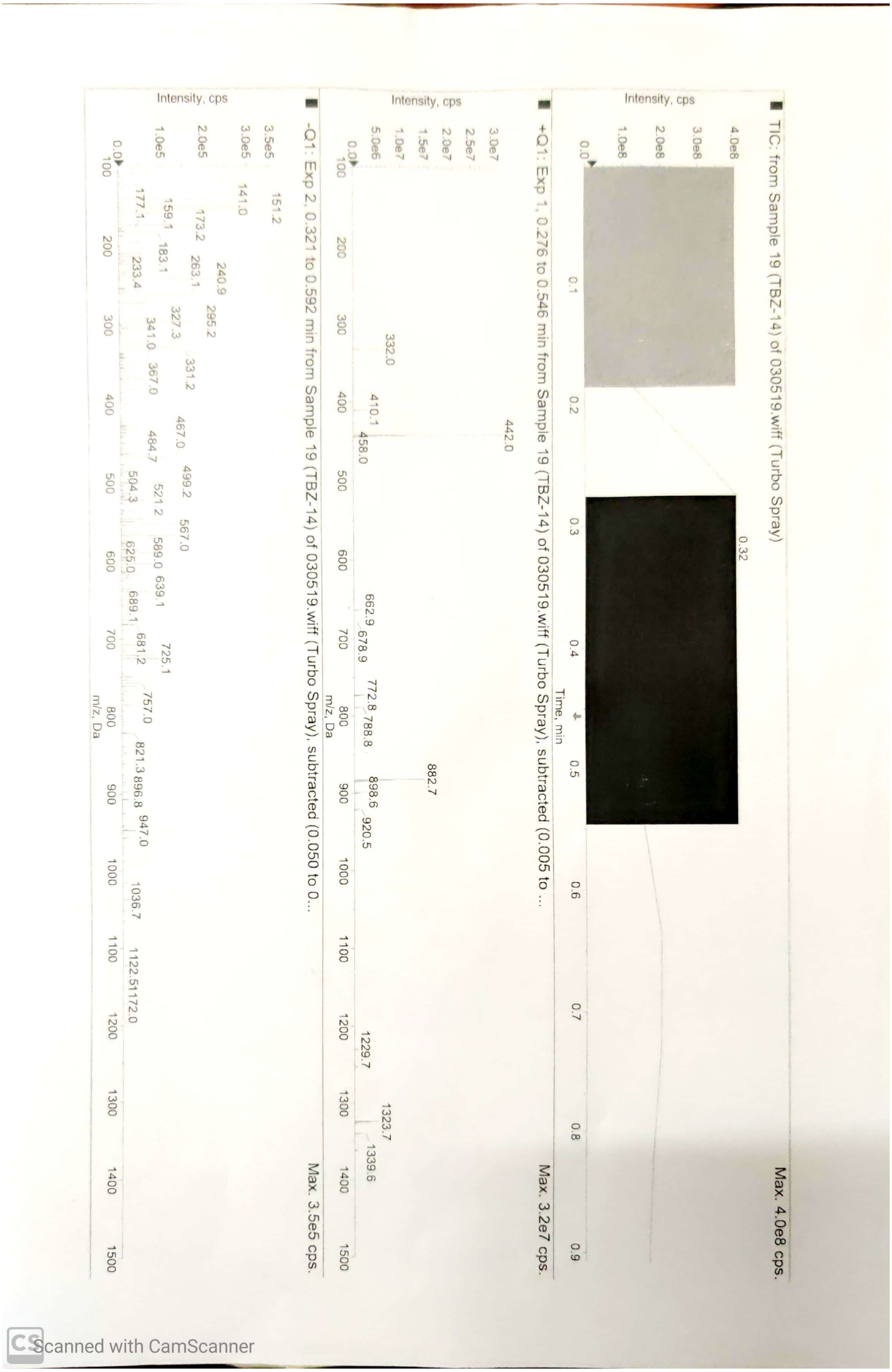

Figure S21: Mass spectrum of Molecule (T7)

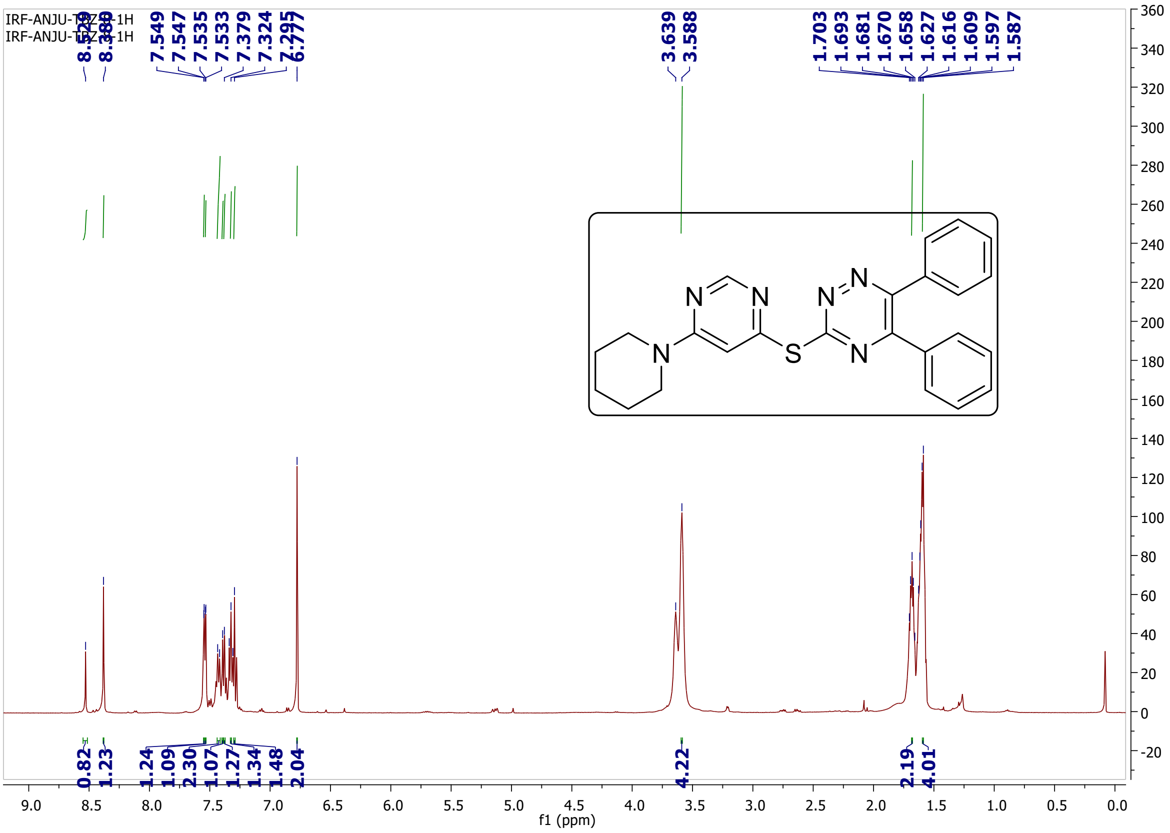

Figure S22: ^{1}H NMR spectrum of Molecule (T8)

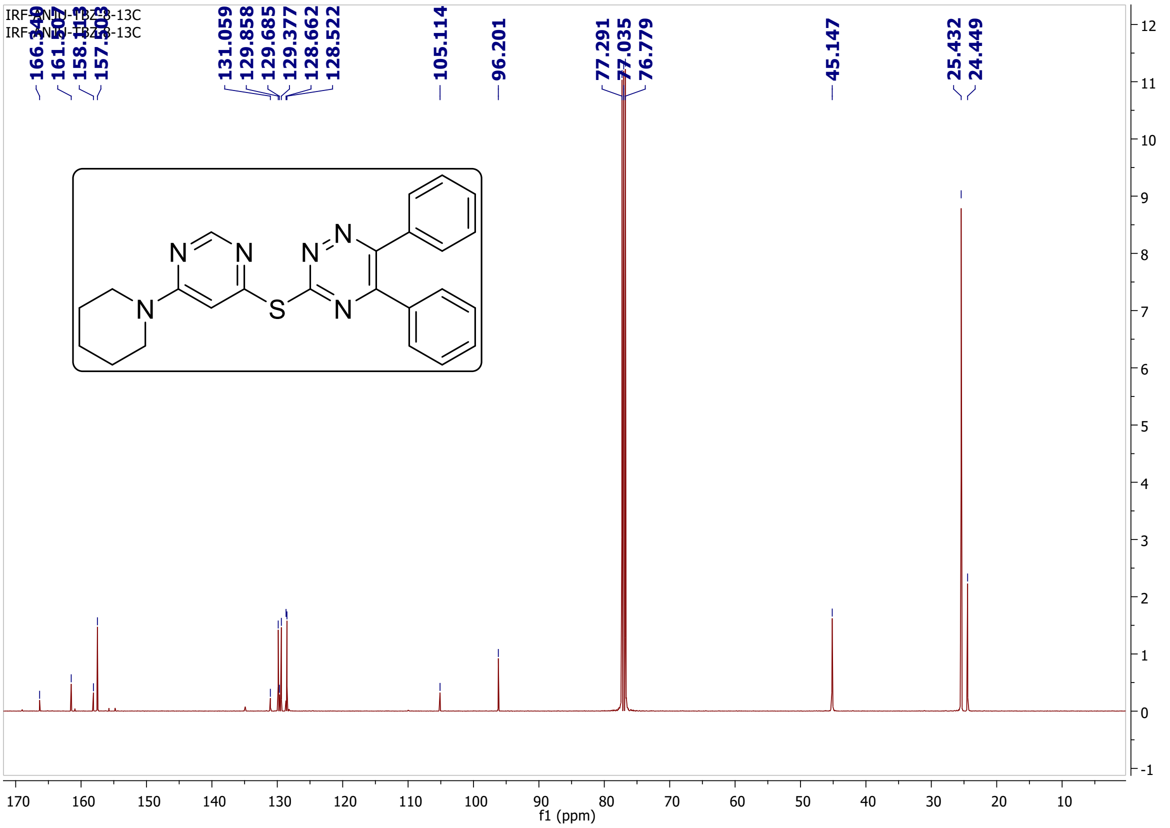

Figure S23: ^{13}C NMR spectrum of Molecule (T8)

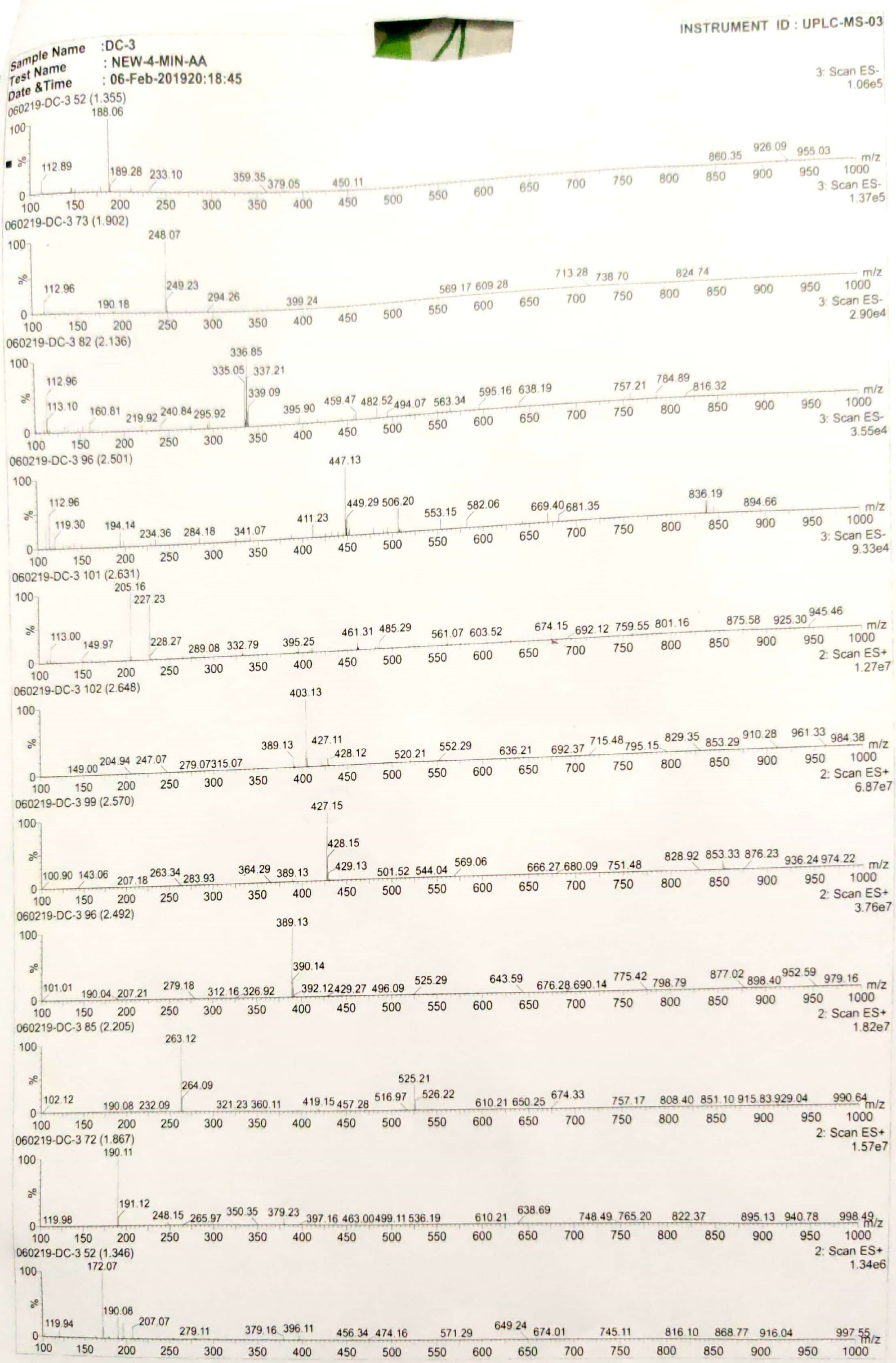

Scanned with CamScanner

Figure S24: Mass spectrum of Molecule (T8)

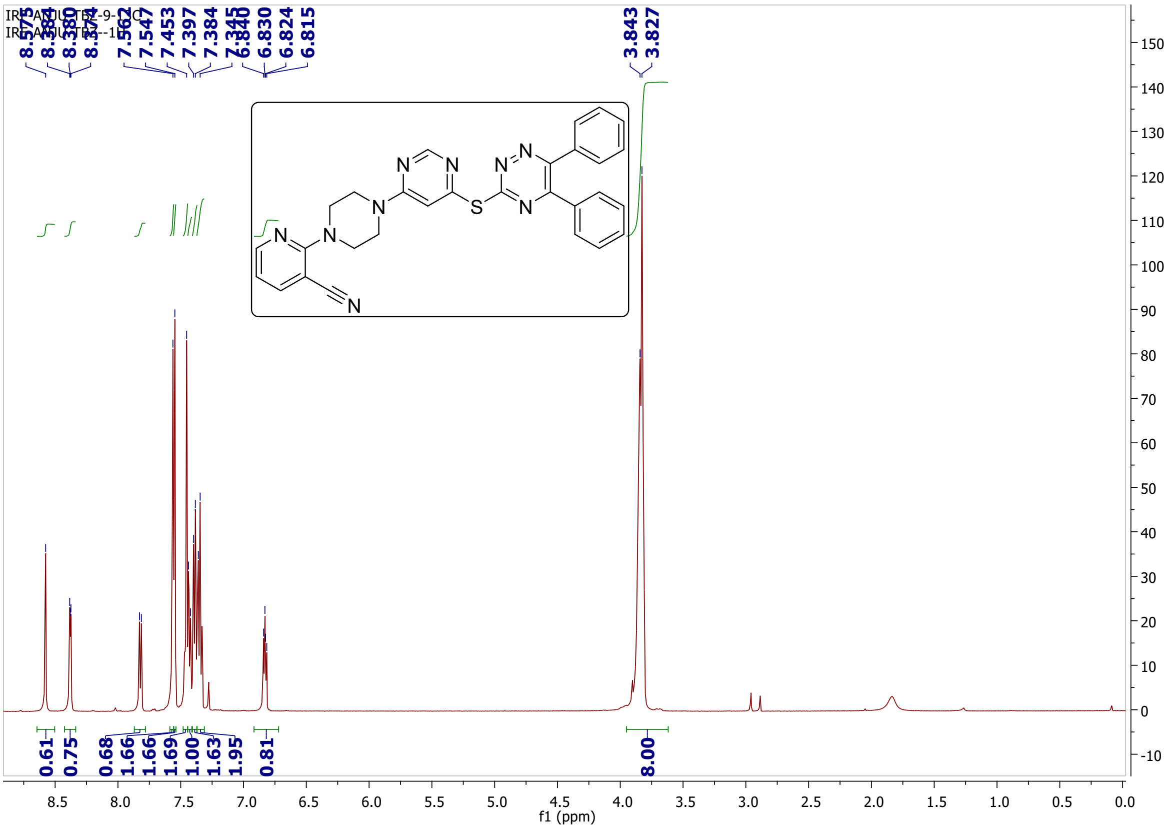

Figure S25: ^{1}H NMR spectrum of Molecule (T9)

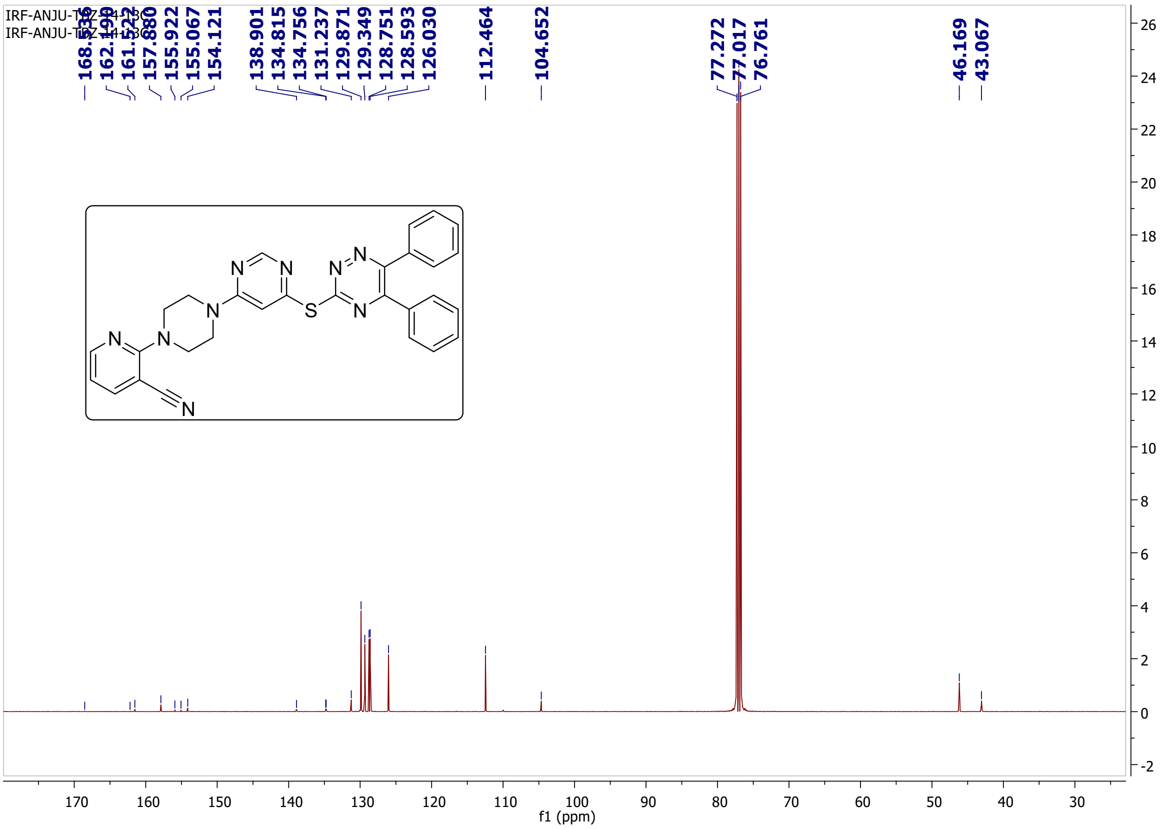

Figure S26: ^{13}C NMR spectrum of Molecule (T9)

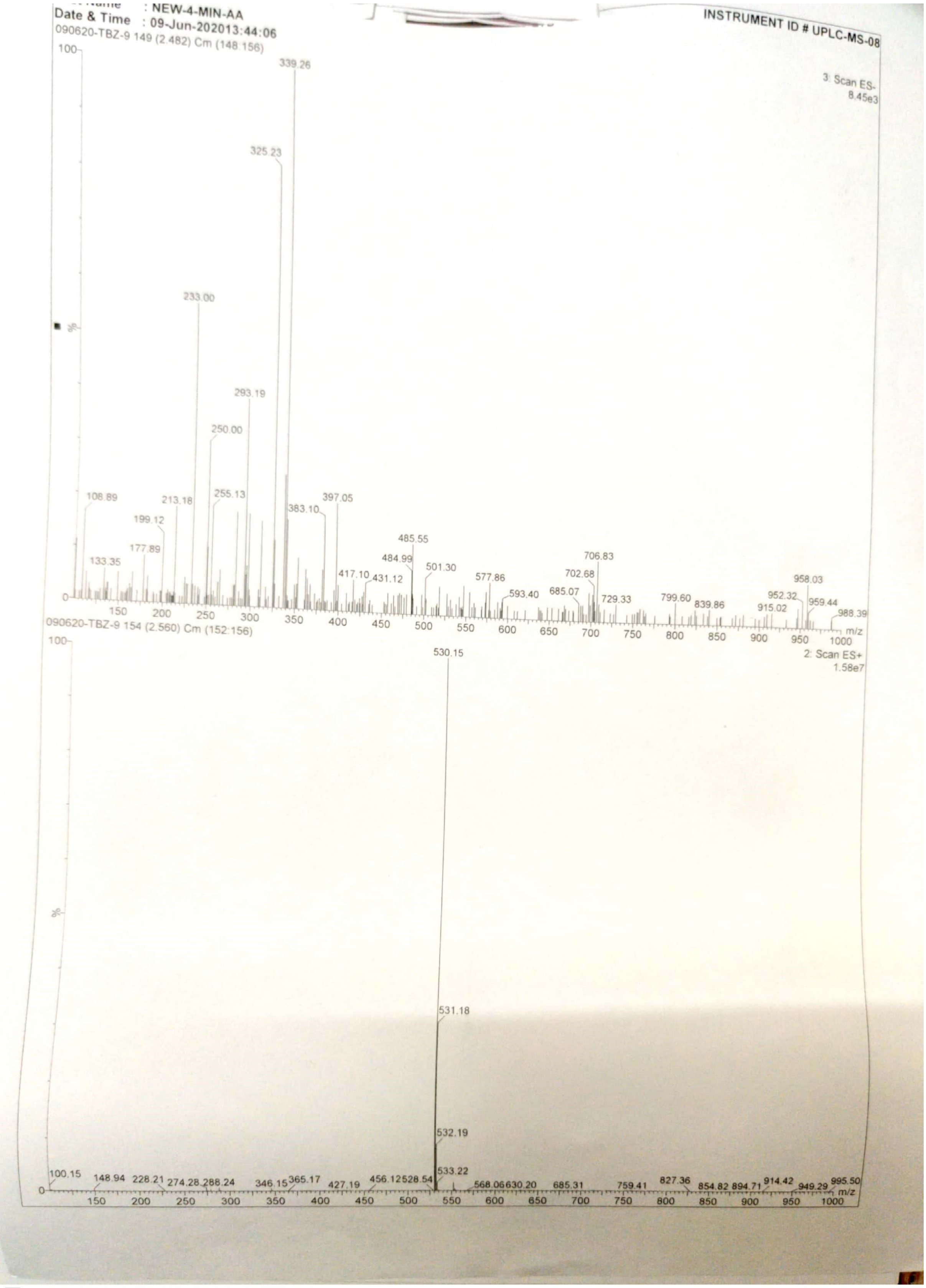

Scanned with CamScanner

Figure S27: Mass spectrum of Molecule (T9)

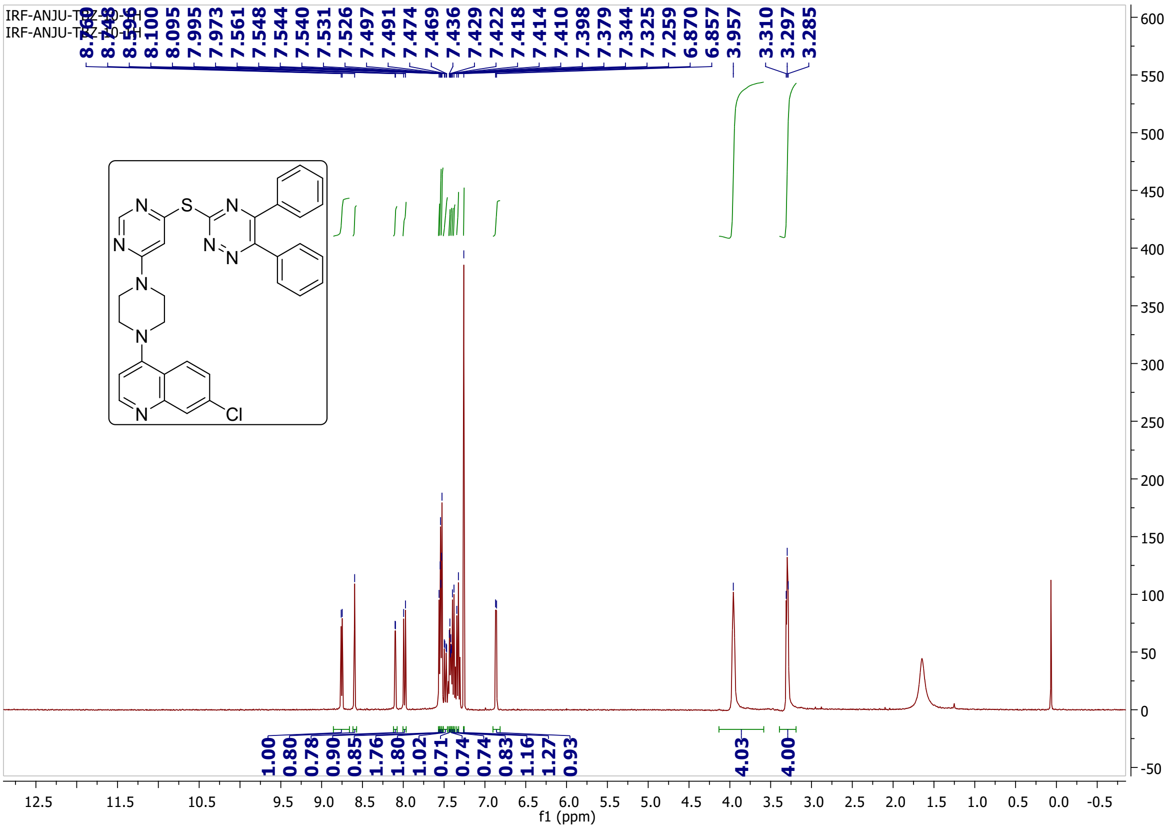

Figure S28: ^{1}H NMR spectrum of Molecule (T10)

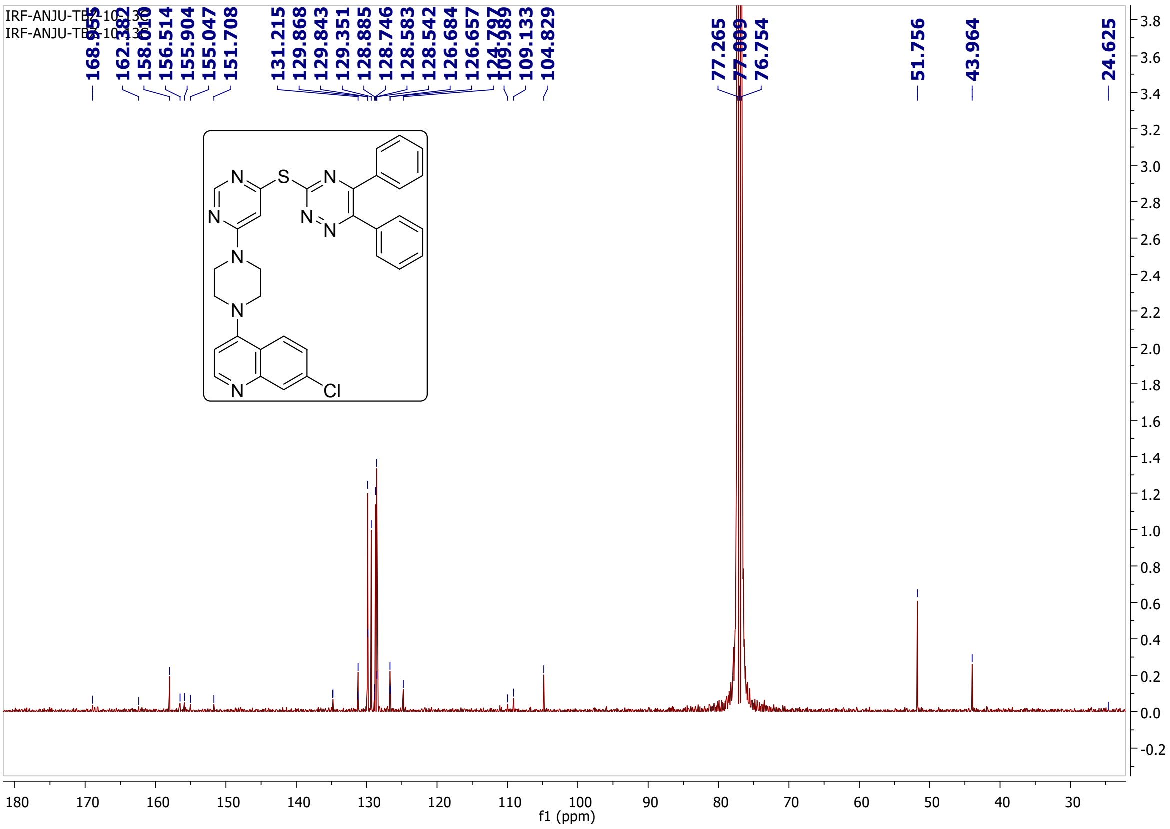

Figure S29: ^{13}C NMR spectrum of Molecule (T10)

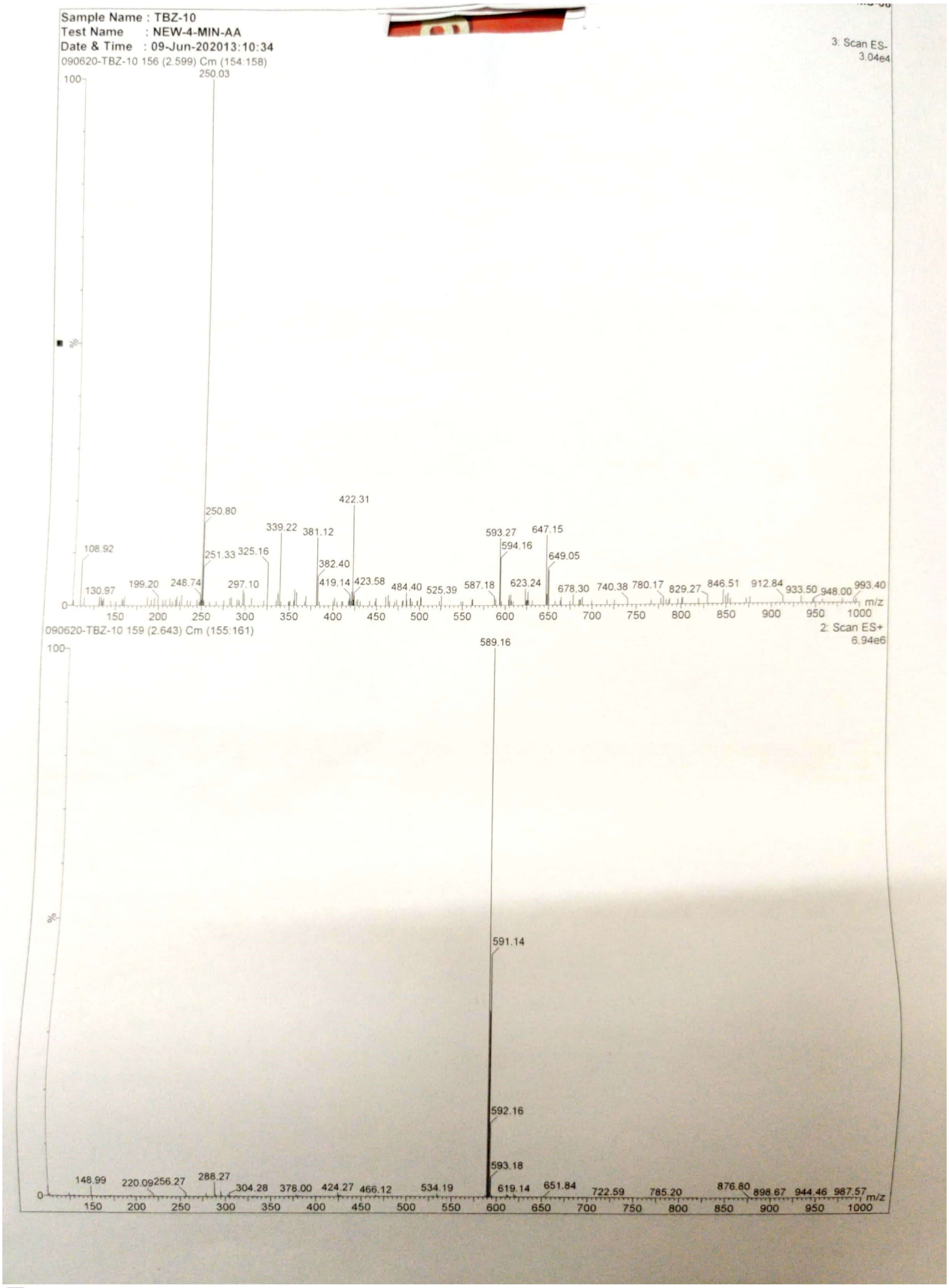

Scanned with CamScanner

Figure S30: Mass spectrum of Molecule (T10)

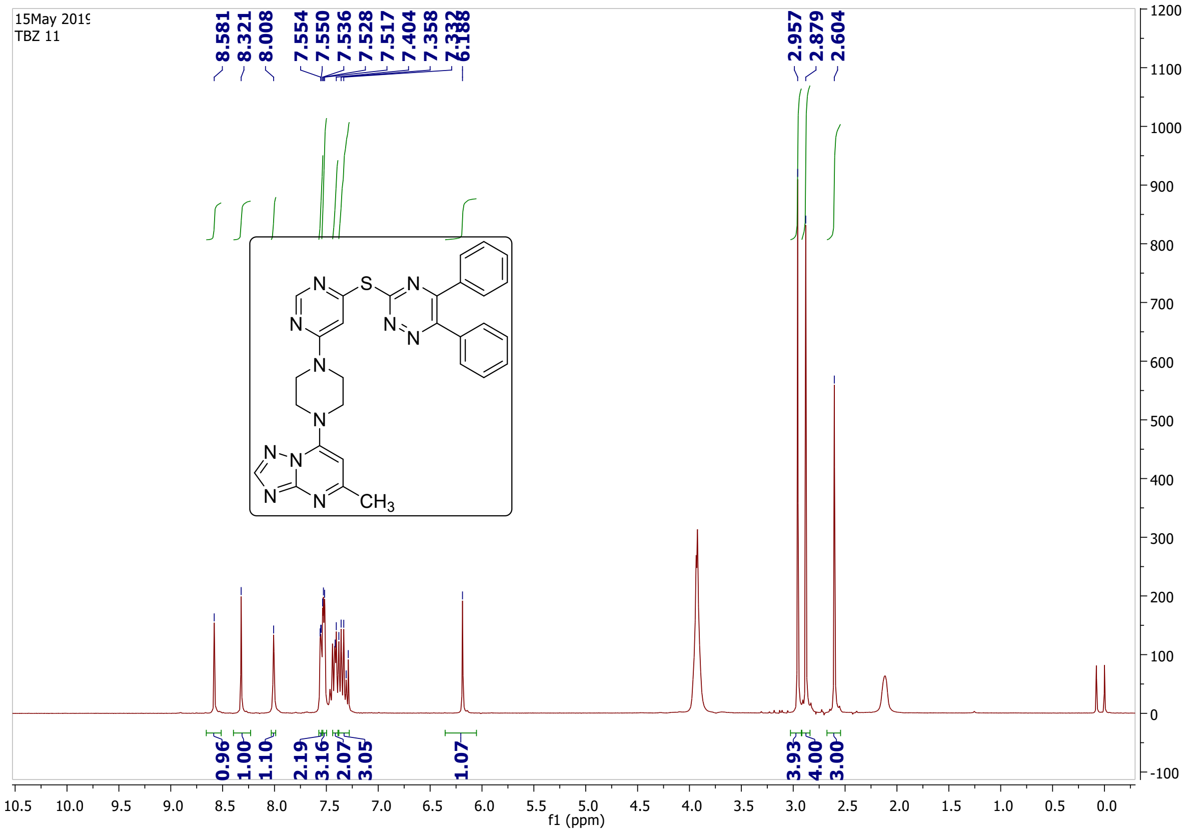

Figure S31: ^{1}H NMR spectrum of Molecule (T11)

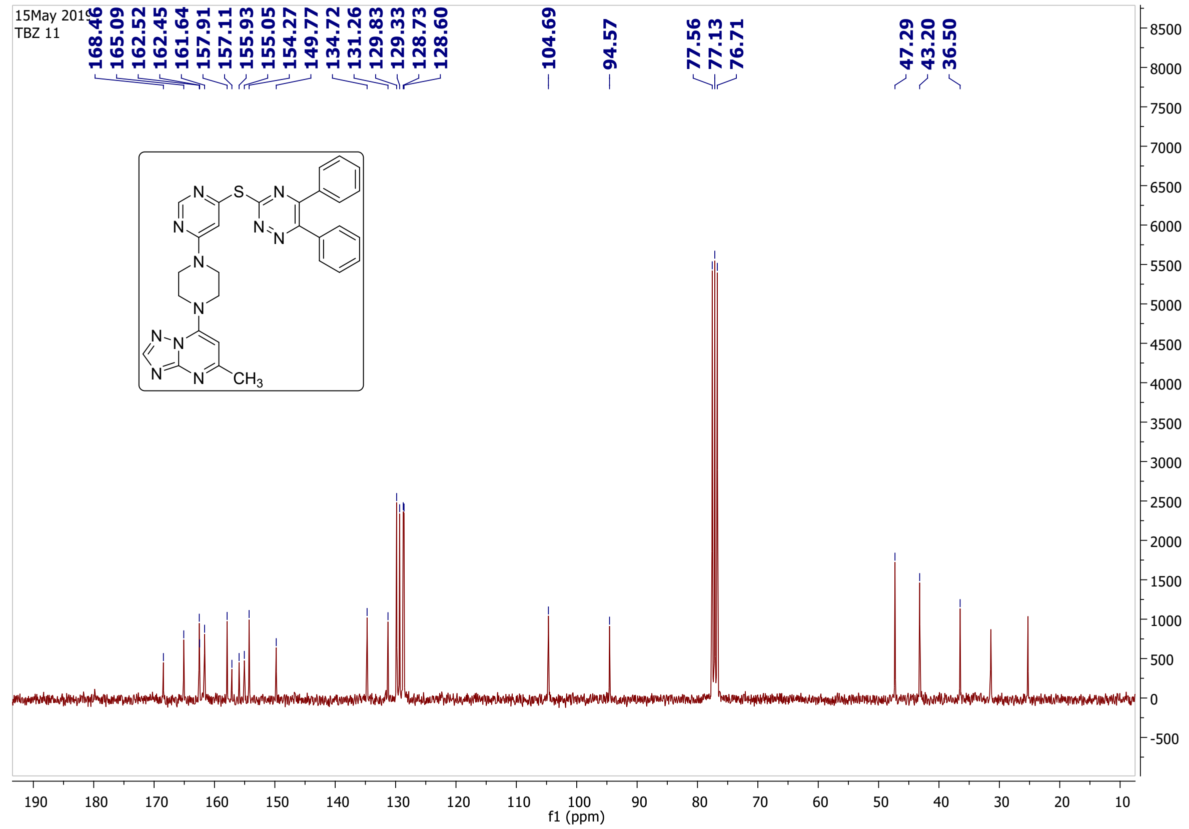

Figure S32: ^{13}C NMR spectrum of Molecule (T11)

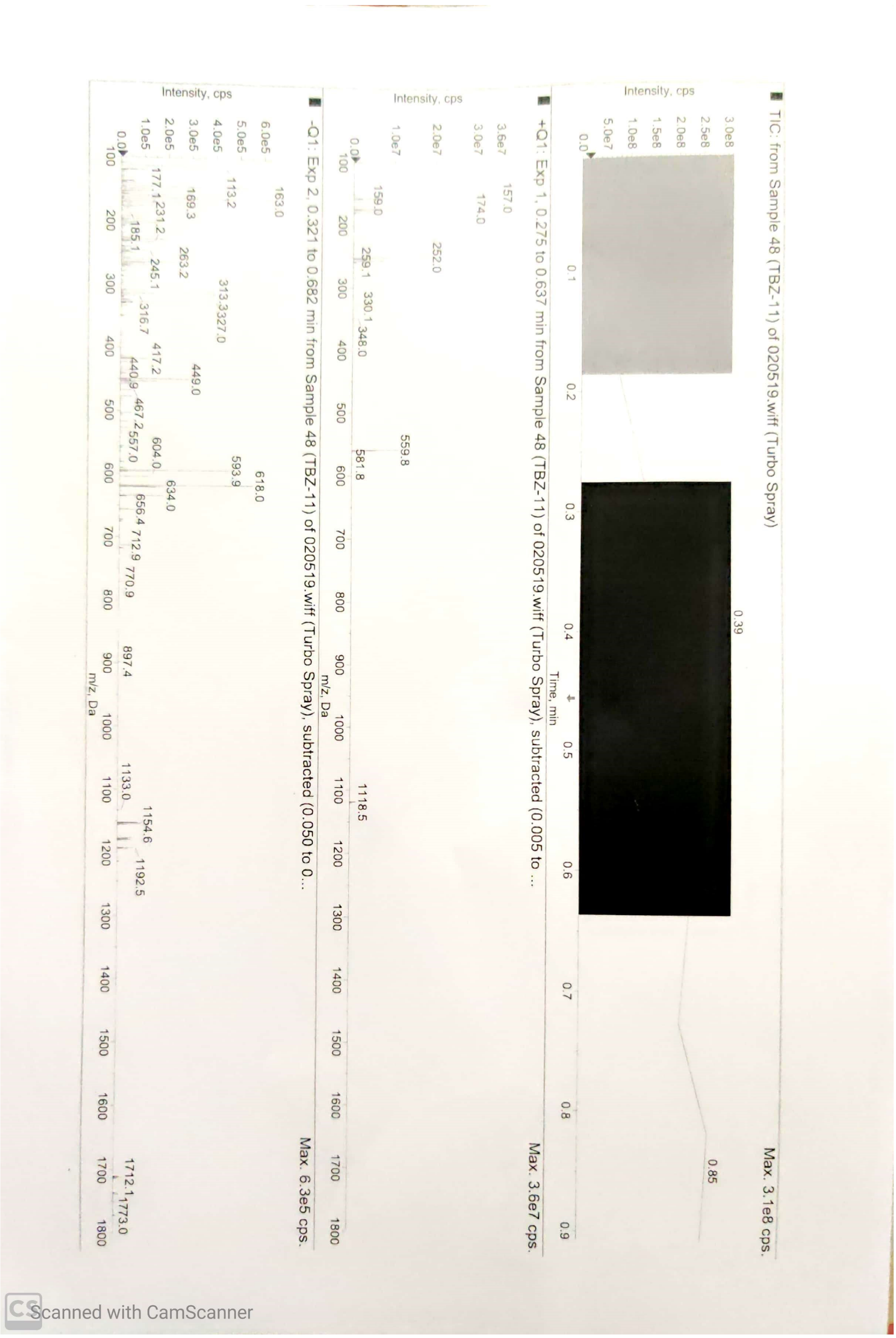

Figure S33: Mass spectrum of Molecule (T11)

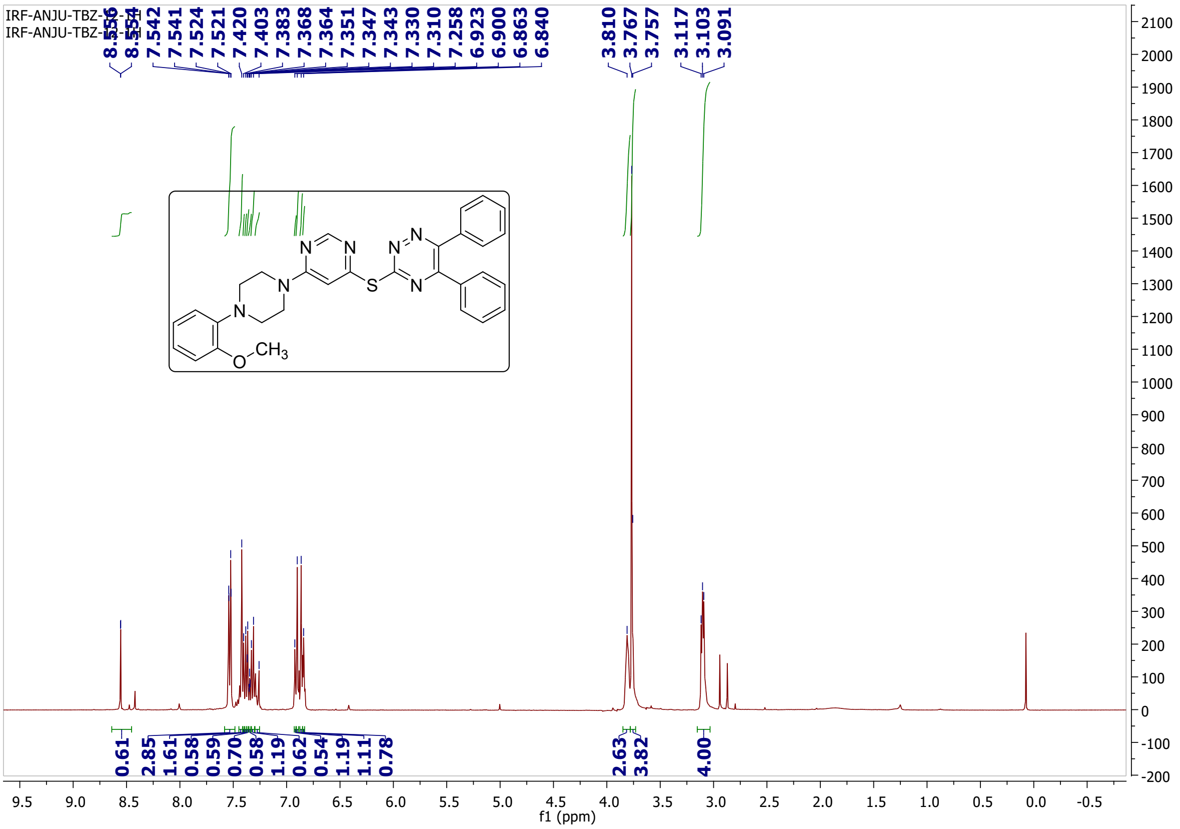

Figure S34: ^{1}H NMR spectrum of Molecule (T12)

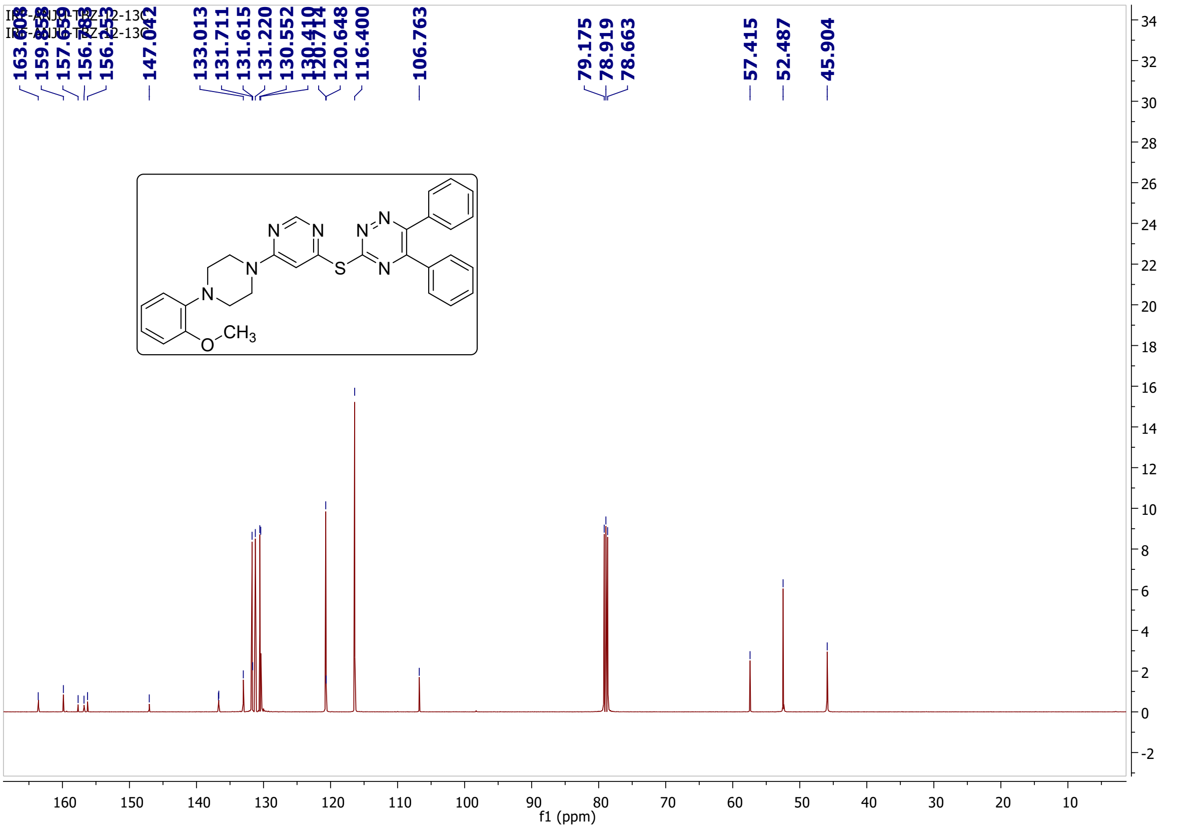

Figure S35: ^{13}C NMR spectrum of Molecule (T12)

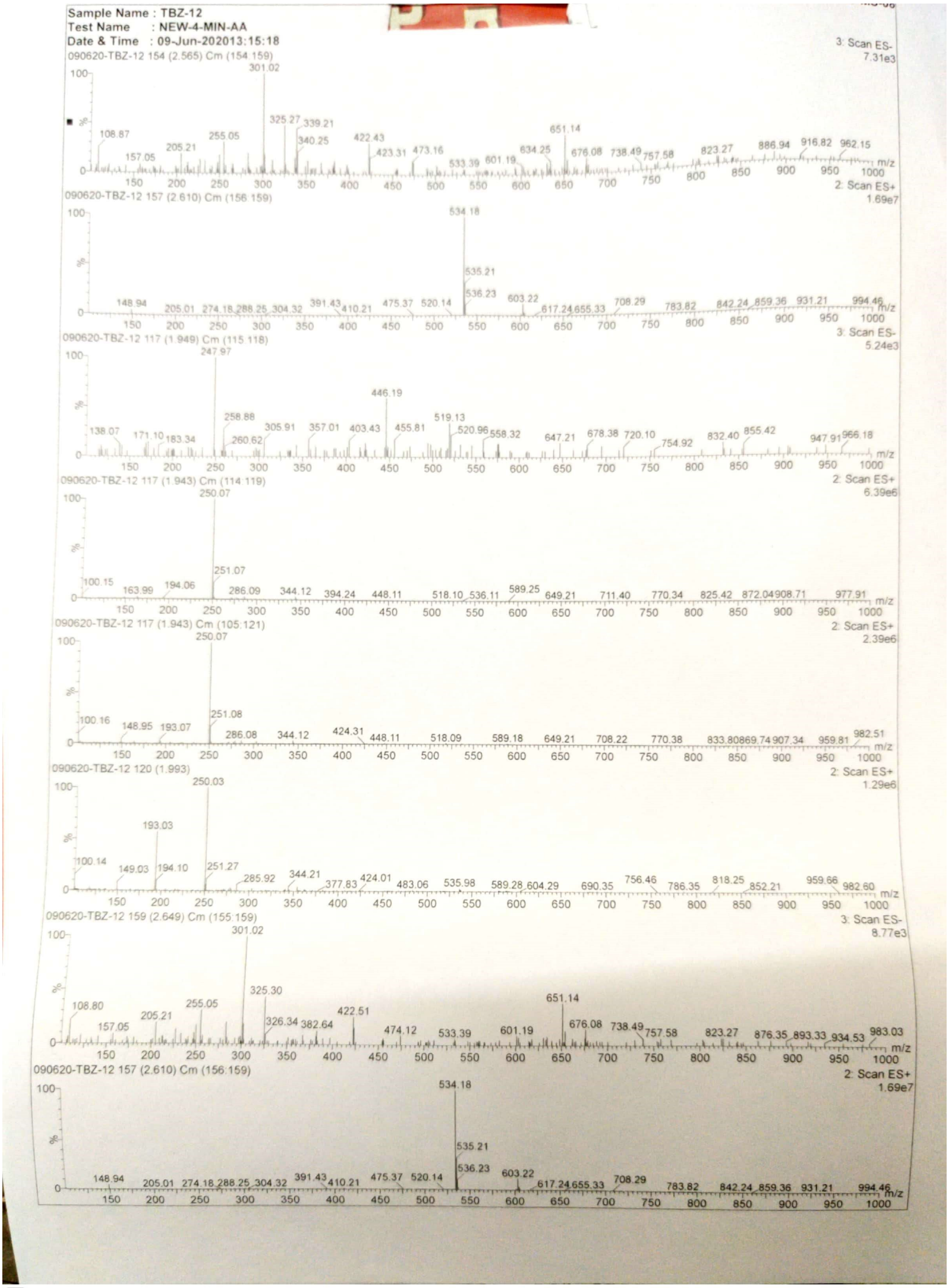

Scanned with CamScanner

Figure S36: Mass spectrum of Molecule (T12)

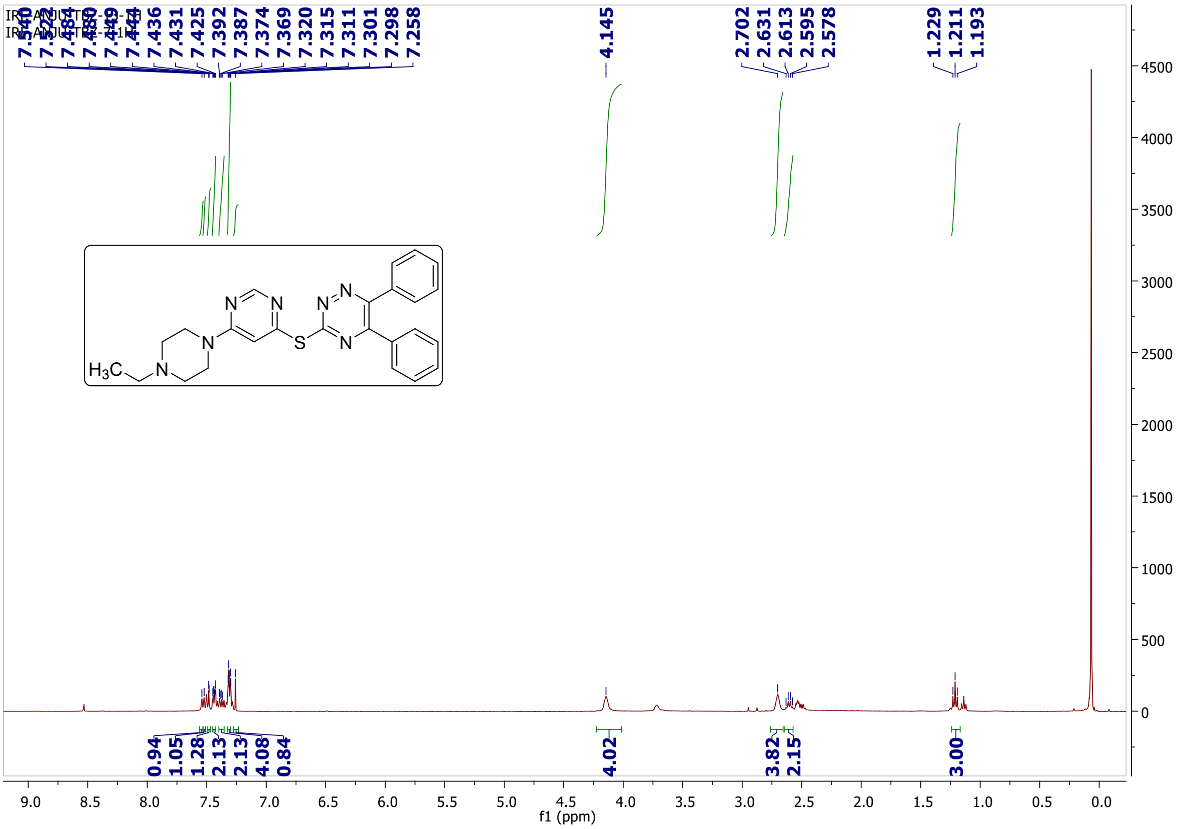

Figure S37: ^{1}H NMR spectrum of Molecule (T13)

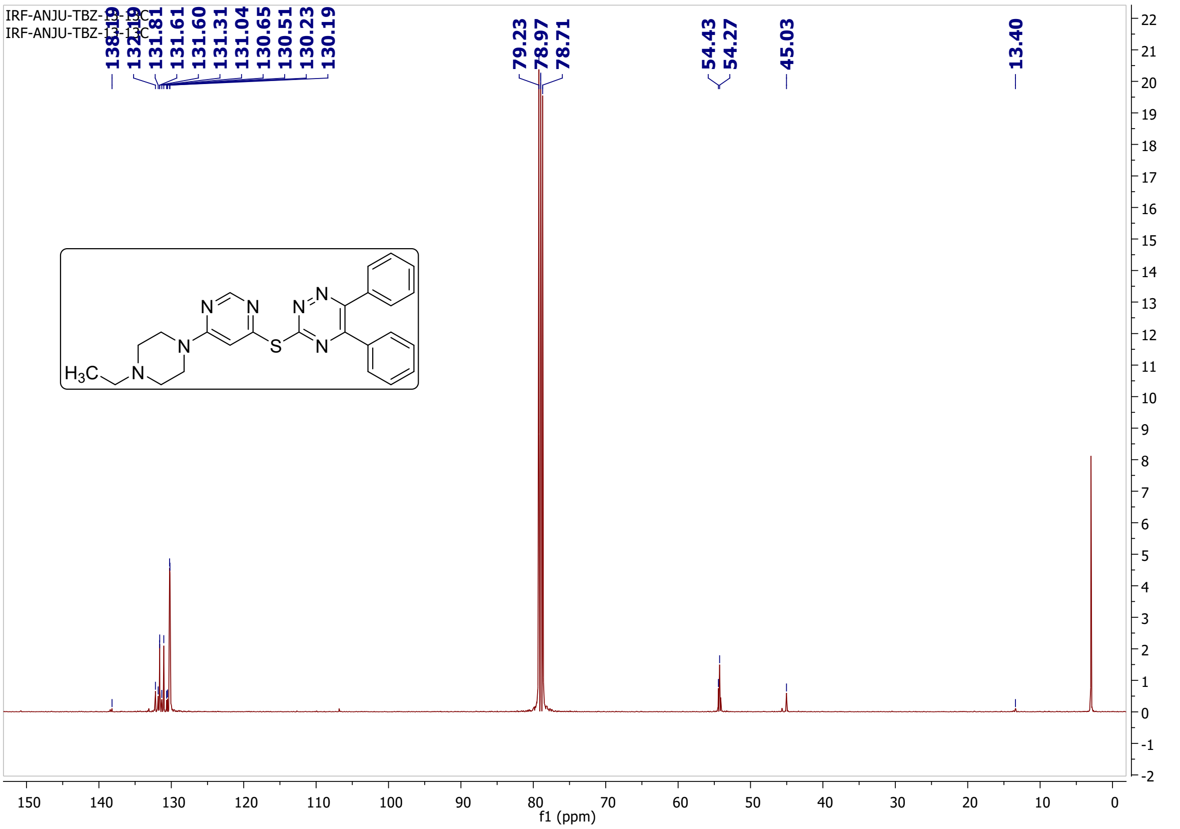

Figure S38: ^{13}C NMR spectrum of Molecule (T13)

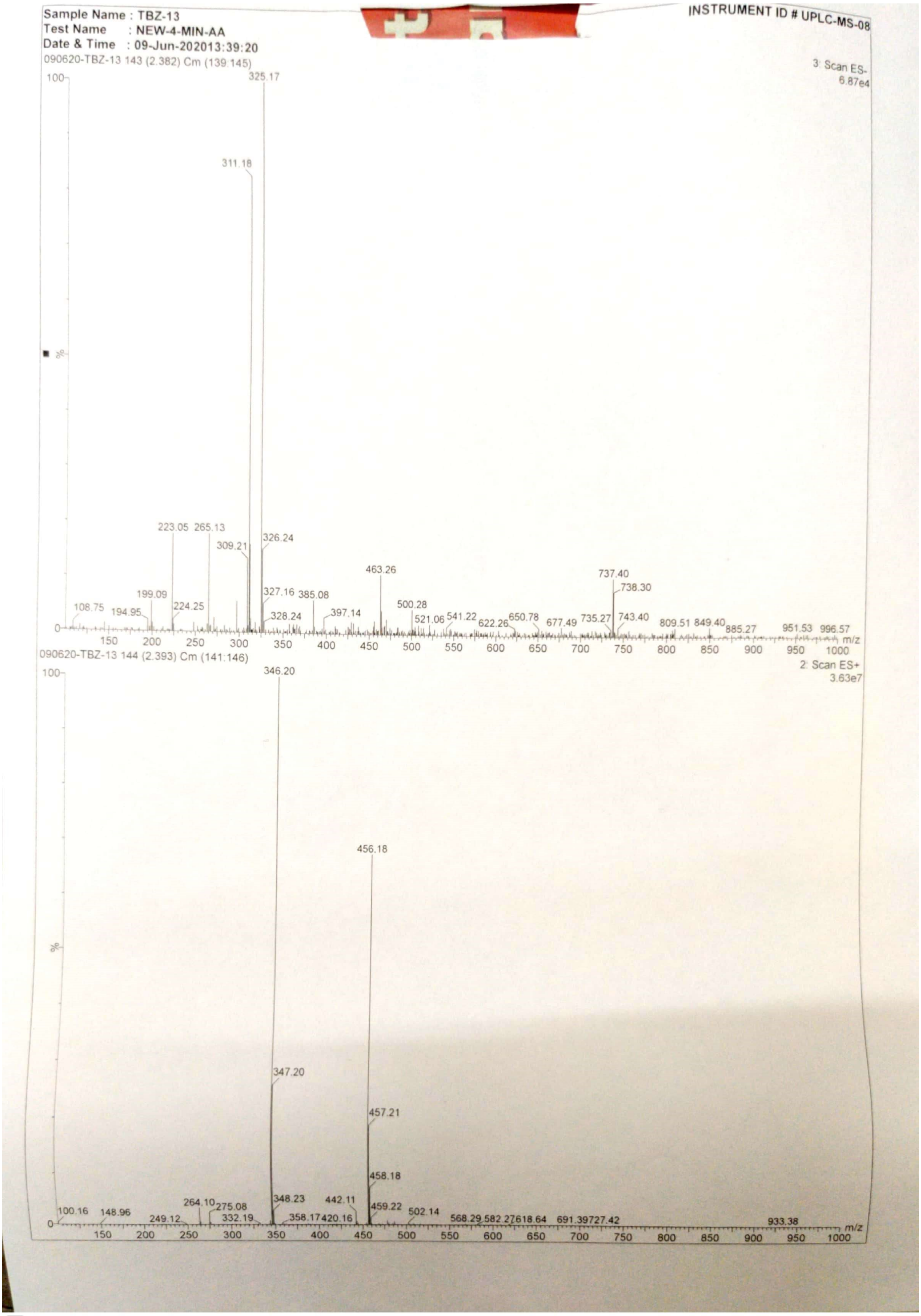

Scanned with CamScanner

Figure S39: Mass spectrum of Molecule (T13)

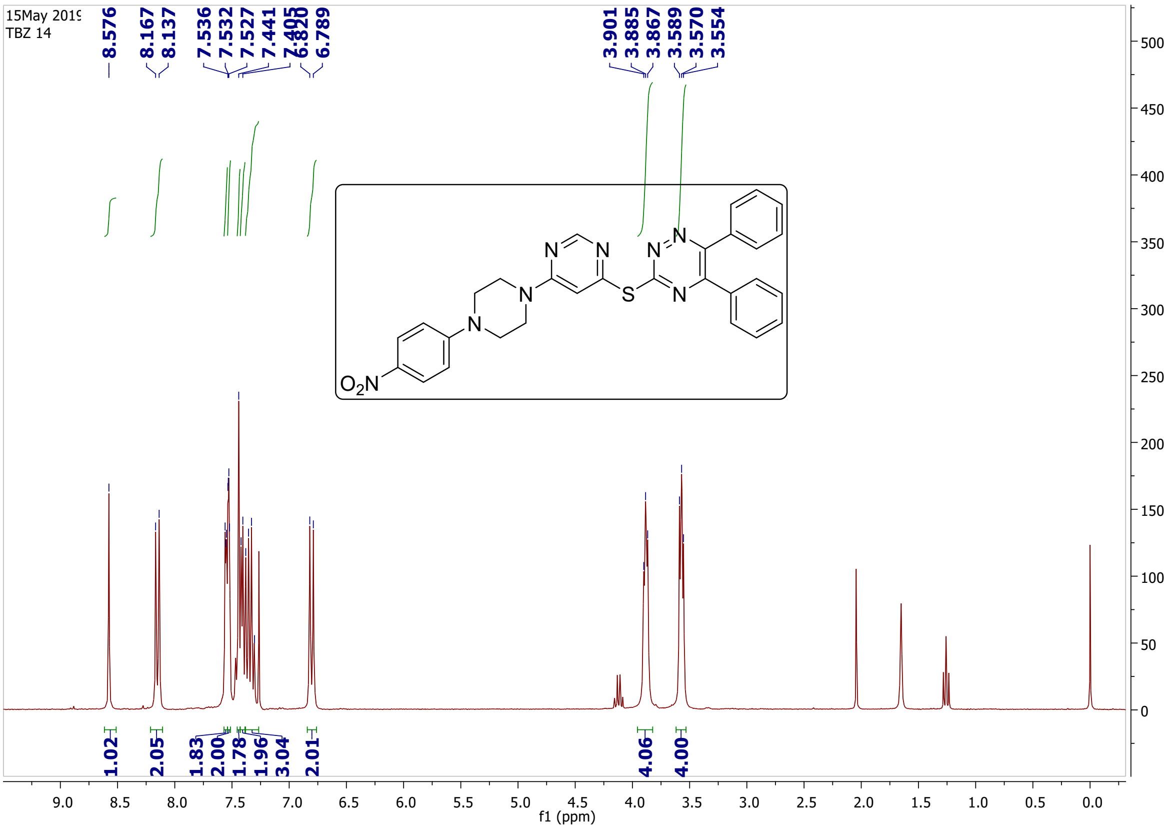

Figure S40: ^{1}H NMR spectrum of Molecule (T14)

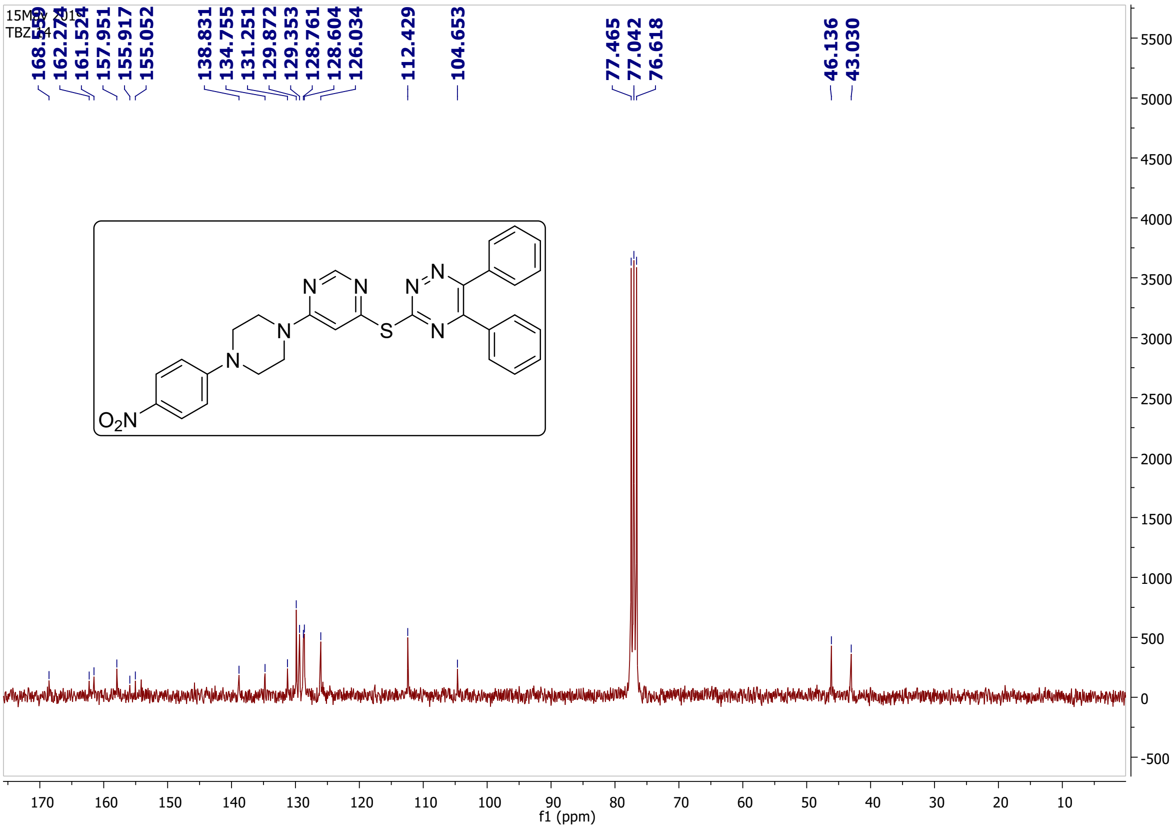

Figure S41: ^{13}C NMR spectrum of Molecule (T14)

TIC: from Sample 49 (TBZ-14) of 020519.wiff (Turbo Spray) Max. 3.2e8 cps.

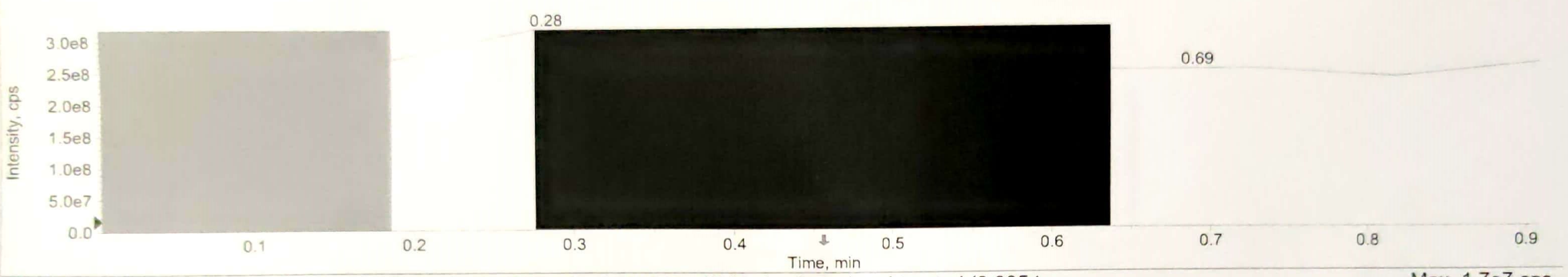

+Q1: Exp 1, 0.276 to 0.637 min from Sample 49 (TBZ-14) of 020519.wiff (Turbo Spray), subtracted (0.005 to ... Max. 1.7e7 cps.

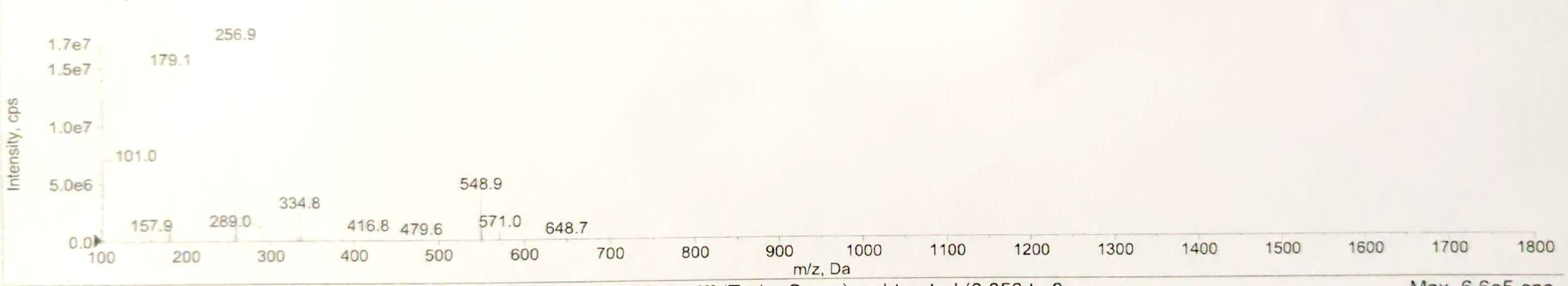

-Q1: Exp 2, 0.321 to 0.682 min from Sample 49 (TBZ-14) of 020519.wiff (Turbo Spray), subtracted (0.050 to 0... Max. 6.6e5 cps.

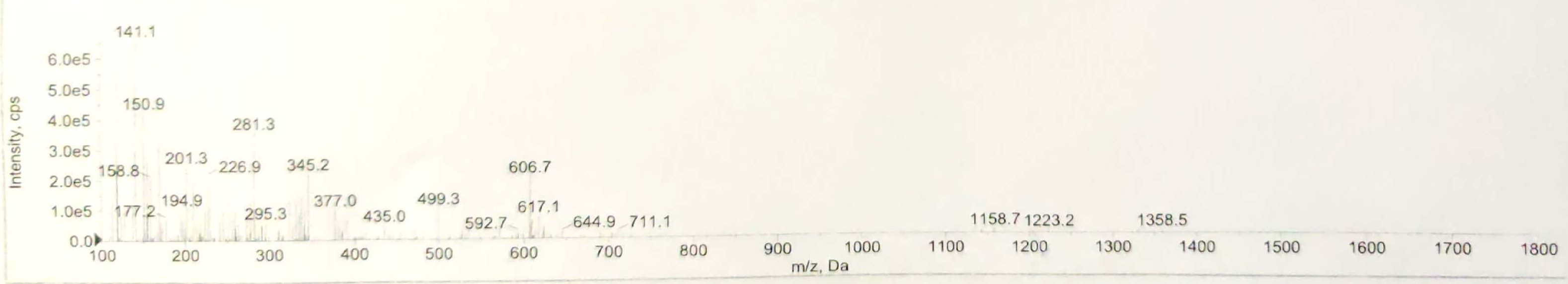

Scanned with CamScanner

Figure S42: Mass spectrum of Molecule (T14)

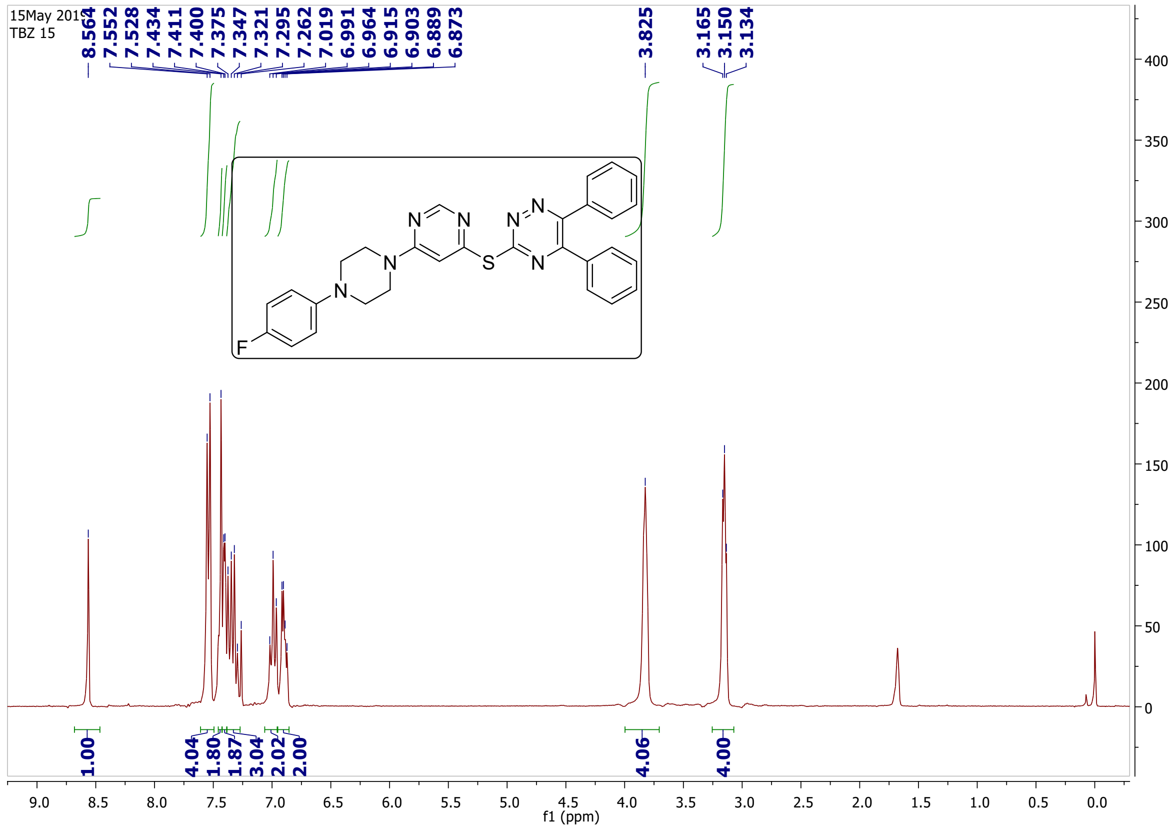

Figure S43: ^{1}H NMR spectrum of Molecule (T15)

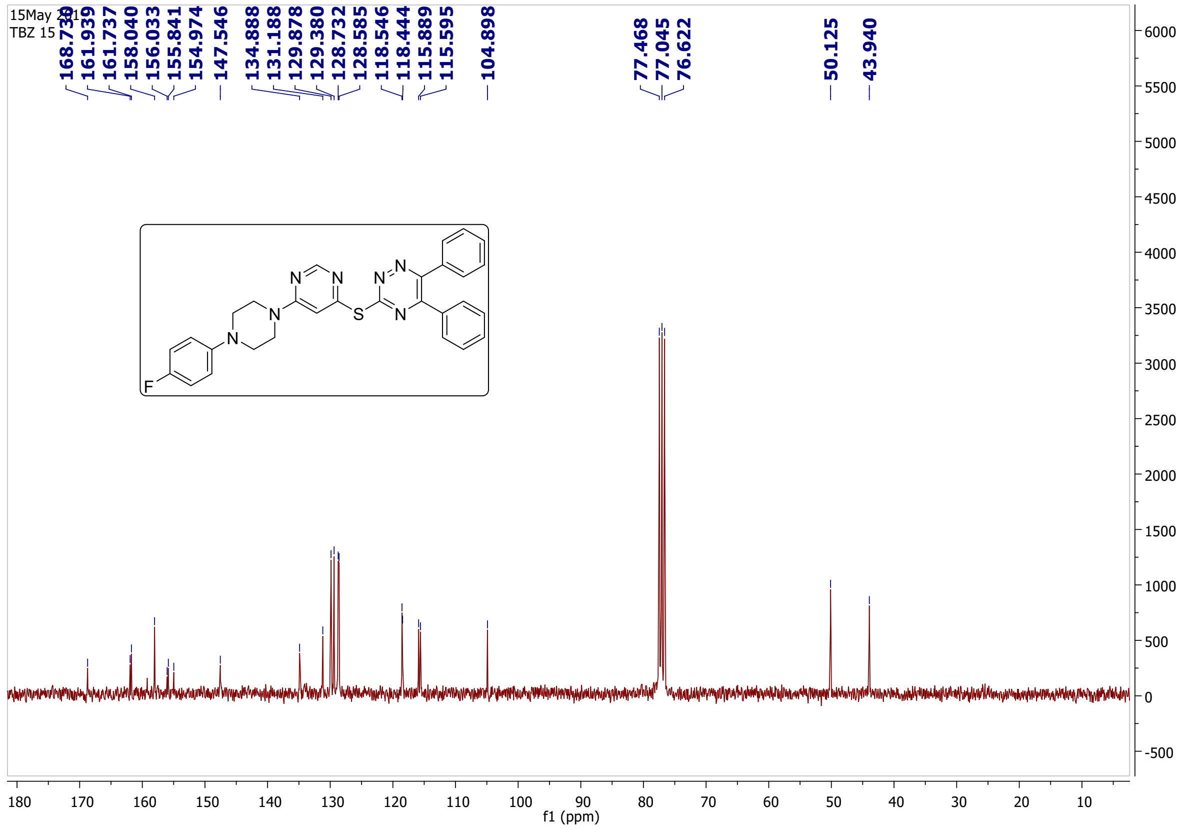

Figure S44: ^{13}C NMR spectrum of Molecule (T15)

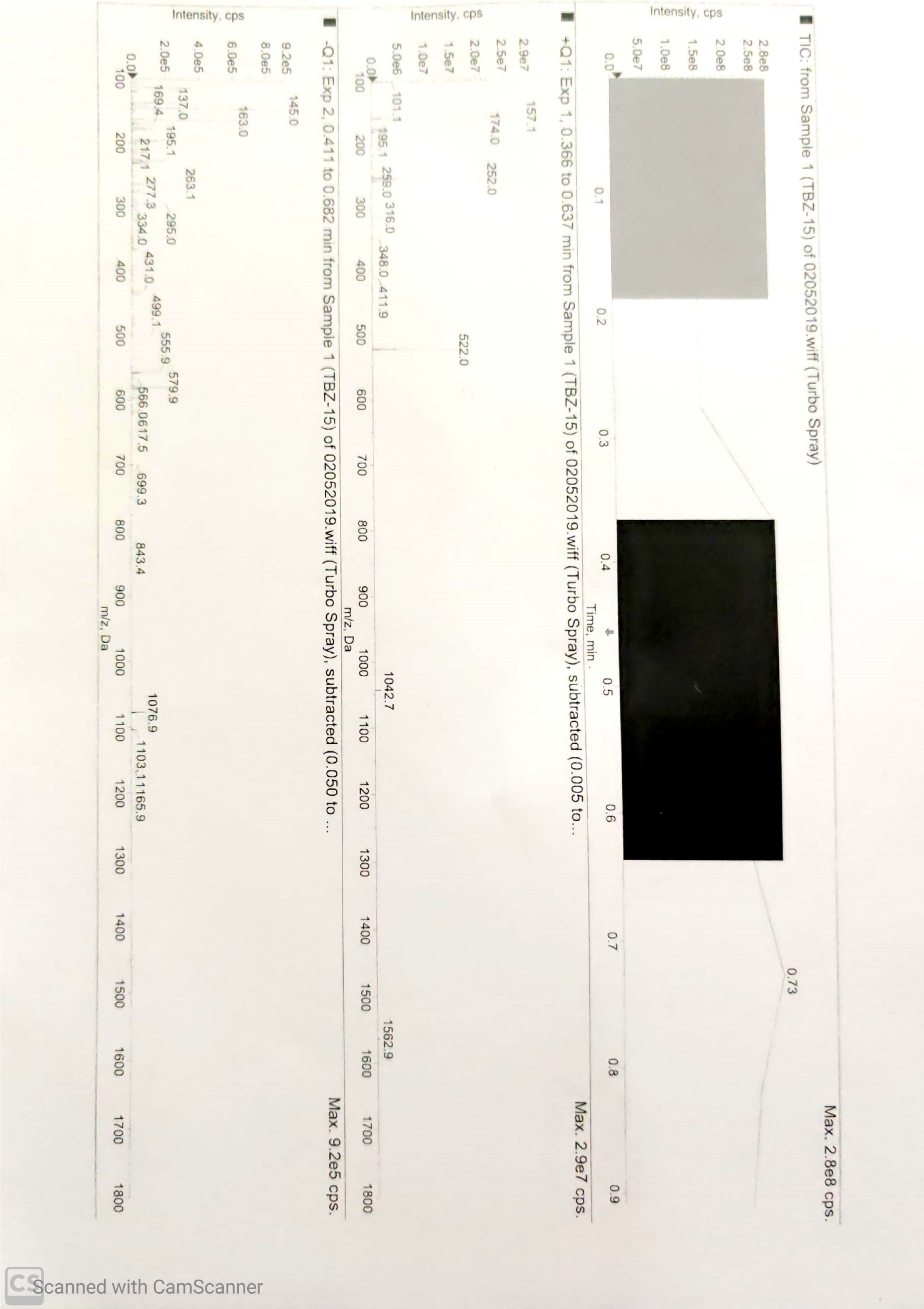

Scanned with CamScanner

Figure S45: Mass spectrum of Molecule (T15)

References

[1] J. Kumar, P. Meena, A. Singh, E. Jameel, M. Maqbool, M. Mobashir, et al., Synthesis and screening of triazolopyrimidine scaffold as multi-functional agents for Alzheimer's disease therapies, European Journal of Medicinal Chemistry. (2016). doi:10.1016/j.ejmech.2016.04.053.
